# Phase I pharmacokinetic study of single agent trametinib in patients with advanced cancer and hepatic dysfunction

**DOI:** 10.1186/s13046-021-02236-7

**Published:** 2022-02-07

**Authors:** Pei Jye Voon, Eric X. Chen, Helen X. Chen, Albert C. Lockhart, Solmaz Sahebjam, Karen Kelly, Ulka N. Vaishampayan, Vivek Subbiah, Albiruni R. Razak, Daniel J. Renouf, Sebastien J. Hotte, Arti Singh, Philippe L. Bedard, Aaron R. Hansen, S. Percy Ivy, Lisa Wang, Lee-Anne Stayner, Lillian L. Siu, Anna Spreafico

**Affiliations:** 1 Princess Margaret Cancer Centre, University of Toronto, 700 University Avenue, office 7-624, ON Toronto, Canada; 2 Cancer Therapy Evaluation Program, National Cancer Institute, Organ Dysfunction Working Group, MD Bethesda, USA; 3 Washington University in St. Louis, St. Louis, MO USA; 4 Moffitt Cancer Center, Tampa, FL USA; 5 UC Davis Comprehensive Cancer Center, Sacramento, CA USA; 6 Karmanos Cancer Institute, Detroit, MI USA; 7 MD Anderson Cancer Center, Houston, TX USA; 8 BC Cancer, Vancouver, BC Canada; 9 Juravinski Cancer Centre, Hamilton, ON Canada

**Keywords:** Trametinib, Phase I trial, Dose escalation, Hepatic dysfunction, Pharmacokinetics

## Abstract

**Background:**

Trametinib is an oral MEK 1/2 inhibitor, with a single agent recommended phase 2 dose (RP2D) of 2 mg daily (QD). This study was designed to evaluate RP2D, maximum tolerated dose (MTD), and pharmacokinetic (PK) profile of trametinib in patients with advanced solid tumors who had various degrees of hepatic dysfunction (HD).

**Methods:**

Advanced cancer patients were stratified into 4 HD groups based on Organ Dysfunction Working Group hepatic function stratification criteria: normal (Norm), mild (Mild), moderate (Mod), severe (Sev). Dose escalation was based on “3 + 3” design within each HD group. PK samples were collected at cycle 1 days 15-16.

**Results:**

Forty-six patients were enrolled with 44 evaluable for safety [Norm=17, Mild=7, Mod (1.5 mg)=4, Mod (2 mg)=5, Sev (1 mg)=9, Sev (1.5 mg)=2] and 22 for PK analysis. Treatment related adverse events were consistent with prior trametinib studies. No treatment related deaths occurred. Dose limiting toxicities (DLTs) were evaluable in 15 patients (Mild=6, Mod (1.5 mg)=3, Mod (2 mg)=2, Sev (1 mg)=3 and Sev (1.5 mg)=1). One DLT (grade 3 acneiform rash) was observed in a Sev patient (1.5 mg). Dose interruptions or reductions due to treatment related adverse events occurred in 15 patients (34%) [Norm=9, 53%; Mild=2, 29%; Mod (1.5 mg)=1, 33%; Mod (2 mg)=2, 33%; Sev (1 mg)=1, 11%; Sev (1.5 mg)=1; 50%]. There were no significant differences across HD groups for all PK parameters when trametinib was normalized to 2 mg. However, only limited PK data were available for the Mod (n = 3) and Sev (n = 3) groups compared to Norm (n = 10) and Mild (n = 6) groups. Trametinib is heavily protein bound, with no correlation between serum albumin level and unbound trametinib fraction (p = 0.26).

**Conclusions:**

RP2D for trametinib in Mild HD patients is 2 mg QD. There are insufficient number of evaluable patients due to difficulty of patient accrual to declare RP2D and MTD for Mod and Sev HD groups. DLTs were not observed in the highest dose cohorts that reached three evaluable patients – 1.5 mg QD in Mod group, and 1 mg QD in Sev group.

**Trial registration:**

This study was registered in the ClinicalTrials.gov website (NCT 02070549) on February 25, 2014. .

**Supplementary Information:**

The online version contains supplementary material available at 10.1186/s13046-021-02236-7.

## Background

Trametinib (Mekinist ®) is an orally bioavailable, highly selective and reversible allosteric inhibitor of MEK1/2 [1, 2]. Trametinib is currently approved for monotherapy and in combination with dabrafenib for the treatment of patients with unresectable/metastatic melanoma harbouring *BRAF V600* mutation. It is also indicated in combination with dabrafenib for adjuvant treatment of patients with Stage III melanoma following complete resection, advanced non-small cell lung cancer, and locally advanced or metastatic anaplastic thyroid cancer with *BRAF V600* mutation [3].

Trametinib is metabolized predominantly via deacetylation followed by oxidation and/or glucuronidation. Following administration, trametinib and its metabolites are excreted in the feces (≥81%) and to a minor extent in urine (≤19%) [4]. Dose selection for phase II and III clinical trials with trametinib was based on the results from its phase I study in which daily doses ranging from 0.125 to 4 mg were administered to patients with solid tumors. A dose of 2 mg administered once daily was selected based on tolerability, exposure-response relationship with pharmacodynamic markers in tumor biopsies, and clinical activity [5]. A population PK analysis showed that trametinib oral clearance and exposure were not significantly different in patients with mild hepatic impairment from those with normal hepatic function [6]. With the exception of this limited evaluation, there are no other prospective clinical and pharmacokinetic data available on trametinib in patients with hepatic dysfunction.

The NCI Organ Dysfunction Working Group (ODWG) was established to evaluate the safety and pharmacological profiles of approved anticancer agents wherein the absorption, distribution, metabolism and excretion processes are potentially variable in patients with organ dysfunction. This study addresses the dosing and PK analysis of trametinib in a special population of patients with hepatic dysfunction, generally excluded from studies as dosing and/or scheduling are unknown and patients are considered too frail to tolerate treatment. As an important post-marketing requirement, the current study, supported by the NCI ODWG, was developed to determine the maximum tolerated dose (MTD), dose-limiting toxicity (DLT) and pharmacokinetic (PK) profile of trametinib in advanced solid tumors patients with varying degrees of hepatic dysfunction.

## Methods

### Patient selection

Patients aged 18 or older with histologically or cytologically confirmed metastatic or unresectable solid tumors (except for hepatocellular carcinoma for which histological or cytological confirmation was not required) no longer suitable for standard curative or palliative treatments, or for whom standard therapy did not exist, were eligible for the study. Due to limited trametinib benefit, patients with pancreatic, colorectal cancer and patients with *BRAF V600E-*mutated melanoma who had progressed on BRAF inhibitor were excluded from the normal and mild hepatic dysfunction groups, but were permitted to enrol in the moderate and severe hepatic dysfunction groups. This exception was considered reasonable provided these patients with very limited options of treatment were adequately informed that their chance of benefit from trametinib was low and objective responses had been rarely observed. ECOG performance status ≤2 with life expectancy of greater than 3 months and adequate organ functions, except liver function, were required. Additional criteria include ability to swallow and absence of clinically significant gastrointestinal abnormalities. History of interstitial lung disease or pneumonitis, retinal vein occlusion, and significant cardiac comorbidities were key exclusion criteria. Complete eligibility criteria are provided in the trial protocol in Supplementary Appendix. The sample size planned for this study ranged between a minimum of 27 and a maximum of 68 patients. This multicenter NCI ODWG study (NCI protocol no. 9591), led by the Princess Margaret Phase I Consortium (currently known as North American Star Consortium) and supported by the Experimental Therapeutics Clinical Trials Network, was approved by regulatory and independent ethics committee at all participating sites. This study was registered in the ClinicalTrials.gov website (NCT 02070549).

This trial was sponsored by the US National Cancer Institute (NCI), Division of Cancer Treatment and Diagnosis (DCTD). Trametinib was supplied by Novartis under a Cooperative Research and Development Agreement (CRADA) with NCI DCTD.

### Study design and dosing

In this single-arm, dose finding, phase I clinical trial of single agent trametinib, advanced cancer patients with varying degrees of hepatic dysfunction were stratified into 4 groups (Norm: normal, Mild: mild, Mod: moderate, Sev: severe) according to their liver function tests based on ODWG hepatic function criteria as summarized in Table 1. Patients had to meet both total bilirubin and aspartate aminotransferase (AST) criteria to be included in a group. However, if a patient’s total bilirubin level or AST level were classified into different liver dysfunction groups, the patient was to be enrolled in the group with the highest degree of liver dysfunction. A patient’s hepatic dysfunction group assignment could be altered after registration if liver function tests performed within 24 h of starting trametinib changed from results obtained at the time of study registration. Patients in Norm group were included in this study as control patients and were followed for toxicity. They were not evaluable for DLT because the MTD has already been defined in this population.

**Table 1:** ODWG hepatic function criteria and dose escalation schema for each cohort as defined by hepatic function

	Group Norm	Group Mild	Group Mod	Group Sev
	**Normal** **hepatic function**	**Mild** **hepatic dysfunction**	**Moderate** **hepatic dysfunction**	**Severe** **Hepatic dysfunction**
**ODWG hepatic function criteria**	Bil ≤ ULN AST ≤ ULN	B1: bil ≤ULN and AST > ULN B2: ULN <bil ≤ 1.5x ULN and any AST	1.5x ULN < bil ≤ 3x ULN and any AST	3x ULN < bil ≤ 10x ULN and any AST
**Dose** **Level**	mg	mg	mg	mg
Level - 2	-	1	0.5	-
Level - 1	-	1.5	1	0.5
Level 1	2	2	1.5	1
Level +1	no escalation	no escalation	2	1.5
Level +2	no escalation	no escalation	no escalation	2
*Abbreviation: AST* Aspartate aminotransferase; *Bil* bilirubin; *ODWG* Organ Dysfunction Working Group				

Trametinib was administered orally once a day (QD) on a 28-day cycle schedule. Treatment was continued until progressive disease, unacceptable toxicity, or consent withdrawal. Dose escalation was adapted from standard 3 + 3 design except for Norm group. The design was modified to allow patients to enroll at higher dose levels before all 3 patients had cleared DLT evaluation as the clinical stability of patients with impaired hepatic function is limited. Norm, Mild, Mod and Sev group were also opened concurrently to optimize enrollment. Trametinib starting dose (dose level 1: DL1) varied based on hepatic dysfunction (Table 1). No dose escalation was planned for patients with Norm and Mild group. De-escalation to DL-1 was planned in all groups except Norm group. Although dose finding was carried out independently for each of the hepatic dysfunction groups, accrual to Sev group occurred in sequential single patient cohorts to limit the number of patients at risk of toxicity and patients were staggered until the first patient completed cycle 1 and so on for each subsequent patient. This one-by-one rule was applied only while the enrollment to the same dose level in the Mod group was incomplete; once completed, enrollment to the Sev group could occur without staggering.

The National Cancer Institute Common Terminology Criteria for Adverse Events (NCI CTCAE) version 4.0 was used to grade treatment-related toxicity. Dose-limiting toxicity (DLT) was defined as toxicity occurring during cycle 1 that was assessed to be possibly, probably or definitely related to the study drug. DLT criteria included: grade ≥3 nonhematologic toxicity (except allergic reactions, alopecia, grade ≥3 diarrhea, nausea, or vomiting responsive to supportive care and or grade ≥3 electrolyte toxicity that was corrected to grade 1 or baseline within 48 h), grade 4 neutropenia or thrombocytopenia, any febrile neutropenia or grade 3 thrombocytopenia complicated by haemorrhage. Worsening liver function, as defined by a rise in serum bilirubin not related to tumor progression or stent occlusion for 1 week or longer, was also considered a DLT if a patient’s bilirubin in the Mild group progressed into the severe dysfunction level; or if a patient’s bilirubin in the Mod group has increased from baseline to ≥ 3 fold; or if a patient’s bilirubin in Sev group increased from baseline to ≥ 2 fold. Other DLTs included treatment-related toxicities that resulted in failure to receive ≥ 75% of trametinib doses in cycle 1 despite maximal supportive care measures and delays in starting cycle 2 by ≥ 2 weeks due to treatment-related toxicity.

### Study assessment

Baseline evaluations included routine history and physical examination including dermatologic examination, complete blood count, serum chemistries, electrocardiogram, and computed tomogram (CT) and/or magnetic resonance imaging (MRI) of thorax, abdomen and pelvis. Liver function tests were done within 24 h prior to starting cycle 1 day 1. Ophthalmology examination was required at baseline and when clinically indicated during study because of the risk of trametinib-induced serous retinopathy. Electrocardiograms and echocardiograms/multi-gated acquisition (MUGA) scans to evaluate left ventricular ejection fraction were performed every 12 weeks.

### Pharmacokinetic evaluation

PK studies were planned for all enrolled patients. The trametinib half-life in patients with normal liver function is approximately 4 days and PK sampling was performed at cycle 1 day 15-16 for all patients such that trametinib was at or near the steady state. Blood samples were collected on day 15 of cycle 1 before and at 0.5, 1, 2, 3, 4, 6, 10 and 24 h following trametinib administration. Plasma trametinib concentrations were determined by Covance Laboratories Inc. (Madison, WI) using a validated LC/MS/MS assay. PK evaluable patients were defined as those who had all protocol required blood samples collected and in whom trametinib was administered as per protocol requirements without dose modification prior to PK blood sample collection. PK unevaluable patients were replaced to ensure adequate PK data for each group. In patients with incomplete PK data from cycle 1 and in those whose dose level or hepatic dysfunction group was changed between cycles, repeat PK sampling was allowed in subsequent cycles. For the Sev group, patients who completed protocol required PK assessment at day 16 were considered evaluable for DLT assessment in contrast to Mild and Mod groups in which patients were only DLT evaluable after a full 28-day cycle schedule, unless they developed DLT during cycle 1.

### Statistical considerations

The primary objectives include providing appropriate dosing recommendations, to establish the MTD and DLT as well as to characterize the PK profile of trametinib in advanced cancer patients with hepatic dysfunction (Mild, Mod, Sev groups). The secondary objectives include evaluation of safety, tolerability and antitumor activity associated with trametinib treatment in these patients.

Summary statistics, such as mean, median, proportion and laboratory values, were used to describe patients’ clinical characteristics. Objective response to treatment was assessed using RECIST 1.1 [7]. Progression free survival (PFS) was evaluated using Kaplan Meier analysis and log rank test was used to assess the difference between 4 hepatic function groups. Frequency and severity of adverse events were tabulated using counts and proportions detailing frequently occurring, serious and severe events of interest.

For PK analysis, PK parameters for trametinib including maximum plasma concentration (C_max_), minimum plasma concentration (C_min_), average plasma concentration (C_avg_), area under the plasma concentration–time curve 0-24 h (AUC_0 − 24_) and apparent oral clearance at steady state (CLss/F) were obtained using non-compartmental methods (Phoenix WinNonlin, version 8.3, Certara USA, Inc., Princeton, NJ). PK parameters among hepatic function groups were compared with analysis of variance (ANOVA) using GraphPad Prism (version 9.1.1, GraphPad Software, San Diego, CA).

For all statistical tests, two-sided tests were performed and no *p*-value adjustment was made due to the exploratory nature of these tests. A *p*-value of 0.05 or less will be considered statistically significant.

## Results

### Patient characteristics

**Fig. 1:**
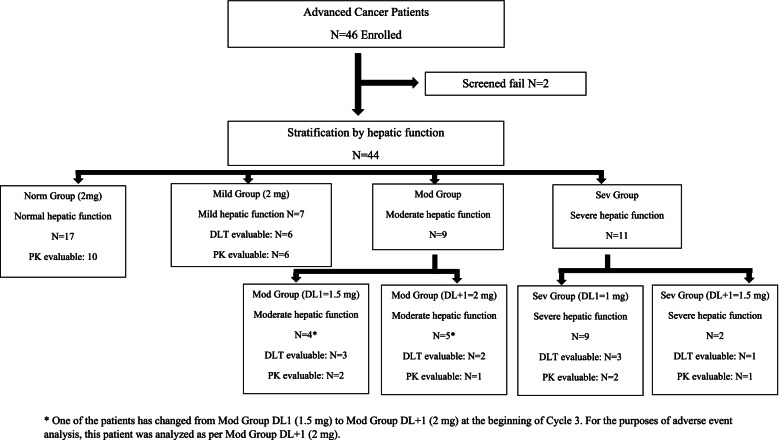
Study patients disposition based on hepatic function

Forty-six patients were enrolled in this study between March 2014 to December 2018, with 2 patients deemed ineligible (one patient from Norm group withdrew consent and one patient from Sev group had deteriorating performance status during screening period). The study closed to accrual due to challenges in enrolling patients with moderate and severe hepatic dysfunction due to rapid deterioration of the clinical status and inability to complete the requested collection of the PK samples. Forty-four patients (24 male, 20 female) were assigned to 1 of the 4 HD groups: Norm, n = 17; Mild, n = 7; Mod, n = 9; Sev, n = 11 (Fig. 1). The median age was 60 years (range, 27–77). The most common cancer types were biliary tract and pancreas (8 patients) followed by hepatocellular carcinoma (6 patients). One of the patients was escalated from 1.5 mg to 2 mg within Mod group at the beginning of cycle 3 due to improvement in liver function tests. Another patient was changed from Norm group to Mild group prior to receiving any treatment. For the purposes of adverse event analysis, these patients were analyzed as per their last assigned group. Additional demographic data are shown in Table 2.

**Table 2:** Baseline demographic and disease characteristic of patients (safety population, n = 44)

Baseline characteristic		Normal (Norm) (2 mg)	Mild (2 mg)	Moderate (Mod) DL1 (1.5 mg)	Moderate (Mod) DL+1 (2 mg)	Severe (Sev) DL1 (1 mg)	Severe (Sev) DL+1 (1.5 mg)	All Patients
**Number of patients**		**17**	**7**	**4**	**5**	**9**	**2**	**44**
**Age, median (range)**		60 (40, 76)	51 (27, 77)	63 (60, 73)	66 (39, 74)	63 (38, 74)	42.5 (42, 43)	60 (27, 77)
**Gender**	Female	11	4	0	2	2	1	20
	Male	6	3	4	3	7	1	24
**Performance status (ECOG)**	0	5	1	0	0	3	0	9
	1	12	5	4	4	6	2	33
	2	0	1	0	1	0	0	2
**Cancer Type**	Bile tract/Pancreas	0	0	2	1	4	1	8
	Liver	1	0	1	3	1	0	6
	Lung	4	1	0	0	0	0	5
	Uveal melanoma	3	1	0	0	0	0	4
	Genitourinary (Bladder/Urethral/ Prostate)	3	0	0	0	0	0	3
	Skin	1	1	0	0	1	0	3
	Breast	0	2	0	0	0	0	2
	Esophagus	0	1	0	0	1	0	2
	Ovary	2	0	0	0	0	0	2
	Thyroid	1	0	1	0	0	0	2
	Others	2	1	0	1	2	1	7
**Number of Prior Regimens**								
	≤2	4	4	2	3	2	0	15
	>2	13	3	2	2	7	2	29

### Safety and DLT

All 44 patients received at least one dose of trametinib. The median number of completed treatment cycles was highest in the Mod group (1.5 mg) with 3 cycles, followed by Norm group with 2 cycles, and 1 cycle each for Mild group, Mod group (1 mg) and Sev group (1 mg). The lowest median treatment cycle administered was in the Sev group (1.5 mg) at 0.5 cycles (Table 3).

**Table 3:** Trametinib Related Adverse Events ≥10% of study patients in descending order of frequency of occurrence

	Normal (Norm) (2 mg)			Mild (2 mg)			Moderate (Mod) DL1 (1.5 mg)			Moderate (Mod) DL+1 (2 mg)			Severe (Sev) DL1 (1 mg)			Severe (Sev) DL+1 (1.5 mg)			Total Patients
**Number of patients** **(Safety evaluable patients with at least one dose of trametinib)**	**17**			**7**			**3**			**6**			**9**			**2**			
**Median Cycle of Treatment Completed (Range)**	**2 (0, 18)**			**1 (0, 6)**			**3 (2, 3)**			**1 (0, 2)**			**1 (0, 5)**			**0.5 (0, 1)**			
**Adverse Event (Treatment Related), Grade**	**All**	**G1-2**	**G ≥ 3**	**All**	**G1-2**	**G ≥ 3**	**All**	**G1-2**	**G ≥ 3**	**All**	**G1-2**	**G ≥ 3**	**All**	**G1-2**	**G ≥ 3**	**All**	**G1-2**	**G ≥ 3**	
**Acneiform rash**	**9 (53%)**	**9 (53%)**	**0 (0%)**	**6 (86%)**	**6 (86%)**	**0 (0%)**	**2 (67%)**	**2 (67%)**	**0 (0%)**	**3 (50%)**	**3 (50%)**	**0 (0%)**	**1 (11%)**	**1 (11%)**	**0 (0%)**	**1 (50%)**	**0 (0%)**	**1 (50%)**	**22** **(50%)**
**Nausea**	**11 (65%)**	**11 (65%)**	**0 (0%)**	**2 (29%)**	**2 (29%)**	**0 (0%)**	**0 (0%)**	**0 (0%)**	**0 (0%)**	**2 (33%)**	**2 (33%)**	**0 (0%)**	**1 (11%)**	**1 (11%)**	**0 (0%)**	**1 (50%)**	**1 (50%)**	**0 (0%)**	**17** **(39%)**
**Diarrhea**	**9 (53%)**	**7 (41%)**	**2 (12%)**	**2 (29%)**	**2 (29%)**	**0 (0%)**	**1 (33%)**	**1 (33%)**	**0 (0%)**	**2 (33%)**	**2 (33%)**	**0 (0%)**	**2 (22%)**	**2 (22%)**	**0 (0%)**	**0 (0%)**	**0 (0%)**	**0 (0%)**	**16** **(36%)**
**Fatigue**	**10 (59%)**	**8 (47%)**	**2 (12%)**	**1 (14%)**	**1 (14%)**	**0 (0%)**	**0 (0%)**	**0 (0%)**	**0 (0%)**	**2 (33%)**	**2 (33%)**	**0 (0%)**	**0 (0%)**	**0 (0%)**	**0 (0%)**	**1 (50%)**	**0 (0%)**	**1 (50%)**	**14** **(32%)**
**Aspartate aminotransferase increased**	**10 (59%)**	**9 (53%)**	**1 (6%)**	**1 (14%)**	**1 (14%)**	**0 (0%)**	**0 (0%)**	**0 (0%)**	**0 (0%)**	**1 (17%)**	**1 (17%)**	**0 (0%)**	**1 (11%)**	**1 (11%)**	**0 (0%)**	**0 (0%)**	**0 (0%)**	**0 (0%)**	**13** **(30%)**
**Anemia**	**7** **(41%)**	**6 (35%)**	**1 (6%)**	**2 (29%)**	**2 (29%)**	**0 (0%)**	**0 (0%)**	**0 (0%)**	**0 (0%)**	**0 (0%)**	**0 (0%)**	**0 (0%)**	**1 (11%)**	**1 (11%)**	**0 (0%)**	**0 (0%)**	**0 (0%)**	**0 (0%)**	**10** **(23%)**
**Maculopapular rash**	**5 (29%)**	**3 (18%)**	**2 (12%)**	**2 (29%)**	**2 (29%)**	**0 (0%)**	**3 (100%)**	**3 (100%)**	**0 (0%)**	**0 (0%)**	**0 (0%)**	**0 (0%)**	**1 (11%)**	**1 (11%)**	**0 (0%)**	**0 (0%)**	**0 (0%)**	**0 (0%)**	**11** **(23%)**
**Thrombocytopenia**	**6 (35%)**	**5 (29%)**	**1 (6%)**	**0 (0%)**	**0 (0%)**	**0 (0%)**	**0 (0%)**	**0 (0%)**	**0 (0%)**	**2** **(33%)**	**2** **(33%)**	**0 (0%)**	**1 (11%)**	**1 (11%)**	**0 (0%)**	**1 (50%)**	**1 (50%)**	**0 (0%)**	**10** **(23%)**
**Alanine aminotransferase increased**	**5 (29%)**	**5 (29%)**	**0 (0%)**	**2 (29%)**	**1 (14%)**	**1 (14%)**	**0 (0%)**	**0 (0%)**	**0 (0%)**	**2 (33%)**	**2 (33%)**	**0 (0%)**	**0 (0%)**	**0 (0%)**	**0 (0%)**	**0 (0%)**	**0 (0%)**	**0 (0%)**	**9** **(20%)**
**Blood alkaline phosphatase increased**	**7 (41%)**	**7 (41%)**	**0 (0%)**	**2 (29%)**	**2 (29%)**	**0 (0%)**	**0 (0%)**	**0 (0%)**	**0 (0%)**	**0 (0%)**	**0 (0%)**	**0 (0%)**	**0 (0%)**	**0 (0%)**	**0 (0%)**	**0 (0%)**	**0 (0%)**	**0 (0%)**	**9** **(20%)**
**Hyponatremia**	**5 (29%)**	**3 (18%)**	**2 (12%)**	**2 (29%)**	**2 (29%)**	**0 (0%)**	**1 (17%)**	**1 (17%)**	**0 (0%)**	**0 (0%)**	**0 (0%)**	**0 (0%)**	**1 (11%)**	**1 (11%)**	**0 (0%)**	**0 (0%)**	**0 (0%)**	**0 (0%)**	**9** **(20%)**
**Vomiting**	**4 (24%)**	**4 (24%)**	**0 (0%)**	**4 (57%)**	**4 (57%)**	**0 (0%)**	**0 (0%)**	**0 (0%)**	**0 (0%)**	**0 (0%)**	**0 (0%)**	**0 (0%)**	**1 (11%)**	**1 (11%)**	**0 (0%)**	**0 (0%)**	**0 (0%)**	**0 (0%)**	**9** **(20%)**
**Hypertension**	**5 (29%)**	**4 (24%)**	**1 (6%)**	**2 (29%)**	**2 (29%)**	**0 (0%)**	**1 (33%)**	**1 (33%)**	**0 (0%)**	**0 (0%)**	**0 (0%)**	**0 (0%)**	**0 (0%)**	**0 (0%)**	**0 (0%)**	**0 (0%)**	**0 (0%)**	**0 (0%)**	**8** **(18%)**
**Hypomagnesemia**	**5 (29%)**	**5 (29%)**	**0 (0%)**	**3 (43%)**	**3 (43%)**	**0 (0%)**	**0 (0%)**	**0 (0%)**	**0 (0%)**	**0 (0%)**	**0 (0%)**	**0 (0%)**	**0 (0%)**	**0 (0%)**	**0 (0%)**	**0 (0%)**	**0 (0%)**	**0 (0%)**	**8** **(18%)**
**Decreased appetite**	**5 (29%)**	**5 (29%)**	**0 (0%)**	**0 (0%)**	**0 (0%)**	**0 (0%)**	**0 (0%)**	**0 (0%)**	**0 (0%)**	**2 (33%)**	**2 (33%)**	**0 (0%)**	**0 (0%)**	**0 (0%)**	**0 (0%)**	**0 (0%)**	**0 (0%)**	**0 (0%)**	**7** **(16%)**
**Lymphopenia**	**4 (24%)**	**3 (18%)**	**1 (6%)**	**1 (14%)**	**1 (14%)**	**0 (0%)**	**1 (33%)**	**1 (33%)**	**0 (0%)**	**0 (0%)**	**0 (0%)**	**0 (0%)**	**1 (11%)**	**0 (0%)**	**1 (11%)**	**0 (0%)**	**0 (0%)**	**0 (0%)**	**7** **(16%)**
**Peripheral Edema**	**4 (24%)**	**4 (24%)**	**0 (0%)**	**1 (14%)**	**1 (14%)**	**0 (0%)**	**1 (33%)**	**1 (33%)**	**0 (0%)**	**1 (17%)**	**1 (17%)**	**0 (0%)**	**0 (0%)**	**0 (0%)**	**0 (0%)**	**0 (0%)**	**0 (0%)**	**0 (0%)**	**7** **(16%)**
**Hypoalbuminemia**	**5 (29%)**	**4 (24%)**	**1 (6%)**	**0 (0%)**	**0 (0%)**	**0 (0%)**	**0 (0%)**	**0 (0%)**	**0 (0%)**	**1 (17%)**	**1 (17%)**	**0 (0%)**	**0 (0%)**	**0 (0%)**	**0 (0%)**	**0 (0%)**	**0 (0%)**	**0 (0%)**	**6** **(14%)**
**Mucositis**	**2 (12%)**	**2 (12%)**	**0 (0%)**	**2 (29%)**	**2 (29%)**	**0 (0%)**	**1 (33%)**	**1 (33%)**	**0 (0%)**	**1 (17%)**	**1 (17%)**	**0 (0%)**	**0 (0%)**	**0 (0%)**	**0 (0%)**	**0 (0%)**	**0 (0%)**	**0 (0%)**	**6** **(14%)**
**Cracked skin / fissure**	**4 (24%)**	**4 (24%)**	**0 (0%)**	**0 (0%)**	**0 (0%)**	**0 (0%)**	**0 (0%)**	**0 (0%)**	**0 (0%)**	**1 (17%)**	**1 (17%)**	**0 (0%)**	**0 (0%)**	**0 (0%)**	**0 (0%)**	**0 (0%)**	**0 (0%)**	**0 (0%)**	**5** **(11%)**
**Dysgeusia**	**4 (24%)**	**4 (24%)**	**0 (0%)**	**0 (0%)**	**0 (0%)**	**0 (0%)**	**1 (33%)**	**1 (33%)**	**0 (0%)**	**1 (17%)**	**1 (17%)**	**0 (0%)**	**0 (0%)**	**0 (0%)**	**0 (0%)**	**0 (0%)**	**0 (0%)**	**0 (0%)**	**5** **(11%)**
**Leucopenia**	**3 (18%)**	**3 (18%)**	**0 (0%)**	**0 (0%)**	**0 (0%)**	**0 (0%)**	**1 (33%)**	**1 (33%)**	**0 (0%)**	**0 (0%)**	**0 (0%)**	**0 (0%)**	**1 (11%)**	**1 (11%)**	**0 (0%)**	**0 (0%)**	**0 (0%)**	**0 (0%)**	**5** **(11%)**
**Dose interruption/reduction**	**9 (53%)**			**3 (43%)**			**3 (100%)**			**3 (50%)**			**3 (33%)**			**2 (100%)**			**23** **(52%)**
**Dose interruption/reduction due to TRAEs**	**9 (53%)**			**2 (29%)**			**1 (33%)**			**1 (11%)**			**1 (11%)**			**1 (50%)**			**15** **(43%)**
**Dose interruption/reduction due to non- TRAEs**	**0 (0%)**			**1(14%)**			**2 (66%)**			**1 (17%)**			**2 (22%)**			**1 (50%)**			**7** **(16%)**
**Treatment discontinuation due to TRAEs**	**1 (6%)**			**1 (14%)**			**0 (0%)**			**1 (33%)**			**0 (0%)**			**0 (0%)**			**3** **(7%)**
**Treatment discontinuation due to non-TRAEs**	**1 (6%)**			**0 (0%)**			**0 (0%)**			**3 (50%)**			**5 (56%)**			**1 (50%)**			**10** **(23%)**
**Death related to TRAEs**	**0 (0%)**			**0 (0%)**			**0 (0%)**			**0 (0%)**			**0 (0%)**			**0 (0%)**			**0** **(0%)**
**Any SAE**	**10 (59%)**			**4 (57%)**			**3 (100%)**			**5 (83%)**			**8 (89%)**			**2 (100%)**			**32** **(73%)**
**Treatment related SAE**	**7 (41%)**			**2 (29%)**			**1 (33%)**			**0 (0%)**			**0 (0%)**			**0 (0%)**			**10** **(23%)**

A total of 15 patients (56%) were DLT evaluable: Mild, n = 6; Mod (1.5 mg), n = 3; Mod (2 mg), n = 2; Sev (1 mg), n = 3; Sev (1.5 mg), n = 1. The common reasons of DLT non-evaluability comprised of dose interruption and/or modification due to treatment unrelated adverse events and disease progression before completion of DLT period (Supplementary Table S1). There were 5 DLT unevaluable patients [Mod (1.5 mg), n = 1; Mod (2 mg), n = 1 and Sev (1 mg), n = 3] because of dose interruption and/or modification secondary to treatment unrelated adverse events with most of these patients suffering from deterioration of hepatic function during DLT period. Disease progression prior to completion of DLT period had led to DLT non-evaluability for 4 patients in Mod (2 mg), n = 1 and Sev groups [Sev (1 mg), n = 2; Sev (1.5 mg), n = 1].

No DLT was identified in Mild group and thus a dose of 2 mg QD was considered to be safe and tolerable in patients with mild hepatic dysfunction. As only 2 patients were DLT-evaluable in Mod group (2 mg), it was not possible to declare an MTD for moderate hepatic dysfunction. However, there were no DLTs in the three DLT-evaluable patients treated at 1.5 mg, and the two DLT-evaluable patients treated at 2 mg. In Sev group (1 mg), 3 patients were DLT-evaluable, with no DLTs identified. There were only 2 patients enrolled in Sev (1.5 mg) group. The only DLT evaluable patient in this group was a grade 3 acneiform rash (DLT event) started on day 19 of first cycle which required dose interruption and resolved subsequently during cycle 2. Trametinib was resumed on cycle 2 day 20 at 1 mg but the patient’s disease subsequently progressed on cycle 3 day 8. Overall, for Sev group, all 3 DLT-evaluable patients treated at 1 mg reported no DLT, and one DLT-evaluable patient treated at 1.5 mg developed DLT as described above. Thus, it was not possible to declare an MTD for Sev group.

Dose interruptions, reductions and discontinuations are shown in Table 3. Dose interruptions or reductions due to TRAEs were higher in Norm group (53%) which included 17 patients. However, dose interruptions due to worsening symptoms of the underlying cancer and treatment unrelated AEs including worsening of liver function tests were higher in patients with increasing liver dysfunction: Norm, n = 0 (0%); Mild, n = 1 (14%); Mod (1.5 mg), n = 2 (66%); Mod (2 mg), n = 1 (17%); Sev (1 mg), n = 2 (22%); Sev (1.5 mg), n = 1 (50%). Treatment discontinuations from non-drug related AEs were also higher patients with more severe liver dysfunction at baseline: Norm, n = 1 (6%); Mild, n = 0 (0%); Mod (1.5 mg), n = 0 (0%); Mod (2 mg), n = 3 (50%); Sev (1 mg), n = 5 (56%); Sev (1.5 mg), n = 1 (50%). The most common reason for treatment discontinuation was disease progression (n = 23, 52.3%). No treatment related deaths were seen across all groups.

Table 3 (TRAEs in descending frequency) and Supplementary Table S2 (TRAEs by organ system) summarize the TRAEs across all 4 groups. The most frequent all grade TRAEs were acneiform rash (50%), followed by nausea (39%), diarrhea (36%), fatigue (32%) and aspartate aminotransaminase increased (30%). Grade 3 TRAEs were uncommon with 2 events in each of Mild, Sev (1 mg) and Sev (1.5 mg) liver dysfunction groups (grade 3 alanine aminotransferase and gamma-glutamyl transferase increased in Mild group; grade 3 lymphopenia and maculopapular rash in Sev (1 mg) group; grade 3 acneiform rash and fatigue in Sev (1.5 mg) group). In Norm group, there was 1 patient with grade 3 pneumonitis which required discontinuation of trametinib during cycle 1. It was not considered a DLT as Norm group was not evaluable for DLT. Overall, there were 10 patients with treatment related serious adverse events (SAEs). These SAEs were mainly from Norm group (7 in Norm group, 2 in Mild group, 1 in Mod (1.5 mg) group) which included three cases of serious rash (1 acneiform, 2 maculo-papular) and one event each for heart failure, hypotension, retinopathy, lung infection, hypoxia, pneumonitis, and acute kidney injury. There was no grade 3 or greater liver enzyme elevation and/or bilirubin elevation across all liver dysfunction groups except for 2 patients in Mild group: 1 patient had grade 3 alanine aminotransferase increased and subsequently resolved spontaneously in a week’s time, and another patient had grade 3 gamma-glutamyl transferase increased and was declared one week later to have clinical progression.

### Efficacy and tumor response

Secondary efficacy endpoint of tumor response was evaluable in 31 patients (70.4%): Norm, n = 13 (76%); Mild, n = 6 (86%); Mod (1.5 mg), n = 4 (100%); Mod (2 mg), n = 3 (60%); Sev (1 mg), n = 4 (44%); Sev (1.5 mg), n = 1 (50%) (Supplementary Table S3). Best response was stable disease (SD), with the following frequencies observed among the groups: Norm, n = 7 (53.8%); Mild, n = 2 (33.3%); Mod (1.5 mg), n = 3 (75%); Mod (2 mg), n = 2 (66%); Sev (1 mg), n = 3 (75%); Sev (1.5 mg), n = 0 (0%). The median duration (range) of SD were: Norm, 3.8 months (1.9-12.5 months); Mild, 4.8 months (3.0-6.6 months); Mod (1.5 mg), 3.5 months (3.0-3.5 months); Mod (2 mg), 2.4 months (2.3-2.4 months); Sev (1 mg), 3.4 months (2.7-4.6 months). There were no partial responses (PRs) noted in any of the hepatic dysfunction groups, compared to Norm group where 2 patients (14%) achieved PR (ovarian and non-small cell lung cancers). Supplementary Figure S1 shows the Kaplan Meier PFS curve comparing all 4 groups. The median PFS was 3.62 months for Norm group, 1.74 months for Mild group, 3.45 months for Mod group and 2.11 months for Sev group, respectively (*p*=0.12).

### Pharmacokinetics

Twenty-six patients had complete protocol required PK collections and 22 patients with complete PK sample collection were considered PK evaluable as trametinib was administered according to protocol without dose modification or omission (Norm=10, Mild=6, Mod=3, Sev=3) (Fig. 1). The reasons for PK non-evaluability were broadly divided into dose interruption and/or modification due to TRAEs (n = 8), treatment unrelated adverse events (n = 6) and disease progression (n = 4) prior to the PK sample collection (Supplementary Table S4).

**Fig. 2:**
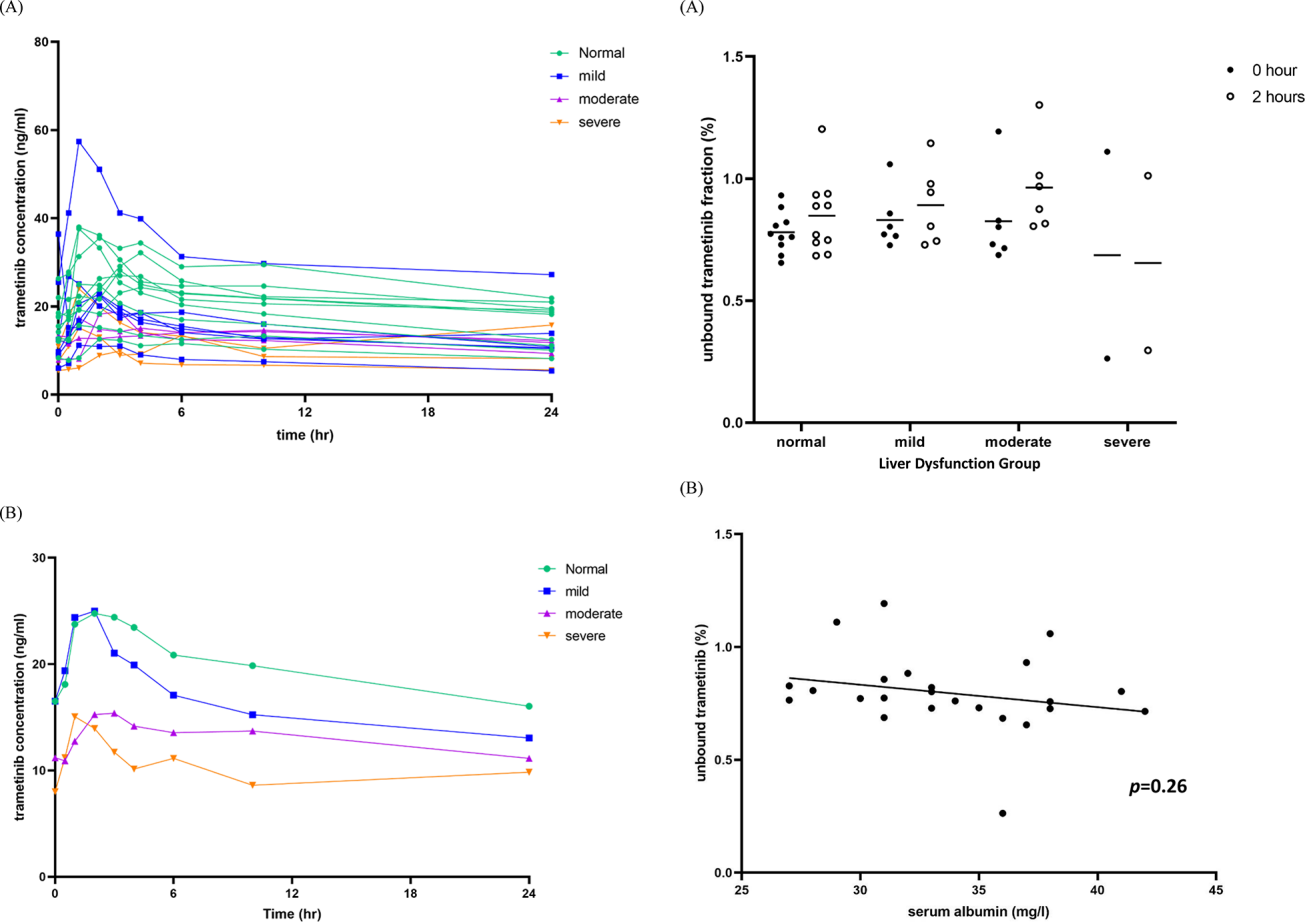
a. Trametinib concentration-time by hepatic dysfunction group (dose standardized to 2 mg) (n = 22). (A) Individual concentration (B) Mean concentration. b. Unbound trametinib fractions (n = 24). (A) by liver function group and time (B) Linear Regression of Unbound Trametinib Fraction vs. Serum Albumin for Protein Binding Analysis Population (p = 0.26)

Individual and mean trametinib concentration-time curves by hepatic function groups are shown in Fig. 2a (A) and (B) respectively with lower trametinib concentration over time detected in Sev and Mod as compared to Norm and Mild groups. PK parameters (C_max_, C_min_, C_avg_ and AUC_0 − 24_) with trametinib dose normalized to 2 mg were numerically lower in Mod and Sev in comparison to Norm and Mild groups (Table 4). However, these differences were not statistically different (Norm vs. Mild, Norm vs. Mod, Norm vs. Sev, Mild vs. Mod, Mild vs. Sev, Mod vs. Sev; all *p*=n.s.).

**Table 4:** Trametinib pharmacokinetic parameters with trametinib dose normalized to 2 mg for PK evaluable patients (n = 22)

	Descriptive Statistics	Normal Group Norm (n = 10)	Mild Group Mild (n = 6)	Moderate Group Mod (n = 3)	Severe Group Sev (n = 3)
**C**_**max**_ **(ng/mL)**	Geo mean (%CV) Mean (±SD)	26.2 (31.4) 27.6 (±8.7)	26.2 (53.8) 29.5 (±15.9)	16.8 (12.6) 16.9 (±2.1)	15.3 (43.6) 16.3 (±7.1)
**C**_**min**_ **(ng/mL)**	Geo mean (%CV) Mean (±SD)	14.4 (29.8) 15.1 (±4.5)	10.8 (57.3) 12.1 (±7.0)	10.4 (22.4) 10.5 (±2.4)	7.6 (32.3) 7.9 (±2.5)
**C**_**avg**_ **(ng/mL)**	Geo mean (%CV) Mean (±SD)	18.7 (28.1) 19.5 (±5.5)	14.7 (51.9) 16.2 (±8.4)	13.0 (8.1) 13.0 (±1.1)	9.5 (36.4) 10.0 (±3.6)
**AUC**_**0 − 24**_ (hr*ng/mL)	Geo mean (%CV) Mean (±SD)	449.5 (28.1) 468.8 (±132.0)	352.1 (51.9) 387.7 (±201.3)	311.0 (8.1) 311.7 (±25.2)	228.9 (36.4) 239.5 (±87.1)
**CLssF (mL/hr)**	Geo mean (%CV) Mean (±SD)	4449.0 (36.0) 4675.0 (±1683.6)	5680.0 (45.5) 6200.0 (±2818.6)	6431.6 (8.4) 6446.4(±542.6)	8736.3 (36.5) 9141.3 (±3337.1)
**Albumin level (g/L)**^a^ **n = 24**	Mean (±SD)	33.9 (±3.1)	34.2 (±5.6)	33.2 (±4.6)	32.5 (±4.9)

*Abbreviations: Geo mean* geometric mean; *%CV* coefficient of variation; *mean* arithmetic mean; *SD* standard deviation; *n* number of patients; *C*_max_ maximum concentration; *C*_min_ minimum concentration; *C*_avg_ average concentration; *AUC*_0− 24_ area under the plasma concentration–time curve 0-24 h; *CLssF* apparent oral clearance at steady state

^a^Serum albumin level of patients with trametinib unbound PK blood sample (PK evaluable and non-evaluable) n = 24

The percentage of unbound trametinib in plasma was 0.814 ± 0.048%, 0.861± 0.043%, 0.895 ± 0.097% and 0.67 ± 0.023% for Norm, Mild, Mod and Sev groups respectively (Fig. 2b (A)). No correlation between serum albumin and unbound trametinib fraction was detected by linear regression analysis (*p*=0.26) (Fig. 2b (B)).

## Discussion

Trametinib is active against a broad range of tumors, especially those harbouring *BRAF-V600* activating mutations [2, 8, 9]. Trametinib as monotherapy was shown to improve OS and PFS in comparison with standard dacarbazine or paclitaxel chemotherapy among patients with *BRAF* V600E or V600K mutated metastatic melanoma [10, 11]. This single-arm, dose finding, phase I trial reported trametinib safety, tolerability and PK in patients with advanced cancers having different degrees of hepatic dysfunction.

The safety results of this study confirmed the well characterized adverse event profile associated with trametinib [5, 12]. Reported TRAEs were primarily low grade and there were no new safety signals seen in both the normal and among hepatic dysfunction groups. Grade 3 or worse toxicities were identified mainly in the Norm group, possibly due to the larger number of patients recruited in this group compared to other hepatic dysfunction groups. In addition, median number of treatment cycles of Norm group was 2 in comparison to only 0.5 to 1 among all hepatic dysfunction group except for Mod (1.5 mg) group (n = 3 patients) which had median number of treatment cycle of 3. Thus, shorter duration of treatment exposure may also confound our observation of lower grade 3 or worse TRAEs among hepatic dysfunction groups in contrast to Norm group.

The clinical observation that trametinib was tolerable in patients with severe hepatic dysfunction was concordant with non-significant differences of all PK parameters among various liver function groups in addition to the lower dose of trametinib evaluated in these patients. PK parameters, such as C_max_, C_min_, C_avg_ and AUC_0 − 24_, were numerically lower while CLss/F was higher in the Sev group, suggesting that trametinib absorption may be impaired in the Sev group. The primary tumor sites of patients in the Sev group were mainly hepatobiliary in origin with background history of gastrointestinal co-morbidities including portal vein thrombosis, gastroesophageal reflux disease and prior history of total colectomy and ileostomy for ulcerative colitis. These conditions may have contributed to the impaired absorption of trametinib as reflected by the PK results and highlight the complex interplay between the pharmacological process of a drug with disease state and organ function.

A recent study investigated the effect of hepatic impairment on the PK parameters of another MEK inhibitor, cobimetinib, and demonstrated that patients with severe hepatic impairment had ∼30% lower total AUC_0−∞_ and ∼2-fold higher unbound AUC_0−∞_ compared to those with normal hepatic function [13]. There was no correlation between serum albumin and unbound fractions of trametinib in our study in contrast to the cobimetinib study. This discordance could be explained by the narrow range of serum albumin levels (range: 28 – 42 g/L, mean: 33.7 ± 4.2 g/L) in our trametinib study population. There were only 2 patients in the Sev group with serum albumin of 29 g/L and 36 g/L respectively, while a larger number of patients (n = 6) with lower mean serum albumin (27.3 ± 4 g/L) were enrolled in the severe hepatic impairment group of the cobimetinib study [13]. Admittedly, this analysis is confounded by the limited number of patients with severe hepatic dysfunction in both studies. Data on other small tyrosine kinase inhibitors including BRAF inhibitor dabrafenib in patients with renal and liver dysfunction (NCT01907802) have not been reported (https://clinicaltrials.gov/ct2/show/study/NCT01907802).

Slow accrual of patients, especially those in Sev group, was one of the major challenges in this study. There were only 2 patients recruited in this group at the 1.5 mg QD dose level, and one patient progressed during cycle one and was not DLT or PK evaluable; this limits any conclusions that can be drawn about the safety of trametinib in patients with severe liver dysfunction. Further recruitment was not possible for this cohort despite various pre-emptive measures to mitigate recruitment challenges, including multicenter participation, as well as allowing for evaluability of DLT after PK collection on day 16 in patients enrolled in the severe hepatic dysfunction cohort instead of waiting for the end of cycle 1 at day 28. In addition, eligibility criteria were relaxed to allow enrolment of patients with pancreatic cancer, colorectal cancer, and *BRAF V600E* mutant melanoma who had progressed on BRAF inhibitors in the Mod and Sev cohorts. A similar constraint was also noted in Mod group with only two DLT evaluable patients at 2 mg QD and the highest dose cohorts that reached three evaluable patients in Mod group was 1.5 mg QD. The recruitment of patients and the conduct of clinical trials focusing on organ dysfunctions are complex, and the establishment of organ dysfunction working group by the National Cancer Institute is to enable multicentre engagement and participation in such studies [14, 15]. Another potential concern in the conduct of studies with hepatic dysfunction is the limited duration of follow up of patients due to disease progression and deterioration of their underlying general condition [16]. This would adversely affect data collection of medium and longer term drug related adverse events. In short, innovative way of designing and conducting liver dysfunction studies should be further explored.

Another limitation of our hepatic dysfunction trial is that trametinib is infrequently used as monotherapy. Currently, most of the approved indications of trametinib are in combination with dabrafenib in line with the concept of simultaneous inhibition of two kinases in the MAPK pathway will produce a greater suppression of signal transduction than either inhibitor alone [17]. Furthermore, combination of MEK and RAF inhibition reduces the toxicities seen with monotherapy of either agents, especially the cutaneous toxicity related to BRAF inhibitors [18]. As this current study tested trametinib alone, the applicability of these results to dabrafenib and trametinib combination in the setting of hepatic dysfunction is unknown. This is particularly crucial in tumors that are known to present with hepatic dysfunction like biliary tract cancer. *BRAF V600* mutations are seen in 5% of this tumor type and combination of dabrafenib and trametinib treatment have shown promising activity in patients with *BRAF V600E*-mutated biliary tract cancer [19]. Nevertheless, this current study has described for the first time the safety and PK data for monotherapy trametinib with varying degree of hepatic dysfunction and will provide guidance for future clinical trial evaluating combination of trametinib with dabrafenib and other agents in similar population. In addition, it is well established that there is no worsening of toxicity observed from this combination in patients with normal liver function in large phase III studies [20–22].

## Conclusion 

In conclusion, the RP2D for trametinib in patients with mild hepatic dysfunction is 2 mg. However, there are insufficient number of evaluable patients to declare RP2D for moderate and severe hepatic dysfunction groups. DLTs were not observed in the highest dose cohorts that reached three evaluable patients – 1.5 mg QD in the Mod group, and 1 mg QD in the Sev cohorts. In addition, based on PK data, there are no significant differences between different hepatic function groups.

## Supplementary Information

**Additional file 1.**

**Additional file 2.**

**Additional file 3.**

## Data Availability

All data generated or analysed during this study are included in this published article and its supplementary information files.

## Abbreviations

ANOVA: analysis of variance
AST: aspartate aminotransferase
AUC_0 − 24_: area under the plasma concentration–time curve 0-24 h
C_avg_: average plasma concentration
CLss/F: apparent oral clearance at steady state
C_max_: maximum plasma concentration
C_min_: minimum plasma concentration
CRADA: Cooperative Research and Development Agreement
CT: computed tomogram
DCTD: Division of Cancer Treatment and Diagnosis
DLTs: Dose limiting toxicities
DL: dose level
HD: hepatic dysfunction
Mod: Moderate
MTD: maximum tolerated dose
MRI: magnetic resonance imaging
MUGA: multi-gated acquisition
NCI: National Cancer Institute
Norm: normal
ODWG: Organ Dysfunction Working Group
PK: pharmacokinetic
PFS: Progression free survival
QD: daily
RP2D: recommended phase 2 dose
SAE: serious adverse events
Sev: severe
TRAEs: Treatment related adverse events
