# Supplementary material for: Phase I pharmacokinetic study of single agent trametinib in patients with advanced cancer and hepatic dysfunction
Source: J Exp Clin Cancer Res. 2022 Feb 7;41:51. doi: 10.1186/s13046-021-02236-7 (PMC8819907; doi:10.1186/s13046-021-02236-7)
Supplement: Supplementary file 1 — Additional file 1. [file 13046_2021_2236_MOESM1_ESM.docx]

Supplementary Table S1. DLT evaluable patients and reasons of DLT non-evaluability

|  | Norm | Mild | Mod  (1.5mg) | Mod  (2mg) | Sev  (1mg) | Sev  (1.5mg) | Total |
| --- | --- | --- | --- | --- | --- | --- | --- |
| **Number of patients** | 17 | 7 | 4 | 5 | 9 | 2 | 44 |
| **DLT evaluable patients**  **(DLT period cycle 1=28 days)** | NA | 6 | 3 | 2 | 3 | 1 | 15 |
| **Reasons for DLT non-evaluability** | | | | | | | |
| 1. **Withdrawal of consent** | NA | 1 | 0 | 0 | 0 | 0 | 1 |
| 1. **Dose interruption/modification due to treatment unrelated AEs** | NA | 0 | 1 | 1 | 3 | 0 | 5 |
| 1. **Dose interruption/modification due to treatment related AEs** | NA | 0 | 0 | 1* | 0 | 0 | 1 |
| 1. **Disease progression** | NA | 0 | 0 | 1 | 2 | 1 | 4 |
| 1. **Others** | NA | 0 | 0 | 0 | 1** | 0 | 1 |

* 1 patient had dose interruption of trametinib of 2 mg for grade 2 treatment related fatigue by investigator for 6 days and treatment was resumed on cycle 1 day 15 with dose reduction to 1.5mg when fatigue had improved to grade 1

** 1 patient had elevation of total bilirubin at baseline (C1D1) of Sev group but rapidly resolved to normal bilirubin (Norm group criteria) throughout all the blood test timepoint in DLT period (Cycle 1)

Supplementary Table S2. Trametinib Related Adverse Events by Organ Systems

|  |  | **Normal (Norm)**  **(2 mg)** | | | **Mild**  **(2 mg)** | | | **Moderate (Mod) DL1**  **(1.5 mg)** | | | **Moderate (Mod) DL+1**  **(2 mg)** | | | **Severe (Sev) DL1**  **(1 mg)** | | | **Severe (Sev) DL+1**  **(1.5 mg)** | | |
| --- | --- | --- | --- | --- | --- | --- | --- | --- | --- | --- | --- | --- | --- | --- | --- | --- | --- | --- | --- |
| **Number of patients** |  | **17** | | | **7** | | | **3** | | | **6** | | | **9** | | | **2** | | |
| **Median Cycle of Treatment (Range)** |  | **2 (0, 18)** | | | **1 (0, 6)** | | | **3 (2, 3)** | | | **1 (0, 2)** | | | **1 (0, 5)** | | | **0.5 (0, 1)** | | |
| **Median day on treatment** |  | **56 (5, 506)** | | | **55 (9, 189)** | | | **97.5 (46, 111)** | | | **36 (7, 62)** | | | **35 (7, 112)** | | | **31.5 (10, 53)** | | |
| **Adverse Event (Treatment related)** |  | **All** | **G 1-2** | **G ≥3** | **All** | **G 1-2** | **G ≥3** | **All** | **G 1-2** | **G ≥3** | **All** | **G1-2** | **G ≥3** | **All** | **G1-2** | **G≥3** | **All** | **G1-2** | **G ≥3** |
| **Blood and Lymphatic System** |  | | | | | | | | | | | | | | | | | | |
|  | **Anemia** | 7  (41%) | 6  (35%) | 1  (6%) | 2 (29%) | 2 (29%) | 0  (0%) | 0  (0%) | 0  (0%) | 0  (0%) | 0  (0%) | 0  (0%) | 0  (0%) | 1 (11%) | 1 (11%) | 0  (0%) | 0  (0%) | 0  (0%) | 0  (0%) |
|  | **Thrombocytopenia** | 6 (35%) | 5 (29%) | 1  (6%) | 0  (0%) | 0  (0%) | 0  (0%) | 0  (0%) | 0  (0%) | 0  (0%) | 2  (33%) | 2  (33%) | 0  (0%) | 1 (11%) | 1 (11%) | 0  (0%) | 1 (50%) | 1 (50%) | 0  (0%) |
|  | **Lymphopenia** | 4 (24%) | 3 (18%) | 1  (6%) | 1 (14%) | 1 (14%) | 0  (0%) | 1 (33%) | 1 (33%) | 0  (0%) | 0  (0%) | 0  (0%) | 0  (0%) | 1 (11%) | 0  (0%) | 1 (11%) | 0  (0%) | 0  (0%) | 0  (0%) |
|  | **Neutropenia** | 3 (18%) | 3 (18%) | 0  (0%) | 0  (0%) | 0  (0%) | 0  (0%) | 1 (33%) | 1 (33%) | 0  (0%) | 0  (0%) | 0  (0%) | 0  (0%) | 0  (0%) | 0  (0%) | 0  (0%) | 0  (0%) | 0  (0%) | 0  (0%) |
|  | **Leucopenia** | 3 (18%) | 3 (18%) | 0  (0%) | 0  (0%) | 0  (0%) | 0  (0%) | 1 (33%) | 1 (33%) | 0  (0%) | 0  (0%) | 0  (0%) | 0  (0%) | 1 (11%) | 1 (11%) | 0  (0%) | 0  (0%) | 0  (0%) | 0  (0%) |
| **Cardiovascular** |  | | | | | | | | | | | | | | | | | | |
|  | **Hypertension** | 5 (29%) | 4 (24%) | 1  (6%) | 2 (29%) | 2 (29%) | 0  (0%) | 1 (33%) | 1 (33%) | 0  (0%) | 0  (0%) | 0  (0%) | 0  (0%) | 0  (0%) | 0  (0%) | 0  (0%) | 0  (0%) | 0  (0%) | 0  (0%) |
| **Gastrointestinal disorder** |  | | | | | | | | | | | | | | | | | | |
|  | **Nausea** | 11 (65%) | 11 (65%) | 0  (0%) | 2 (29%) | 2 (29%) | 0  (0%) | 0  (0%) | 0  (0%) | 0  (0%) | 2 (33%) | 2 (33%) | 0  (0%) | 1 (11%) | 1 (11%) | 0  (0%) | 1 (50%) | 1 (50%) | 0  (0%) |
|  | **Diarrhea** | 9 (53%) | 7 (41%) | 2 (12%) | 2 (29%) | 2 (29%) | 0  (0%) | 1 (33%) | 1 (33%) | 0  (0%) | 2 (33%) | 2 (33%) | 0  (0%) | 2 (22%) | 2 (22%) | 0  (0%) | 0  (0%) | 0  (0%) | 0  (0%) |
|  | **Vomiting** | 4 (24%) | 4 (24%) | 0  (0%) | 4 (57%) | 4 (57%) | 0  (0%) | 0  (0%) | 0  (0%) | 0  (0%) | 0  (0%) | 0  (0%) | 0  (0%) | 1 (11%) | 1 (11%) | 0  (0%) | 0  (0%) | 0  (0%) | 0  (0%) |
|  | **Dry mouth** | 2 (12%) | 2 (12%) | 0  (0%) | 1 (14%) | 1 (14%) | 0  (0%) | 0  (0%) | 0  (0%) | 0  (0%) | 0  (0%) | 0  (0%) | 0  (0%) | 0  (0%) | 0  (0%) | 0  (0%) | 0  (0%) | 0  (0%) | 0  (0%) |
|  | **Mucositis** | 2 (12%) | 2 (12%) | 0  (0%) | 2 (29%) | 2 (29%) | 0  (0%) | 1 (33%) | 1 (33%) | 0  (0%) | 1 (17%) | 1 (17%) | 0  (0%) | 0  (0%) | 0  (0%) | 0  (0%) | 0  (0%) | 0  (0%) | 0  (0%) |
| **General disorder** |  |  |  |  |  |  |  |  |  |  |  |  |  |  |  |  |  |  |  |
|  | **Fatigue** | 10 (59%) | 8 (47%) | 2 (12%) | 1 (14%) | 1 (14%) | 0  (0%) | 0  (0%) | 0  (0%) | 0  (0%) | 2 (33%) | 2 (33%) | 0  (0%) | 0  (0%) | 0  (0%) | 0  (0%) | 1 (50%) | 0  (0%) | 1  (50%) |
|  | **Decreased appetite** | 5 (29%) | 5 (29%) | 0  (0%) | 0  (0%) | 0  (0%) | 0  (0%) | 0  (0%) | 0  (0%) | 0  (0%) | 2 (33%) | 2 (33%) | 0  (0%) | 0  (0%) | 0  (0%) | 0  (0%) | 0  (0%) | 0  (0%) | 0  (0%) |
|  | **Peripheral edema** | 4 (24%) | 4 (24%) | 0  (0%) | 1 (14%) | 1 (14%) | 0  (0%) | 1 (33%) | 1 (33%) | 0  (0%) | 1 (17%) | 1 (17%) | 0  (0%) | 0  (0%) | 0  (0%) | 0  (0%) | 0  (0%) | 0  (0%) | 0  (0%) |
|  | **Pyrexia** | 2 (12%) | 2 (12%) | 0  (0%) | 0  (0%) | 0  (0%) | 0  (0%) | 0  (0%) | 0  (0%) | 0  (0%) | 0  (0%) | 0  (0%) | 0  (0%) | 0  (0%) | 0  (0%) | 0  (0%) | 0  (0%) | 0  (0%) | 0  (0%) |
|  | **Weight loss** | 2 (12%) | 2 (12%) | 0  (0%) | 0  (0%) | 0  (0%) | 0  (0%) | 0  (0%) | 0  (0%) | 0  (0%) | 1  (17%) | 1  (17%) | 0  (0%) | 0  (0%) | 0  (0%) | 0  (0%) | 0  (0%) | 0  (0%) | 0  (0%) |
|  | **Chills** | 1  (6%) | 1  (6%) | 0  (0%) | 0  (0%) | 0  (0%) | 0  (0%) | 1 (33%) | 1 (33%) | 0  (0%) | 0  (0%) | 0  (0%) | 0  (0%) | 0  (0%) | 0  (0%) | 0  (0%) | 0  (0%) | 0  (0%) | 0  (0%) |
| **Investigations** |  | | | | | | | | | | | | | | | | | | |
|  | **Aspartate aminotransferase increased** | 10 (59%) | 9 (53%) | 1  (6%) | 1 (14%) | 1 (14%) | 0  (0%) | 0  (0%) | 0  (0%) | 0  (0%) | 1 (17%) | 1 (17%) | 0  (0%) | 1 (11%) | 1 (11%) | 0  (0%) | 0  (0%) | 0  (0%) | 0  (0%) |
|  | **Blood alkaline phosphatase increased** | 7 (41%) | 7 (41%) | 0  (0%) | 2 (29%) | 2 (29%) | 0  (0%) | 0  (0%) | 0  (0%) | 0  (0%) | 0  (0%) | 0  (0%) | 0  (0%) | 0  (0%) | 0  (0%) | 0  (0%) | 0  (0%) | 0  (0%) | 0  (0%) |
|  | **Alanine aminotransferase increased** | 5 (29%) | 5 (29%) | 0  (0%) | 2 (29%) | 1 (14%) | 1 (14%) | 0  (0%) | 0  (0%) | 0  (0%) | 2 (33%) | 2 (33%) | 0  (0%) | 0  (0%) | 0  (0%) | 0  (0%) | 0  (0%) | 0  (0%) | 0  (0%) |
|  | **Hypoalbuminemia** | 5 (29%) | 4 (24%) | 1  (6%) | 0  (0%) | 0  (0%) | 0  (0%) | 1 (17%) | 1 (17%) | 0  (0%) | 0  (0%) | 0  (0%) | 0  (0%) | 0  (0%) | 0  (0%) | 0  (0%) | 0  (0%) | 0  (0%) | 0  (0%) |
|  | **Hypomagnesemia** | 5 (29%) | 5 (29%) | 0  (0%) | 3 (43%) | 3 (43%) | 0  (0%) | 0  (0%) | 0  (0%) | 0  (0%) | 0  (0%) | 0  (0%) | 0  (0%) | 0  (0%) | 0  (0%) | 0  (0%) | 0  (0%) | 0  (0%) | 0  (0%) |
|  | **Hyponatremia** | 5 (29%) | 3 (18%) | 2 (12%) | 2 (29%) | 2 (29%) | 0  (0%) | 1 (17%) | 1 (17%) | 0  (0%) | 0  (0%) | 0  (0%) | 0  (0%) | 1 (11%) | 1 (11%) | 0  (0%) | 0  (0%) | 0  (0%) | 0  (0%) |
| **Metabolism and Nutrition Disorder** |  | | | | | | | | | | | | | | | | | | |
|  | **Hypoglycemia** | 0  (0%) | 0  (0%) | 0  (0%) | 0  (0%) | 0  (0%) | 0  (0%) | 0  (0%) | 0  (0%) | 0  (0%) | 0  (0%) | 0  (0%) | 0  (0%) | 1 (11%) | 1 (11%) | 0  (0%) | 0  (0%) | 0  (0%) | 0  (0%) |
| **Musculoskeletal disorder** |  | | | | | | | | | | | | | | | | | | |
|  | **Muscle weakness upper limb** | 2 (12%) | 2 (12%) | 0  (0%) | 0  (0%) | 0  (0%) | 0  (0%) | 0  (0%) | 0  (0%) | 0  (0%) | 0  (0%) | 0  (0%) | 0  (0%) | 0  (0%) | 0  (0%) | 0  (0%) | 0  (0%) | 0  (0%) | 0  (0%) |
| **Neurological disorder** |  | | | | | | | | | | | | | | | | | | |
|  | **Dysgeusia** | 4 (24%) | 4 (24%) | 0  (0%) | 0  (0%) | 0  (0%) | 0  (0%) | 1 (33%) | 1 (33  %) | 0  (0%) | 1 (20%) | 1 (20%) | 0  (0%) | 0  (0%) | 0  (0%) | 0  (0%) | 0  (0%) | 0  (0%) | 0  (0%) |
| **Skin disorder** |  | | | | | | | | | | | | | | | | | | |
|  | **Acneiform rash** | 9 (53%) | 9 (53%) | 0  (0%) | 6 (86%) | 6 (86%) | 0  (0%) | 2 (67%) | 2 (67%) | 0  (0%) | 3 (50%) | 3 (50%) | 0  (0%) | 1 (11%) | 1 (11%) | 0  (0%) | 1 (50%) | 0  (0%) | 1 (50%) |
|  | **Maculopapular rash** | 5 (29%) | 3 (18%) | 2 (12%) | 2 (29%) | 2 (29%) | 0  (0%) | 3 (100%) | 3 (100%) | 0  (0%) | 0  (0%) | 0  (0%) | 0  (0%) | 1 (11%) | 1 (11%) | 0  (0%) | 0  (0%) | 0  (0%) | 0  (0%) |
|  | **Cracked skin / fissure** | 4 (24%) | 4 (24%) | 0  (0%) | 0  (0%) | 0  (0%) | 0  (0%) | 0  (0%) | 0  (0%) | 0  (0%) | 1 (17%) | 1 (17%) | 0  (0%) | 0  (0%) | 0  (0%) | 0  (0%) | 0  (0%) | 0  (0%) | 0  (0%) |
|  | **Paronychia** | 4 (24%) | 4 (24%) | 0  (0%) | 0  (0%) | 0  (0%) | 0  (0%) | 0  (0%) | 0  (0%) | 0  (0%) | 0  (0%) | 0  (0%) | 0  (0%) | 0  (0%) | 0  (0%) | 0  (0%) | 0  (0%) | 0  (0%) | 0  (0%) |
|  | **Papular rash** | 3 (18%) | 3 (18%) | 0  (0%) | 0  (0%) | 0  (0%) | 0  (0%) | 0  (0%) | 0  (0%) | 0  (0%) | 0  (0%) | 0  (0%) | 0  (0%) | 0  (0%) | 0  (0%) | 0  (0%) | 0  (0%) | 0  (0%) | 0  (0%) |
|  | **Dry skin** | 2 (12%) | 2 (12%) | 0  (0%) | 1 (14%) | 1 (14%) | 0  (0%) | 0  (0%) | 0  (0%) | 0  (0%) | 0  (0%) | 0  (0%) | 0  (0%) | 0  (0%) | 0  (0%) | 0  (0%) | 0  (0%) | 0  (0%) | 0  (0%) |
|  | **Pruritus** | 2 (12%) | 2 (12%) | 0  (0%) | 0  (0%) | 0  (0%) | 0  (0%) | 0  (0%) | 0  (0%) | 0  (0%) | 0  (0%) | 0  (0%) | 0  (0%) | 0  (0%) | 0  (0%) | 0  (0%) | 0  (0%) | 0  (0%) | 0  (0%) |

Supplementary Table S3. Efficacy and Tumor Response of Trametinib

|  | **Normal (Norm)**  **(2 mg)** | **Mild DL1**  **(2 mg)** | **Moderate (Mod) DL1 (1.5 mg)** | **Moderate (Mod) DL+1 (2 mg)** | **Severe (Sev) DL1 (1 mg)** | **Severe (Sev) DL+1 (1.5 mg)** | **Total** |
| --- | --- | --- | --- | --- | --- | --- | --- |
| Number of Patients | 17 | 7 | 4 | 5 | 9 | 2 | 44 |
| Best Response | n (%) | n (%) | n (%) | n (%) | n (%) | n (%) |  |
| **Partial Response** | 2 (15.4) | 0 (0) | 0 (0) | 0 (0) | 0 (0) | 0 (0) | 2 |
| **Stable Disease** | 7 (53.8) | 2 (33.3) | 3 (75.0) | 2 (66.7) | 3 (75.0) | 0 (0) | 17 |
| **Progressive Disease** | 4 (30.8) | 4 (66.7) | 1 (25.0) | 1 (33.3) | 1 (25.0) | 1 (100%) | 12 |
| **Evaluable** | 13 (76%) | 6 (86%) | 4 (100%) | 3 (60%) | 4 (44%) | 1 (50%) | 31 |

Supplementary Table S4. PK evaluable patients and reasons of PK non-evaluability

|  | Norm | Mild | Mod  (1.5mg) | Mod  (2mg) | Sev  (1mg) | Sev  (1.5mg) | Total |
| --- | --- | --- | --- | --- | --- | --- | --- |
| **Number of patients** | 17 | 7 | 4 | 5 | 9 | 2 | 44 |
| **PK evaluable patients** | 10 | 6 | 2 | 1 | 2 | 1 | 22 |
| **Reasons for PK non-evaluability** | | | | | | | |
| 1. **Withdrawal of consent** | 0 | 1 | 0 | 0 | 0 | 0 | 1 |
| 1. **Dose interruption/modification due to treatment related AEs** | 7 | 0 | 0 | 1 | 0 | 0 | 8 |
| 1. **Dose interruption/modification due to treatment unrelated AEs** | 0 | 0 | 2 | 1 | 3 | 0 | 6 |
| 1. **Disease progression** | 0 | 0 | 0 | 1 | 2 | 1 | 4 |
| 1. **Others** | 0 | 0 | 0 | 1*** | 2**^/^*** | 0 | 3 |

** 1 patient had elevation of total bilirubin at baseline (C1D1) of Sev group but rapidly resolved to normal bilirubin (Norm group criteria) through out all the blood test timepoint in DLT period (Cycle 1)

*** 1 patient each from Mod (2mg) and Sev (1mg) had incomplete PK sample collection
