# Supplementary material for: Phase I pharmacokinetic study of single agent trametinib in patients with advanced cancer and hepatic dysfunction
Source: J Exp Clin Cancer Res. 2022 Feb 7;41:51. doi: 10.1186/s13046-021-02236-7 (PMC8819907; doi:10.1186/s13046-021-02236-7)
Supplement: Supplementary file 2 — Additional file 2. [file 13046_2021_2236_MOESM2_ESM.pdf]

Supplementary Figure S1. Progression Free Survival Kaplan Meier Curve

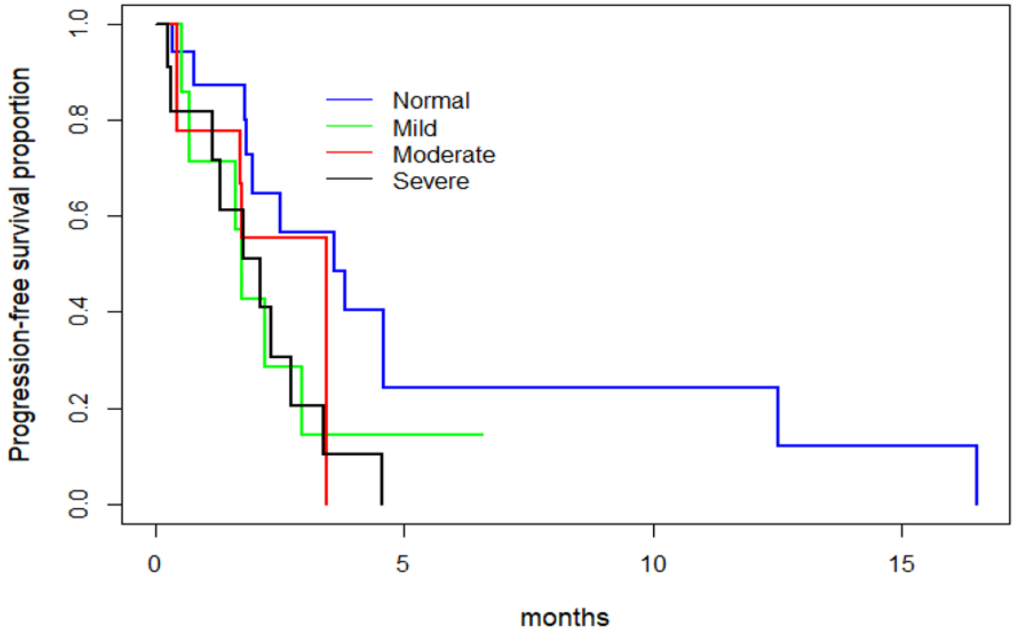

	Events	Median	95% CI	P value
**Norm group**	12	3.62	1.81-4.61	0.12
**Mild group**	6	1.74	0.53-2.96	
**Mod group**	6	3.45	0.46-NA	
**Sev group**	10	2.11	0.33-2.73	
