# Supplementary material for: Phase I pharmacokinetic study of single agent trametinib in patients with advanced cancer and hepatic dysfunction
Source: J Exp Clin Cancer Res. 2022 Feb 7;41:51. doi: 10.1186/s13046-021-02236-7 (PMC8819907; doi:10.1186/s13046-021-02236-7)
Supplement: Supplementary file 3 — Additional file 3. [file 13046_2021_2236_MOESM3_ESM.pdf]

SUMMARY OF CHANGES

For Protocol Amendment #16:

NCI Protocol #: 9591
Local Protocol #: PJC-015

NCI Version Date: June 05, 2020
Protocol Date: June 05, 2020

This amendment is in response to Dr. Johnson's June 5, 2020 request for changes to the trametinib pharmaceutical section.

#	Section	Page(s)	Change
1	Title Page, Header	All	Added the newest protocol version Amendment 16/ Version 19/June 05, 2020

<table>
<tr><th>#</th><th>Section</th><th>Page(s)</th><th>Change</th></tr>
<tr><td>2</td><td>8.1</td><td>53-54</td><td>Protocol section has been updated as per the notification from the company collaborator indicating Trametinib supply will transition from commercially-labeled supply for investigational use to investigationally-labeled supply in the next 30 days

Updated language is listed below:

• How Supplied: Novartis supplies and CTEP, NCI, DCTD distributes trametinib as 0.5 mg and 2 mg (as free base) tablets. Each investigationally-labeled bottle contains 32 tablets.

The tablet core contains mannitol, microcrystalline cellulose, hypromellose, croscarmellose sodium, magnesium stearate (non-animal), colloidal silicon dioxide and sodium lauryl sulfate.

  ◦ 0.5 mg tablets are yellow, modified oval, biconvex and film-coated. Aqueous film coating consists of hypromellose, titanium dioxide, polyethylene glycol, iron oxide yellow.
  ◦ 2 mg tablets are pink, round, biconvex and film-coated. Aqueous film coating consists of hypromellose, titanium dioxide, polyethylene glycol, polysorbate 80, iron oxide red.

• Storage: Store tablets at 2°C -8°C in the original bottle and dispense unopened bottles. Do not open bottles or repackage tablets or remove desiccant. Bottles should be protected from light and moisture.

If a storage temperature excursion is identified, promptly return trametinib to 2°C -8°C and quarantine the supplies. Provide a detailed report of the excursion (including documentation of temperature monitoring and duration of the excursion) to PMBAfterHours@mail.nih.gov for determination of suitability.

• Stability: Stability studies are ongoing. Tablets are only stable for 32 days once bottle has been opened. If multiple bottles are dispensed to a patient in the same visit, please advise the patient to open only one bottle at a time.</td></tr>
</table>

NCI Protocol #: 9591

Local Protocol #: PJC-015

Title: A Phase I Trial of Single Agent Trametinib (GSK1120212) in Advanced Cancer Patients with Hepatic Dysfunction

Corresponding Organization: **LAO-11030** / University Health Network Princess Margaret Cancer Center LAO

***Principal Investigator:** Dr. Lillian Siu
Princess Margaret Cancer Centre
Department of Medical Oncology
610 University Avenue, 5-718
Toronto, Ontario, CANADA
M5G 2M9
Phone: 416-946-2911
Fax: 416-946-4467
lillian.siu@uhn.ca

Participating Organizations:

**LAO-CA043** / City of Hope Comprehensive Cancer Center LAO
**LAO-CT018** / Yale University Cancer Center LAO
**LAO-MA036** / Dana-Farber - Harvard Cancer Center LAO
**LAO-MD017** / JHU Sidney Kimmel Comprehensive Cancer Center LAO
**LAO-MN026** / Mayo Clinic Cancer Center LAO
**LAO-NC010** / Duke University - Duke Cancer Institute LAO
**LAO-NJ066** / Rutgers University - Cancer Institute of New Jersey LAO
**LAO-OH007** / Ohio State University Comprehensive Cancer Center LAO
**LAO-PA015** / University of Pittsburgh Cancer Institute LAO
**LAO-TX035** / University of Texas MD Anderson Cancer Center LAO
**LAO-NCI /** National Cancer Institute LAO

Statistician:
Lisa Wang
Princess Margaret Cancer Centre
610 University Ave
Toronto, ON Canada M5G 2M9
Phone: 416-946-4501 ext 4883
Fax: 416-946-2048
lisawang@uhnres.utoronto.ca

Study Coordinator:
Arti Singh
Princess Margaret Cancer Centre
700 University Ave, 2nd Floor North,
Toronto, ON Canada M5G 1Z5
Phone: 416-946-4501 ext 3842
Fax: 416-946-4607
arti.singh@uhn.ca

Responsible Research Nurse:
Dave Zwir
Princess Margaret Cancer Centre
610 University Ave
Toronto, ON, M5G 2M9
Phone: 416-946-4501 ext 4723
Fax: 416-946-6559
dave.zwir@uhn.ca

Program Manager:
Patrick Marban
Princess Margaret Cancer Centre
700 University Ave
2nd Floor, North, Rm 216
Toronto, ON M5G 1Z5
Phone: 416-946-4616
Fax: 416-946-2390
patrick.marban@uhn.ca

NCI Supplied Agent: Trametinib dimethyl sulfoxide (GSK1120212B, MEKINIST™) (NSC 763093; IND #119752)

IND Sponsor: DCTD, NCI

Protocol Type / Version # / Version Date:
Original / Version 1 / August 13, 2013
Revision 1 / Version 2 / September 10, 2013
Revision 2 / Version 3 / September 23, 2013
Amendment 1 / Version 4 / December 10, 2013
Amendment 2 / Version 5 / April 14, 2014
Amendment 3/ Version 6/ July 28, 2014
Amendment 4/Version 7/ February 12, 2015
Amendment 5/Version 8/June 1, 2015
Amendment 6/Version 9/October 21, 2015
Amendment 7/Version 10/November 13, 2015
Amendment 8/Version 11/April 12, 2016
Amendment 9/Version 12/November 21, 2016
Amendment 10/Version 13/March 10, 2017
Amendment 11/Version 14/July 13, 2017
Amendment 12/Version 15/January 16, 2018
Amendment 13/Version 16/October 22, 2018
Amendment 14/Version 17/February 25, 2019
Amendment 15/Version 18/December 5, 2019
Amendment 16/Version 19/June 05, 2020

SCHEMA

LIVER DYSFUNCTION GROUP

This is a single-arm, dose finding, phase I clinical trial wherein advanced cancer patients with varying degrees of hepatic dysfunction, deemed eligible, will be treated with the MEK inhibitor trametinib. Patients will be stratified into 4 groups or cohorts (A: normal, B: mild dysfunction, C: moderate dysfunction, D: severe dysfunction) according to their hepatic function as outlined in the following schema.

After enrollment, trametinib will be taken orally once a day during a 28-day cycle. Dose escalation will be performed according to well-defined criteria. The patients will be requested to maintain a medication diary, which will be returned to clinic staff at the end of each course. All patients who achieve stable disease (SD) or partial response (PR) after 2 cycles of treatment will continue on trial unless intolerable toxicities are reported. Treatment discontinuation will occur upon progression disease (PD), toxicity or informed consent (IC) withdrawal/physician decision to discontinue.

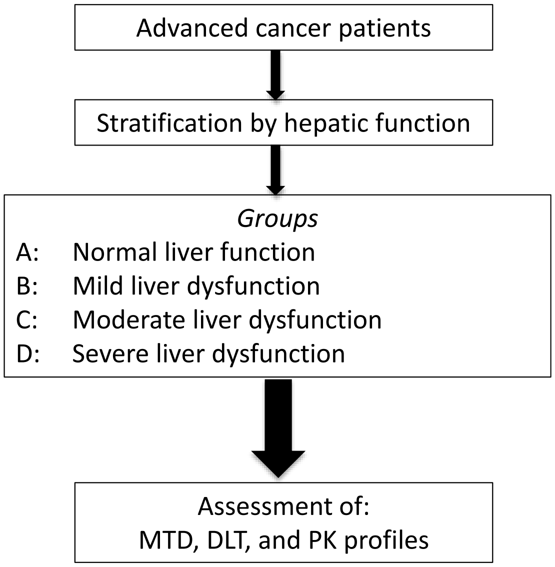

Trametinib will be given daily on a 28-day cycle.

	**Group A**	**Group B**	**Group C**	**Group D**
	**Normal** hepatic function	**Mild** hepatic dysfunction	**Moderate** hepatic dysfunction	**Severe** hepatic dysfunction
**Dose Level**	mg	mg	mg	mg
Level - 2	-	1	0.5	-
Level - 1	-	1.5	1	0.5
Level 1	2	2	1.5	1
Level +1	no escalation	no escalation	2	1.5
Level +2	no escalation	no escalation	no escalation	2

TABLE OF CONTENTS

SCHEMA 3

1. OBJECTIVES 6
   1.1 Primary Objectives 6
   1.2 Secondary Objectives 6

2. BACKGROUND 6
   2.1 Hepatic dysfunction population 6
   2.2 Trametinib Dimethyl Sulfoxide (GSK1120212B, MEKINIST™) 7
   2.3 Trametinib Dimethyl Sulfoxide (GSK1120212B, MEKINIST™) Patients' Population Selection 15
   2.4 Rationale 17

3. PATIENT SELECTION 18
   3.1 Eligibility Criteria 18
   3.2 Exclusion Criteria 20
   3.3 Inclusion of Women and Minorities 22

4. REGISTRATION PROCEDURES 23
   4.1 Investigator and Research Associate Registration with CTEP 23
   4.2 Site Registration 24
   4.3 Patient Registration 26
   4.4 General Guidelines 27

5. TREATMENT PLAN 27
   5.1 Stratification by Hepatic Function 27
   5.2 Trametinib Dimethyl Sulfoxide (GSK1120212B, MEKINIST™) Administration 28
   5.3 Definition of Dose-Limiting Toxicity 29
   5.4 General Concomitant Medication and Supportive Care Guidelines 32
   5.5 Duration of Therapy 32
   5.6 Duration of Follow Up 32
   5.7 Criteria for Removal from Study 32

6. DOSING DELAYS/DOSE MODIFICATIONS 33
   6.1 Trametinib Dimethyl Sulfoxide (GSK1120212B, MEKINIST™) Dose Modifications 33

7. ADVERSE EVENTS: LIST AND REPORTING REQUIREMENTS 45
   7.1 Comprehensive Adverse Events and Potential Risks list (CAEPR) 45
   7.2 Adverse Event Characteristics 49
   7.3 Expedited Adverse Event Reporting 49
   7.4 Routine Adverse Event Reporting 51
   7.5 Secondary Malignancy 51
   7.6 Second Malignancy 51

8. PHARMACEUTICAL INFORMATION....51
8.1 Trametinib dimethyl sulfoxide (GSK1120212B, MEKINIST™) (NSC 763093)....51

9. BIOMARKER, CORRELATIVE, AND SPECIAL STUDIES....54
9.1 Pharmacokinetic Studies....54
9.2 Biomarker Studies....57

10. STUDY CALENDAR....58

11. MEASUREMENT OF EFFECT....63
11.1 Antitumor Effect – Solid Tumors....63

12. STUDY OVERSIGHT AND DATA REPORTING / REGULATORY REQUIREMENTS....69
12.1 Study Oversight....69
12.2 Data Reporting....69
12.3 Collaborative Agreements Language....71

13. STATISTICAL CONSIDERATIONS....73
13.1 Study Design/Endpoints....73
13.2 Sample Size/Accrual Rate....74
13.3 Stratification Factors....75
13.4 Analysis of Secondary Endpoints....75

REFERENCES....77

APPENDIX A Performance Status Criteria....80

APPENDIX B CHILD-PUGH Classification (CPC) of Liver Dysfunction....81

APPENDIX C Data Management Guidelines....82

APPENDIX D PK Sampling Guidelines....84

APPENDIX E Patient Medication Diary....86

1. OBJECTIVES

1.1 Primary Objectives

- To provide appropriate dosing recommendations for patients with varying degree of hepatic dysfunction receiving trametinib (mild, moderate and severe).
- To establish the maximum tolerated dose (MTD) and dose-limiting toxicity (DLT) of trametinib in advanced cancer patients with varying degrees of hepatic dysfunction
- To characterize the PK profile of trametinib in advanced cancer patients with varying degrees of hepatic dysfunction.

1.2 Secondary Objectives

- To document the non-DLTs associated with the administration of trametinib in patients with varying degrees of hepatic dysfunction.
- To document any antitumor activity associated with trametinib treatment of patients enrolled on this study.
- To explore and characterize predictive biomarkers for individual cancer patients utilizing genomic sequencing technologies.

2. BACKGROUND

2.1 Hepatic dysfunction population

Advanced cancer patients frequently present with different degrees of hepatic dysfunction, which usually prevent them from receiving standard treatments or participate in clinical trials. Hepatic dysfunction is either a result of metastatic disease or pre-existing medical conditions that often require dose adjustments to avoid accumulation and potential toxicity. In principle, every human tissue has the ability to metabolize drugs; however a safe medication use is mostly dependent on renal and liver functions[1,2]. Liver failure usually increases in incidence with age and is often a challenging condition for medication therapy prescription, when indicated. Liver dysfunction may decrease drug or drug metabolite excretion in phase 1 or phase 2 drug metabolism reactions, which comprise of oxidation, reduction, hydrolysis, cyclization/decyclization, addition of oxygen or removal of hydrogen and methylation, sulphation, acetylation, glucuronidation, glutathione conjugation, glycine conjugation respectively. In the past, the US Food and Drug Administration (FDA) has approved many drugs with little or no PK/PD information in patients with hepatic dysfunction. Information on organ dysfunction metabolisms for cancer agents has traditionally been performed post-marketing[3]. Nowadays, a critical challenge in this patient population is the use of the new and potentially promising targeted agents.

Liver dysfunction can alter both PK and PD, and can cause drug accumulation or, in a minority of cases, prevent active metabolite formation[4]. Patients with hepatic dysfunction

are generally unstable and clinical conditions may deteriorate very rapidly leading to acute liver failure[4]. Similar to renal dysfunction, liver disease may alter kidney function, leading to accumulation of drugs and /or metabolites regardless of route of elimination[4,5]. In the attempt of establishing reliable laboratory hepatic function measurements, physicians have utilized the Child-Pugh Liver Dysfunction Classification[6].

Although some agents' pharmacokinetics are unaltered in hepatic dysfunction, patients with liver disease may still experience increased sensitivity to specific drugs, therefore characterizing the safety profile of anticancer agents in dedicated clinical studies is the correct way to develop appropriate guidelines for the use of these agents in patients with hepatic dysfunction.

2.2 Trametinib Dimethyl Sulfoxide (GSK1120212B, MEKINIST™)

2.2.1 Trametinib Dimethyl Sulfoxide (GSK1120212B, MEKINIST™)

The RAF-MEK-ERK pathway plays a critical role in multiple cellular functions. Activation of the pathway can result from activation/mutations of the upstream receptor tyrosine kinases (RTKs) and RAS, or upregulation/mutations in RAF and MEK. Upon activation, RAF acts as the MAPK kinase kinase and activates MAPKK (MEK1/2), which in turn catalyze activation of the effectors ERK1/ERK2. Once activated, ERK1/2 translocates into the nucleus and phosphorylates a number of effector proteins and transcriptional factors that regulate cell proliferation, motility, differentiation, and survival.

Trametinib is one of the several MEK inhibitors in clinical development. On May 29, 2013, the U.S. Food and Drug Administration (FDA) approved trametinib for the treatment of patients with unresectable or metastatic melanoma with $BRAF^{V600E}$ or $BRAF^{V600K}$ mutations as detected by an FDA-approved test[7]. On January 10, 2014, the Food and Drug Administration granted accelerated approval to trametinib and dabrafenib for use in combination to treat patients with unresectable or metastatic melanoma with a BRAF V600E or V600K mutation as detected by an FDA-approved test[8].

Experience to date indicates that MEK is a valid target. In a phase III trial comparing trametinib with dacarbazine or paclitaxel in patients with BRAF V600E or V600K mutant metastatic melanoma, trametinib demonstrated a significantly better response rate, progression-free survival, and overall survival[9]. However, single agent activities are limited. Extensive research is underway to identify the patient selection markers and develop rational combination strategies. Preclinical studies have provided strong rationale and proof of principle for combination of MEK inhibitors with RTK inhibitors (EGFR or IGF-1R)[10,11], PI3K/AKT inhibitors[12,13], and mTOR inhibitors. On the other hand, the optimal dose/schedule and patient selection criteria for combination regimens have not been defined. Phase 1 results for a number of combinations have been reported, including AZD6244 + MK2206 (MEK + AKT inhibitors)[14] and GDC-0973 + GDC-094 (MEK + PI3K inhibitors)[15].
The most up-to-date preclinical and clinical study information for trametinib can be found in the GSK1120212 (trametinib)[16].

2.2.1.1 Mechanisms of Action and Preclinical Data with Trametinib

Trametinib is a dimethyl sulfoxide (DMSO) solvate compound (ratio 1:1) with potent, allosteric and ATP non-competitive inhibition of MEK1/2 (IC_{50} of 0.7 and 0.9 nM against MEK1 and MEK2, respectively)[17]. Trametinib inhibited MEK1/2 kinase activity and prevented RAF-dependent MEK phosphorylation (S217 for MEK1), producing prolonged pERK1/2 inhibition. Trametinib showed better potency against unphosphorylated MEK1/2 (u-MEK1/2) when compared with preactivated diphosphorylated MEK (pp-MEK), suggesting that u-MEK affords a higher affinity binding site for trametinib than does pp-MEK.

The specificity of trametinib was confirmed against a panel of 183 kinases, including MEK5 (the closet kinase homolog to MEK1/2), CRAF, BRAF, ERK1, and ERK2[18]. Trametinib demonstrated equal potency against activated MEK1- and MEK2-mediated phosphorylation of ERK (sequence identity of 85% across the whole protein and 100% in the active site for humans). Trametinib demonstrated preferential inhibition of RAF-mediated MEK1 activation (IC_{50} = 0.60 nM) over pMEK1 kinase activity (IC_{50} = 13 nM)[19].

BRAF-mutant Colo205, A375P F11s, and HT-29 human tumor xenograft mouse models showed the most significant mean tumor growth inhibition (TGI) (80% to 87%) at 3.0 mg/kg trametinib, with multiple complete and partial tumor regressions. In the Colo205 model, tumor regression was observed even at a dose of 0.3 mg/kg[18]. Two KRAS-mutant xenograft models, HCT-116 and A549, also showed significant TGI (83% and 75%) but without significant tumor regressions[17]. As predicted by cell proliferation assays, tumor xenograft lines with wild-type (wt) RAF/RAS (PC3, BxPC3, and BT474) were much less sensitive, showing only modest TGI (44-46%) with no tumor regressions.

Pharmacodynamic studies were performed in mice treated with trametinib for 14 days[17]. In the A375P F11s xenograft model, the first dose of trametinib (3 mg/kg) significantly reduced pERK for more than 8 hours on Day 1. pERK inhibition was more sustained (over 24 hours) after the Day 7 dose, probably due to an increase in the steady-state levels of trametinib after repeated doses. The average C_{max} in blood was 1,410 nM on Day 7, with an estimated half-life ($t_{1/2}$) of 33 hours. In addition, immunohistochemistry (IHC) also confirmed inhibition of cell proliferation (reduced Ki67) and G1 cell cycle arrest (elevated p27Kip1/CDKN1B) following 4 days of treatment.

2.2.1.2 Clinical Pharmacokinetics (PK) and Activity of Trametinib

FTIH Phase 1 Trial of Trametinib Monotherapy (MEK111054)
There are 3 parts in this study. Part 1: The dose-escalation portion involves administration of trametinib (repeat doses of 0.125 mg to 4.0 mg) to patients with solid tumors or lymphoma in one of three schedules - (1) QD for 21 days followed by 7 days without drug, (2) loading dose on Day 1 or Day 1-2, followed by QD with the

designated dose, or (3) QD dosing without a drug holiday. Part 2: cohort expansion at the recommended phase 2 dose (RP2D) for pancreatic cancer, melanoma, non-small cell lung cancer (NSCLC), colorectal cancer (CRC), or any BRAF mutation-positive cancer. Part 3: expansion to characterize the biologically active range of trametinib via analysis of pharmacodynamic biomarkers (biopsies or FDG-PET).

The MTD of trametinib was established as 3 mg QD, but the recommended phase 2 dose (RP2D) was chosen at 2 mg QD based on tolerability of repeated cycles[20].

PK and metabolism of trametinib:
PK measurements were conducted under fasting conditions. After a single dose (Day 1), AUC_{0-24} and C_{max} values were dose-proportional up to 6 mg, lower than dose proportional following 8 mg, and greater than dose proportional following the 10 mg dose. Median T_{max} was 1.5 hours.

After repeat doses (Day 15), trametinib accumulated with a mean accumulation ratio of 6.6 at the RP2D of 2 mg QD. Between-subject variability in exposure ranged from 27-50% for C_{max} and 20-41% for AUC_{0-24} across all dosing regimens. The effective $t_{1/2}$ was approximately 4.5 days, and steady state was reached by approximately Day 15. Trametinib had a small peak: trough ratio of ~2[(20)]. At 2 mg QD on Day 15, mean AUC_{0-24} was 376 ng•h/mL and C_{max} 23 ng/mL, and the mean trough concentrations ranged from 10.0 to18.9 ng/mL. The long half-life and small peak: trough ratio of trametinib allowed constant target inhibition within a narrow range of exposure.

Drug-drug interactions:
Trametinib is metabolized predominantly via deacetylation (non-cytochrome P450 [CYP450]-mediated) with secondary oxidation or in combination with glucuronidation biotransformation pathways[19]. The deacetylation is likely mediated by hydrolytic esterases, such as carboxylesterases, or amidases. Based on *in vitro* studies, trametinib is not an inhibitor of CYP1A2, CYP2A6, CYP2B6, CYP2D6, and CYP3A4. Trametinib has an overall low potential for drug-drug interactions.

Pharmacodynamic effect and biomarkers:
The relationship between dose and tumor biomarkers such as pERK, Ki67, and p27, were evaluated in patients with BRAF or NRAS mutation-positive metastatic melanoma[19]. In general, increasing exposures and/or doses provided greater pharmacodynamic effects. The median change observed at a dose of 2 mg QD was 62% inhibition of pERK, 83% inhibition of Ki67, and a 175% increase in p27.

Antitumor Activity in the FTIH phase 1 trial:
In the FTIH phase 1 trial, 14 patients with BRAF-mutant melanoma received trametinib at 2 mg QD. The overall objective response rate (ORR) was 43% (6/14), including 2 complete responses (CRs)[19]. In 9 patients with BRAF wt melanoma, 2 patients achieved a partial response (PR), and 3 stable disease (SD)[20]. In 26 evaluable pancreatic cancer patients, there were 2 PRs (1 PR was KRAS mutation-positive) and 11 SD (2 achieved ≥20% tumor reduction)[21]. Among the 27 CRC patients (without

selection of RAS or RAF mutations), 8 SD were observed.

Antitumor Activity in Melanoma

Phase 3 trial of trametinib vs. chemotherapy in advanced V600 mutant melanoma:
In a phase 3 trial, patients with unresectable stage IIIC or IV cutaneous melanoma with a BRAF V600E or V600K mutation were randomized (2:1) to trametinib (2 mg, PO, QD) or chemotherapy (dacarbazine or paclitaxel)[9,22]. There were 322 patients in the intention-to-treat (ITT) population, of whom 273 (85%) were in the primary efficacy population (patients with $BRAF^{V600E}$-positive cancer who did not have brain metastases at baseline). Of the patients, 214 were randomized to receive trametinib, and 108 were randomized to receive chemotherapy. Investigator-assessed efficacy data are summarized in Table 1.

Table 1: Summary of the investigator-assessed efficacy data of the Phase 3 trial of trametinib versus chemotherapy in advanced V600 mutant melanoma

	**Trametinib (n=214)**	**Chemotherapy (DTIC) (n=108)**
**PFS**<br>Median, months (95% CI)	4.8 (4.3, 4.9)	1.5 (1.4, 2.7)
HR (95% CI)<br>*P* value (log-rank test)	0.47 (0.34, 0.65)<br>*P*<0.0001	
**Confirmed Tumor Responses**<br>Objective Response Rate (95% CI)<br>CR, n (%)<br>PR, n (%)	 22% (17, 28)<br>4 (2%)<br>43 (20%)	 8% (4, 15)<br>0<br>9 (8%)
Duration of response<br>Median, months (95% CI)	5.5 (4.1, 5.9)	NR (3.5, NR)
CI = confidence interval; CR = complete response; HR = hazard ratio; NR = not reached; PFS = progression-free survival; PR = partial response		

The 6-month OS rate was 81% in the trametinib group and 67% in the chemotherapy group. Mature data on OS are pending.

Experience with Trametinib in Metastatic Melanoma Following BRAF Inhibitor Therapy

The clinical activity of single-agent trametinib was evaluated in a single-arm, multicenter, international trial in 40 patients with BRAF V600E or V600K mutation-positive, unresectable, or metastatic melanoma who had received prior treatment with a BRAF inhibitor. All patients received trametinib at a dose of 2 mg PO QD until disease progression or unacceptable toxicity. None of the patients achieved a confirmed PR or CR.

Antitumor Activity of Trametinib in Cancer Other Than Melanoma

In a phase 1/2 monotherapy study, acute myeloid leukemia (AML) or myelodysplastic syndrome (MDS) patients were given trametinib at dose levels from 1-2 mg QD. Drug-related AEs in 45 patients were similar to that observed in patients with solid tumors, and 2 mg PO QD was selected for further investigation in this patient population. Twelve patients (23%) withdrew due to an AE, including cardiac failure (2) and infection (2). Efficacy was reported in 39 patients[23]. The best response in 13 patients with KRAS or NRAS mutations included 3 CRs (23%), 7 SD (54%), and 1 PD (progressive disease) (5%). In 26 patients with wild-type RAS or an unknown mutation, there were 2 PRs (8%).

In a multicenter phase 2 study, NSCLC patients with KRAS mutant tumors were randomized 2:1 to receive trametinib (2 mg QD) or docetaxel (75 mg/m^2 IV every 3 weeks)[24]. A total of 134 pts were randomized to trametinib (89) or docetaxel (45); 129 patients had KRAS-mutant NSCLC. The hazard ratio for PFS was 1.14 (95% CI, 0.75-1.75; P=0.5197) with a median PFS of 11.7 versus 11.4 weeks for trametinib versus docetaxel. The overall response rate (ORR) was 12% for trametinib and 12% for docetaxel.

In a double-blind, phase 2 study evaluating the combination of gemcitabine with trametinib, untreated pancreatic cancer patients were randomized to receive gemcitabine (1000 mg/m^2 weekly ×7 for 8 weeks, then weekly ×3 every 4 weeks) plus either trametinib 2 mg or placebo QD[25]. Median OS was 8.4 months with trametinib compared to 6.7 months with placebo. Median PFS was 16 weeks versus 15 weeks, and ORRs and median duration of responses were 22% and 23.9 weeks and 18% and 16.1 weeks on trametinib and placebo; the median OS and ORR in the subgroup of patients with KRAS mutations (143/160) was similar to OS and ORR for all randomized patients.

2.2.1.3 Trametinib Safety Profile

A **Comprehensive Adverse Events and Potential Risks (CAEPR)** list using NCI Common Terminology Criteria for Adverse Events (CTCAE) terms is included in Section 7.1 of the protocol.

Based on available AE data from clinical studies involving trametinib to date, the most common toxicities are rash and diarrhea. Rash and diarrhea are common, class-effect toxicities for MEK inhibitors. In addition, visual impairment and left ventricular ejection fraction (LVEF) reduction, although observed at lower frequencies, are also considered class-effect toxicities as they have been observed with trametinib as well as other MEK inhibitors.

AEs of special interest:
Rash, diarrhea, visual disorders, hepatic disorders, cardiac-related AEs, and pneumonitis are considered AEs of special interest because they are either known class effects (*i.e.*,

have been observed with other MEK inhibitors) or are potentially life-threatening[26]. The following sections provide integrated summaries for these AEs across different clinical trials, with emphasis on trials using trametinib as monotherapy, especially at the RP2D of 2 mg.

Refer to dose modification guidelines for the toxicities for which they are addressed in Section 6.

Rash: Rash was a common AE observed across different dose levels and in different combinations[16]. At the 2 mg dose, rash was seen in 27% to 78% of patients in different trials. Of the ~370 subjects with rash AEs at the 2 mg monotherapy dose (including crossover subjects) in five studies, the majority of rash AEs were grades 1 or 2 (24% to 73%); 0% to 9% of patients experienced grade 3 rash AEs, and four patients had a grade 4 rash AE.

In a randomized phase 3 trial of trametinib vs. chemotherapy, the overall incidence of skin toxicity (including rash, dermatitis, acneiform rash, palmar-plantar erythrodysesthesia syndrome, and erythema) was 87% in patients treated with trametinib and 13% in chemotherapy-treated patients. Severe skin toxicity occurred in 12% of patients on the trametinib arm, most commonly for secondary infections of the skin. The median time to onset of skin toxicity was 15 days (range: 1 to 221 days), and median time to resolution was 48 days (range: 1 to 282 days). Dose reduction was required in 12% for skin toxicities, and permanent discontinuation of trametinib was required in 1% of patients.

Diarrhea: At the 2 mg monotherapy dose, 33% to 58% of patients in five trials had diarrhea[16]. Of ~320 subjects (including crossover subjects) with diarrhea at this dose, the majority of diarrhea AEs were grade 1 or 2 in severity (33% to 56% of all study patients); 17 patients had grade 3 diarrhea, and none had grade 4 diarrhea.

Visual disorders: At the 2 mg monotherapy dose, 4% to 21% of the patients in five trials experienced visual disorders[16]. Of the 85 total subjects (including crossover subjects) experiencing visual disorders at this dose level, the majority of visual disorders were grades 1 or 2 (4% to 20% of all study patients); six patients experienced grade 3 visual disorders, and one patient experienced a grade 4 visual disorder.

- *Retinal Pigment Epithelial Detachment (RPED):* Also known as chorioretinopathy, RPED is a visual impairment due to fluid accumulation under the retina and causes blurry vision. There were five cases of RPED, previously termed central serous retinopathy, reported from the integrated trametinib safety population consisting of subjects treated with trametinib 2 mg once daily from five studies[16]. As of 23 June 2013, 14 cases of RPED were reported across the entire trametinib program amongst subjects treated with trametinib either as monotherapy or in combination with other anti-cancer agents (including cases from a MEK/BRAF combination study).

- *Retinal vein occlusion (RVO):* As of 23 June 2013, a total of four cases of RVO

were reported across the entire trametinib program (including one case from a MEK/BRAF combination study)[16]. All cases of RVO occurred in one eye only. Study drug was stopped at time of diagnosis in all cases. There was a decrease of visual acuity in two subjects with central RVO (CRVO) while the other two subjects had no meaningful decrease of visual acuity. In the two subjects with CRVO, local treatment with intravitreal injections of anti-VEGF antibodies was initiated within 2 weeks after RVO diagnosis, and visual acuity improved in one subject and restored to baseline conditions in another subject, at the time of the data cutoff. Three of these four cases were considered related to study treatment by the investigators.

Hepatic disorders: Abnormalities of liver enzymes and bilirubin have been observed with administration of trametinib. However, assessment of these cases was often confounded by co-morbid conditions (such as biliary obstruction), concomitant use of other potentially hepatotoxic drugs, and liver metastases. At the 2 mg monotherapy dose, 8% to 34% of patients in five trials had LFT abnormalities. Of the 96 total patients (including crossovers) with LFT changes, the majority were grade 1 or 2 in severity (4% to 20% of all study patients); 26 had grade 3 events, and 6 patients had grade 4 events.

Cardiac-related AEs: At the 2 mg monotherapy dose, 3% to 21% of the subjects in six studies had cardiac-related AEs[16]. Of the 65 total subjects (including crossover subjects) experiencing cardiac-related AEs at the 2.0 mg monotherapy dose in five of the studies, the majority of cardiac-related AEs were grades 1 or 2 in severity (0% to 16% of all study subjects); 18 subjects had grade 3 cardiac-related AEs, and no subjects had Grade 4 cardiac-related AEs in any study. No subject in one study, which evaluated the effect of repeat oral dosing of trametinib 2 mg QD on cardiac repolarization in subjects with solid tumors, had cardiac-related AEs. One study subject receiving trametinib 2 mg QD had grade 5 (fatal) acute cardiac failure, with evidence of massive tumor invasion of the heart; this AE was considered not drug-related by the investigator.

In the phase 3 trial of trametinib vs. chemotherapy in patients with melanoma (MEK114267), cardiomyopathy (defined as cardiac failure, left ventricular dysfunction, or decreased LVEF] occurred in 7% (14/211) of patients treated with trametinib, and in no patients in the chemotherapy arm. Cardiomyopathy was identified within the first month of treatment in five of these 14 patients; median onset of cardiomyopathy was 63 days (range: 16 to 156 days). Cardiomyopathy resolved in 10 of these 14 (71%) patients. Cardiac monitoring should be included in trametinib protocols, to include LVEF assessment by echocardiogram or MUGA scan at baseline, one month after initiation of trametinib and then at 2- to 3-month intervals while on treatment. Refer to dose modification guidelines for cardiac AEs in the event of LVEF decline or symptomatic cardiac AEs.

Pneumonitis: At the 2 mg monotherapy dose, 0% to 4% of the subjects in five studies had pneumonitis[16]. Of the nine total subjects (including crossovers) experiencing pneumonitis AEs at this dose, three subjects had grade 1 or 2 pneumonitis and six subjects had grade 3 pneumonitis.

Embryofetal toxicity: Based on its mechanism of action, trametinib can cause fetal harm when administered to a pregnant woman. Trametinib was embryotoxic and abortifacient

in rabbits at doses greater than or equal to those resulting in exposures approximately 0.3 times the human exposure at the recommended clinical dose. If this drug is used during pregnancy, or if the patient becomes pregnant while taking this drug, the patient should be apprised of the potential hazard to a fetus.

Incidence of common AEs reported from a phase III trial of trametinib vs. chemotherapy in patients with advanced melanoma:

Patients with abnormal LVEF, history of acute coronary syndrome within 6 months, or current evidence of Class II or greater congestive heart failure (New York Heart Association) were excluded from this trial. Selected adverse reactions (AR) occurring in patients receiving trametinib as compared to patients in the chemotherapy arm are listed as below:

Table 2: Selected adverse reactions (ARs) occurring in ≥10% of patients receiving trametinib AND at a higher incidence than in the chemotherapy arm (high in the trametinib arm compared with chemotherapy by ≥5% in overall incidence or by ≥2% grade 3 or 4 AEs)

	**Trametinib (n=211)**		**Chemotherapy (n=99)**	
**Adverse Reactions**	**All Grades**	**Grades 3 and 4**	**All Grades**	**Grades 3 and 4**
**Skin and subcutaneous tissue disorders**				
Rash	57	8	10	0
Dermatitis acneiform	19	<1	1	0
Dry skin	11	0	0	0
Pruritis	10	2	1	0
Paronychia	10	0	1	0
**Gastrointestinal disorders**				
Diarrhea	43	0	16	2
Stomatitis	15	2	2	0
Abdominal pain	13	1	5	1
**Vascular disorders**				
Lymphedema	32	1	4	0
Hypertension	15	12	7	3
Hemorrhage	13	<1	0	0

Table 3: Percent-patient incidence of laboratory abnormalities occurring at a higher incidence in patients treated with trametinib versus chemotherapy (between-arm difference of ≥5% [all grades] or ≥2% [grades 3 or 4])

	**Trametinib (n=211)**		**Chemotherapy (n=99)**	
**Preferred term**	**All Grades**	**Grades 3 and 4**	**All Grades**	**Grades 3 and 4**
Increased aspartate aminotransferase (AST)	60	2	16	1
Increased alanine aminotransferase (ALT)	39	3	20	3
Hypoalbuminemia	42	2	23	1
Anemia	38	2	26	3
Increased alkaline phosphatase	24	2	18	3

Other clinically important adverse reactions observed in ≤10% of patients (n=329) treated with trametinib were: nervous system disorders (dizziness, dysgeusia), ocular disorders (blurred vision, dry eye), infections and infestations (folliculitis, rash pustular, cellulitis), cardiac disorders (bradycardia), gastrointestinal disorders (xerostomia), and musculoskeletal and connective tissue disorders (rhabdomyolysis).

2.2.1.4 Clinical Experience with the Combination of Trametinib + Dabrafenib

Please refer to Dabrafenib protocol template for information on the combination of dabrafenib and trametinib.

2.2.1.5 Clinical Experience with the Combination of Trametinib + GSK2141795 (AKT inhibitor) (TAC113886)

Twenty-three patients with advanced solid tumors received the combination using a zone-based escalation procedure enabling evaluation of multiple combination doses in parallel cohorts[27]. While the RP2D for single agent for single agent trametinib and GSK2141795 are 2 mg/d and 75 mg/d, dose reductions were required for the combination. DLTs include grade 2 AST and ALT elevation, and grade 3 chest pain with sustained ventricular tachycardia; all DLTs were reversible with drug interruption. The most common AEs (≥10%) included nausea (26%), AST elevation (22%; grade 3/4, 9%), fatigue (22%) and rash (22%). Three MTDs were defined for variable dose ratios: 2 mg trametinib + 25 mg GSK2141795; 0.5 mg trametinib + 75 mg GSK2141795; and 1.5 mg trametinib + 50 mg GSK2141795. Three of 13 evaluable patients (unselected) had tumor shrinkage of 8% (ovarian), 16% (endometrial), and 17% (ovarian) after 8 weeks on study. The dose regime of 1.5 mg trametinib + 50 mg GSK2141795 will be considered for further development. Additional trials to explore alternate schedules (*e.g.*, intermittent) and pharmacodynamic markers are ongoing.

2.3 Trametinib Dimethyl Sulfoxide (GSK1120212B, MEKINIST™) Patients' Population Selection

Several preclinical and clinical studies have shown a more pronounced activity of the MEK inhibitor trametinib in tumors harboring BRAF-activating mutations, in particular

cutaneous melanoma[17,28-31]. In this clinical setting, the MEK114267 (METRIC) study has showed significant improvement in overall survival (OS) and progression-free survival (PFS) of trametinib versus standard chemotherapy (dacarbazine/paclitaxel)[9,32]. Despite striking results in the melanoma setting, emerging data have recently raised the question whether or not selecting patients, based on mutational status, for trametinib is the most appropriate approach in all cancer types. The rationale for tumor type selection for this trial is justified as follows:

- **Patients with advanced solid tumors (besides melanoma, NSCLC, colorectal cancer, and pancreatic cancers):** With the exception of melanoma, NSCLC and pancreatic cancers, the clinical evaluation of trametinib has not been performed in different solid tumor types specifically enriched for patients with RAS or RAF mutations. Based on published data of another MEK inhibitor, selumetinib, in patients with recurrent low-grade serous carcinoma of the ovary or peritoneum, and in patients with metastatic biliary tract cancer, genotypic selection did not correlate with objective response[33,34].
    - In an open-label, single-arm, phase II study of selumetinib in recurrent low-grade serous carcinoma of the ovary or peritoneum, an overall response rate of 15% were observed in 8/52 patients. Of the 34 patients (65%) who had sufficient DNA for BRAF and KRAS genotyping, 2 (6%) of 34 patients had BRAF mutations, 14 (35%) of 34 patients had KRAS mutations. The differences in the percentage of patients with an objective response for any mutation were not statistically significant.
    - In the open-label, single-arm, phase II study of selumetinib in patients with metastatic biliary tract cancers, an overall response rate of 12% were observed in 3/25 response evaluable patients. No BRAF mutations were found in this study population. Two patients had KRAS mutations and achieved only short-lived stable disease on study.

  Based on these data of selumetinib, there is no justification at this present time to restrict study entry based on genotype in the vast majority of advanced solid tumors. The cases of melanoma, NSCLC and pancreatic cancers are to be handled separately based on the published results with trametinib in these specific tumor types (below).
- **Patients with metastatic melanoma who have been exposed to BRAF inhibitors**: A recent phase II clinical trial of trametinib in BRAF mutant metastatic melanoma previously treated with or without BRAF inhibitors, has shown minimal clinical activity of trametinib in patients pretreated with other BRAF inhibitors, as compared to BRAF inhibitors-naïve patients, suggesting a possible mechanism of resistance[35]. As such, these patients will be excluded from this current protocol.
- **Patients with KRAS mutant NSCLC**: Two multicenter phase I/Ib studies of trametinib in combination with either pemetrexed or docetaxel in KRAS mutant and wildtype advanced NSCLC patients resulted in acceptable tolerability and clinical activity regardless of the patients' mutational status[36,37]. Additionally, a randomized, multicenter, phase II study of trametinib compared with docetaxel in KRAS-mutant NSCLC patients has shown no improvement in PFS in this patient population as compared with docetaxel[24]. In the latter study, 134 NSCLC patients previously treated with platinum-based chemotherapy were randomized 2:1 to

trametinib or docetaxel, and 129 of these patients had KRAS mutations. Among KRAS mutant patients, the median PFS and objective response rate for trametinib were 11.7 weeks and 12%; versus 11.4 weeks and 12% for docetaxel. While this study did not demonstrate an improvement in PFS with trametinib compared to docetaxel, single-agent trametinib did shown antitumor activity in this patient population, and thus may constitute a reasonable option in NSCLC patients with liver dysfunction whereby chemotherapy options may be limited.

- **Patients with colorectal cancer:** In the phase I study of trametinib in patients with advanced cancers[30], no objective responses were observed in 28 patients with colorectal cancer. Of 13 patients with KRAS mutations, 1 had minor response and received treatment for 31 weeks, three others had stable disease. Due to the lack of objective response seen in this patient population, colorectal cancer patients are excluded from the current protocol. No objective response was observed among KRAS wildtype patients in this cohort.
- **Patients with pancreatic cancer**: A randomized, double-blind, placebo-controlled study of trametinib in combination with gemcitabine in metastatic pancreatic cancer patients did not show improvement in overall survival, PFS or response rate in the combination arm as compared to single agent gemcitabine[38]. Based on these negative results, pancreatic cancer patients are excluded from the current protocol.

NOTE: Overall trametinib has shown extremely limited activity in these tumors, however, objective responses had been rarely observed. There are currently no biomarkers to predict if a given patient will or will not benefit. For patients with moderate/severe organ dysfunction, enrollment in this trial may be considered reasonable if they are adequately informed that their chance of benefit is very low.

2.4 Rationale

The effect of trametinib in subjects with a variety of refractory cancers has been evaluated in several clinical trials, administered as both single agent or in combination[17,29-31,35,39,40]. Trametinib pharmacokinetics was determined after single and repeat-dose oral administration of study medication tablets in subjects with solid tumors. Results showed that trametinib is absorbed rapidly with median Tmax generally occurring 1.5 hours after single oral administration under fasting conditions. The absolute oral bioavailability of a single trametinib 2 mg tablet is moderate to high (72%) relative to a co-administered IV microdose. Single-dose administration of trametinib with a high-fat, high-calorie meal resulted in a 70% decrease in maximum observed concentration (Cmax) and a 10% decrease in area under the concentration-time curve from time zero (pre-dose) extrapolated to infinity (AUC(0-∞)) compared to fasted conditions. Following repeat-dosing the mean area under the curve (AUC0-τ) and maximum concentrations (Cmax) increased in an approximately dose proportional manner. Trametinib dose accumulation has been seen with repeat dosing with a mean accumulation ratio at the RP2D of 2 mg once daily of 5.97 and a terminal half-life of 5.3 days, determined after single dose administration. Steady state appears to be achieved by Day 15, with little difference in pre-dose (trough) concentration at the end of the dosing interval (Cτ), Cmax and area under the concentration-time curve from time zero (pre-dose) to 24 hours (AUC(0-24)) between Days 15 and 21. Additionally, trametinib is a low extraction ratio drug based on plasma IV clearance of 3.21 L/hour, which represents approximately 1% of liver blood flow. Trametinib has a high

volume of distribution (Vd) of 1060 L determined following an IV microdose. Furthermore, data from previous studies have shown that fecal excretion is the major route of elimination following trametinib oral dose. This route accounts for >80% of excreted radioactivity recovered (or 39.2 and 35.0% of the radioactive dose in 2 subjects) while urinary excretion accounts for <19% of excreted radioactivity recovered (<10% of the radioactive dose). Given that trametinib is generally deacetylated via hydrolytic esterases, therefore its pharmacokinetics is unlikely to be affected by other agents through metabolic interactions. Based *on in vitro* and *in vivo data* trametinib is unlikely to significantly affect the pharmacokinetics of other products via interactions with CYP enzymes or transporters, and that the PK of trametinib is unlikely to be affected by other drugs.

In the population PK analysis of trametinib, 64 (13.0%) subjects were categorized as having mild hepatic impairment (NCI classification). Exposure to trametinib was not significantly different in these subjects (2% difference) relative to subjects with normal hepatic function.

Currently, no clinical data on trametinib are available in patients with hepatic dysfunction. Data have shown that hepatic metabolism is likely to be the primary route of elimination and it can be anticipated that trametinib concentrations will increase in patients with hepatic impairment; hence dose adjustment may be needed. Taken together all these pharmacokinetic considerations and given that trametinib has not been evaluated in patients with hepatic dysfunction; it is of interest to investigate safety and pharmacokinetic assessments of trametinib in this patient population prior to routine use and dosing recommendations.

3. PATIENT SELECTION

3.1 Eligibility Criteria

3.1.1 Patients must have a histologically or cytologically confirmed solid malignancy that is metastatic or unresectable for which standard curative or palliative treatments do not exist or are no longer effective.

a. Hepatocellular carcinoma (HCC) patients are **not** required to have histologically or cytologically confirmed malignancy, patients are considered eligible based on tumor markers and/or imaging assessment.

Based on recent data that have shown limited trametinib benefit, patients with the following tumor types will be excluded from the **normal and mild cohorts**:

- Pancreatic cancer patients
- Colorectal cancer patients
- BRAF V600E melanoma patients who have failed BRAF inhibitors

Note: Patients with pancreatic cancer, colorectal cancer, and BRAF V600E melanoma patients who have failed BRAF inhibitors are **allowed** to enroll in the **moderate and severe cohorts** provided the patients: 1) sign a separate consent form which outlines the extremely limited activity observed in prior studies as mentioned in Section 2.3,

and 2) are consented to the study by a protocol-specified designee who is **not** their longitudinal oncologist.

3.1.2 All patients must have completed any prior chemotherapy, targeted therapy, radiotherapy (unless palliative doses which must be discussed with study Principal Investigator), surgery, anti-angiogenic therapy or interferon ≥ 28 days before study entry.

3.1.3 Age ≥18 years. Because no dosing or adverse event data are currently available on the use of trametinib in patients <18 years of age, children are excluded from this study, but will be eligible for future pediatric trials.

3.1.4 ECOG performance status ≤2 (Karnofsky ≥ 60%, see Appendix A).

3.1.5 Life expectancy of greater than 3 months.

3.1.6 Able to swallow and retain orally-administered medication and does not have any clinically significant gastrointestinal abnormalities that may alter absorption such as malabsorption syndrome or major resection of the stomach or bowels.

3.1.7 All prior treatment-related toxicities must be CTCAE v4.0 grade ≤1 (**except alopecia**) at the time of enrollment.

3.1.8 Patients must have normal organ (except liver function) and marrow function as defined below:

- Absolute neutrophil count (ANC) ≥1.2x10^9/L
- Hemoglobin ≥9 g/dL
- Platelets ≥75x10^9/L
- Serum creatinine ≤1.5 mg/dL (≤133 µmol/L) *OR* calculated creatinine clearance (Cockroft-Gault formula) ≥50 mL/min *OR* 24-hour urine creatinine clearance ≥50 mL/min
- Proteinuria ≤ +1 on dipstick or ≤1 gram/24 hours
- Prothrombin time (PT)/International normalized ratio (INR) and partial thromboplastin time (PTT) ≤1.5x institutional ULN
- Left ventricular ejection fraction (LVEF) ≥ institutional lower limit of normal (LLN) by ECHO or MUGA

3.1.9 Patients with abnormal hepatic function will be eligible and will be grouped according to criteria summarized below in Table 5 and in Section 5.1. Patients with active hemolysis should be excluded. No distinction should be made between liver dysfunction due to metastases and liver dysfunction due to other causes. This data will be captured in the Case Report Form (CRF). Registration laboratory investigations will be used to assign a patient to a hepatic function group. Hepatic function tests should be repeated within 24 hours prior to starting initial therapy and may result in patients' group assignment being altered if different to registration test results.

Table 5: Defining criteria for the four cohorts based on hepatic function.

Group	Hepatic Function	Hepatic Function
A	Normal hepatic function	Bilirubin ≤ ULN<br>AST ≤ ULN
B	Mild hepatic dysfunction	B1: bilirubin ≤ ULN and AST > ULN<br>B2: ULN < bilirubin ≤ 1.5x ULN and any AST
C	Moderate hepatic dysfunction	1.5x ULN < bilirubin ≤ 3x ULN and any AST
D	Severe hepatic dysfunction	3x ULN < bilirubin ≤ 10x ULN and any AST

3.1.10 Trametinib can cause fetal harm when administered to a pregnant woman. Women of child-bearing potential and men must agree to use adequate contraception (hormonal or barrier method of birth control; abstinence) prior to study entry, during the study participation, and for four months after the last dose of the drug. Women of child-bearing potential must have a negative serum pregnancy test within 14 days prior to registration and agree to use effective contraception throughout the treatment period and for 4 months after the last dose of study treatment. Should a woman become pregnant or suspect she is pregnant while she or her partner is participating in this study, she should inform her treating physician immediately.

3.1.11 Ability to understand and the willingness to sign a written informed consent document.

3.2 Exclusion Criteria

3.2.1 History of another malignancy.

Exception: Patients who have been disease-free for 3 years, or patients with a history of completely resected non-melanoma skin cancer and/or patients with indolent secondary malignancies, are eligible. Consult the CTEP Medical Monitor if unsure whether second malignancies meet the requirements specified above.

3.2.2 History of interstitial lung disease or pneumonitis.

3.2.3 Any major surgery, extensive radiotherapy, chemotherapy with delayed toxicity, biologic therapy, or immunotherapy within 28 days prior to enrollment and/or daily or weekly chemotherapy without the potential for delayed toxicity within 14 days prior to enrollment.

3.2.4 Use of other investigational drugs within 28 days (or five half-lives, whichever is shorter; with a minimum of 14 days from the last dose) preceding the first dose of trametinib and during the study. Patients previously treated with RAF and/or MEK inhibitors are excluded from the study. Multikinase antiangiogenic tyrosine kinase inhibitors such as regorafenib, sofarenib, sunitinib, etc. whose primary mechanism of action is not RAF inhibition, are allowed. If there are any questions, please contact study's Principal Investigator."

3.2.5 Symptomatic or untreated leptomeningeal or brain metastases or spinal cord compression.

3.2.6 Have a known immediate or delayed hypersensitivity reaction or idiosyncrasy to drugs chemically related to trametinib or excipients or to dimethyl sulfoxide (DMSO).

3.2.7 Current use of a prohibited medication. The following medications or non-drug therapies are prohibited:

- Other anti-cancer therapy while on study treatment. (Note: megestrol [Megace] if used as an appetite stimulant is allowed).
- Concurrent treatment with bisphosphonates is permitted; however, treatment must be initiated prior to the first dose of study therapy. Prophylactic use of bisphosphonates in patients without bone disease is not permitted, except for the treatment of osteoporosis.
- Because the composition, PK, and metabolism of many herbal supplements are unknown, the concurrent use of all herbal supplements is prohibited during the study (including, but not limited to, St. John's wort, kava, ephedra [ma huang], ginkgo biloba, dehydroepiandrosterone [DHEA], yohimbe, saw palmetto, or ginseng).

3.2.8 History or current evidence/risk of retinal vein occlusion (RVO).

3.2.9 History or evidence of cardiovascular risk including any of the following:

- LVEF<LLN.
- A QT interval corrected for heart rate using the Bazett's formula QTcB ≥480 msec.
- History or evidence of current clinically significant uncontrolled arrhythmias (exception: patients with controlled atrial fibrillation for >30 days prior to randomization are eligible).
- History of acute coronary syndromes (including myocardial infarction and unstable angina), coronary angioplasty, or stenting within 6 months prior to randomization.
- History or evidence of current ≥ Class II congestive heart failure as defined by the New York Heart Association (NYHA) functional classification system
- Treatment-refractory hypertension defined as a blood pressure of systolic >140 mmHg and/or diastolic >90 mmHg which cannot be controlled by anti-hypertensive therapy.
- Patients with intra-cardiac defibrillators

- Known cardiac metastases.

3.2.10 Active Hepatitis B Virus (HBV), or Hepatitis C Virus (HCV) infection (patients with chronic or cleared HBV and HCV infection are eligible). Patients with known HIV infection are eligible if not on antiviral agents and CD4 counts are adequate (≥ 500).

3.2.11 Uncontrolled inter-current illness including, but not limited to, ongoing or active infection, symptomatic congestive heart failure, unstable angina pectoris, cardiac arrhythmia, or psychiatric illness/social situations that would limit compliance with study requirements.

3.2.12 Animal reproductive studies have not been conducted with trametinib. Therefore, the study drug must not be administered to pregnant women or nursing mothers. Women of childbearing potential should be advised to avoid pregnancy and use effective methods of contraception. Men with a female partner of childbearing potential must have either had a prior vasectomy or agree to use effective contraception. If a female patient or a female partner of a patient becomes pregnant while the patient receives trametinib, the potential hazard to the fetus should be explained to the patient and partner (as applicable).

3.2.13 HIV-positive patients on combination antiretroviral therapy are ineligible because of the potential for pharmacokinetic interactions. In addition, these patients are at increased risk of lethal infections when treated with marrow-suppressive therapy. Appropriate studies will be undertaken in patients receiving combination antiretroviral therapy when indicated.

3.2.14 Any condition or medical problem in addition to the underlying malignancy and organ dysfunction which the investigator feels would pose unacceptable risk.

3.3 Inclusion of Women and Minorities

Both men and women of all races and ethnic groups are eligible for this trial. This study is designed to include minorities as appropriate. However, the trial is not designed to measure differences in intervention effects.

4. REGISTRATION PROCEDURES

4.1 Investigator and Research Associate Registration with CTEP

4.1.1 CTEP Registration Procedures

Food and Drug Administration (FDA) regulations and National Cancer Institute (NCI) policy require all investigators participating in any NCI-sponsored clinical trial to register and to renew their registration annually.

Registration requires the submission of:

- a completed ***Statement of Investigator Form*** (FDA Form 1572) with an original signature
- a current Curriculum Vitae (CV)
- a completed and signed ***Supplemental Investigator Data Form*** (IDF)
- a completed ***Financial Disclosure Form*** (FDF) with an original signature

Fillable PDF forms and additional information can be found on the CTEP website at http://ctep.cancer.gov/investigatorResources/investigator_registration.htm. For questions, please contact the ***CTEP Investigator Registration Help Desk*** by email at pmbregpend@ctep.nci.nih.gov.

4.1.2 CTEP Associate Registration Procedures / CTEP-IAM Account

The Cancer Therapy Evaluation Program (CTEP) Identity and Access Management (IAM) application is a web-based application intended for use by both Investigators (i.e., all physicians involved in the conduct of NCI-sponsored clinical trials) and Associates (i.e., all staff involved in the conduct of NCI-sponsored clinical trials).

Associates will use the CTEP-IAM application to register (both initial registration and annual re-registration) with CTEP and to obtain a user account.

Investigators will use the CTEP-IAM application to obtain a user account only. (See CTEP Investigator Registration Procedures above for information on registering with CTEP as an Investigator, which must be completed before a CTEP-IAM account can be requested.)

An active CTEP-IAM user account is needed to access all CTEP and CTSU (Cancer Trials Support Unit) websites and applications, and is critical to the conduct of this study, including document access, patient enrollment, and clinical data submission.

Additional information can be found on the CTEP website at
http://ctep.cancer.gov/branches/pmb/associate_registration.htm.
For questions, please contact the ***CTEP Associate Registration Help Desk*** by email at ctepreghelp@ctep.nci.nih.gov.

4.2 Site Registration

This study is supported by the NCI Cancer Trials Support Unit (CTSU).

Each investigator or group of investigators at a clinical site must obtain IRB approval for this protocol and submit IRB approval and supporting documentation to the CTSU Regulatory Office before they can be approved to enroll patients. Assignment of site registration status in the CTSU Regulatory Support System (RSS) uses extensive data to make a determination of whether a site has fulfilled all regulatory criteria including but not limited to: an active Federal Wide Assurance (FWA) number, an active roster affiliation with the Lead Network or a participating organization, a valid IRB approval, and compliance with all protocol specific requirements.

4.2.1 Downloading Regulatory Documents

Site registration forms may be downloaded from the 9591 protocol page located on the CTSU Web site. Permission to view and download this protocol is restricted and is based on person and site roster data housed in the CTSU RSS. To participate, Investigators and Associates must be associated with the Corresponding or Participating protocol organization in the RSS.

- Go to https://www.ctsu.org and log in using your CTEP IAM username and password
- Click on the Protocols tab in the upper left of your screen
- Either enter the protocol # in the search field at the top of the protocol tree, or
- Click on the By Lead Organization folder to expand, followed by LAO-11030, and protocol # 9591
- Click on LPO Documents sub-tab, select the Site Registration documents link, and download and complete the forms provided.

4.2.2 Submitting Regulatory Documents

Both U.S. and Canadian sites must submit their Model Informed Consent to the Study Coordinator at the University Health Network Princess Margaret Cancer Center (listed on protocol face page) for **pre**-approval prior to submitting to their local IRB/REB.

U.S. sites will submit completed forms along with a copy of the IRB Approval and Model Informed Consent to the CTSU Regulatory Office, where they will be entered and tracked in the CTSU RSS.

Regulatory Submission Portal: www.ctsu.org (members' area) → Regulatory Tab

→Regulatory Submission

When applicable, original documents should be mailed to:

CTSU Regulatory Office
1818 Market Street, Suite 1100
Philadelphia, PA 19103

Institutions with patients waiting that are unable to use the Portal should alert the CTSU Regulatory Office immediately at 1-866-651-2878 in order to receive further instruction and support.

Canadian sites will submit their REB initial approval along with the Model Informed Consent and all future amendment approvals to the University Health Network Princess Margaret Cancer Center for review. The University Health Network Princess Margaret Cancer Center will then send notification of document approval to the CTSU Regulatory Office for tracking and compliance in RSS.

Requirements for 9591 Site Registration:

- CTSU Transmittal Sheet (optional)
- IRB approval (For sites not participating via the NCI CIRB; local IRB documentation, an IRB-signed CTSU IRB Certification Form, Protocol of Human Subjects Assurance Identification/IRB Certification/Declaration of Exemption Form, or combination is accepted)

4.2.3 Checking Site Registration Status

You can verify your site registration status on the members' section of the CTSU website.

- Go to https://www.ctsu.org and log in to the members' area using your CTEP-IAM username and password
- Click on the Regulatory tab at the top of your screen
- Click on the Site Registration tab
- Enter your 5-character CTEP Institution Code and click on Go

Note: The status given only reflects compliance with IRB documentation and institutional compliance with protocol-specific requirements as outlined by the Lead Network. It does not reflect compliance with protocol requirements for individuals participating on the protocol or the enrolling investigator's status with the NCI or their affiliated networks.

4.3 Patient Registration

4.3.1 OPEN / IWRS

Patient enrollment will be facilitated using the Oncology Patient Enrollment Network (OPEN). OPEN is a web-based registration system available to users on a 24/7 basis. It is integrated with the CTSU Enterprise System for regulatory and roster data interchange and with the Theradex Interactive Web Response System (IWRS) for retrieval of patient registration/randomization assignment. Patient enrollment data entered by Registrars in OPEN / IWRS will automatically transfer to the NCI's clinical data management system, Medidata Rave.

For trials with slot reservation requirements, OPEN will connect to IWRS at enrollment initiation to check slot availability. Registration staff should ensure that a slot is available and secured for the patient before completing an enrollment.

The OPEN system will provide the site with a printable confirmation of registration and treatment information. Please print this confirmation for your records.

4.3.2 OPEN/IWRS User Requirements

OPEN/IWRS users must meet the following requirements:

- Have a valid CTEP-IAM account (*i.e.*, CTEP username and password).
- To enroll patients or request slot reservations: Be on an ETCTN Corresponding or Participating Organization roster with the role of Registrar.
- To approve slot reservations or access cohort management: Be identified to Theradex as the "Client Admin" for the study.
- Have regulatory approval for the conduct of the study at their site.

Prior to accessing OPEN/IWRS, site staff should verify the following:

- All eligibility criteria have been met within the protocol stated timeframes.
- If applicable, all patients have signed an appropriate consent form and HIPAA authorization form.

4.3.3 OPEN/IWRS Questions?

Further instructional information on OPEN is provided on the OPEN tab of the CTSU website at https://www.ctsu.org or at https://open.ctsu.org. For any additional questions contact the CTSU Help Desk at 1-888-823-5923 or ctsucontact@westat.com.

Theradex has developed a Slot Reservations and Cohort Management User Guide, which is available on the Theradex website:
http://www.theradex.com/clinicalTechnologies/?National-Cancer-Institute-NCI-11. This link to the Theradex website is also on the CTSU website OPEN tab. For questions about the use of IWRS for slot reservations, contact the Theradex Helpdesk: 609-619-7802 or

Theradex main number 609-799-7580; CTMSSupport@theradex.com.

4.4 General Guidelines

Following registration, patients should begin protocol treatment within 5 days. Issues that would cause treatment delays should be discussed with the Principal Investigator. If a patient does not receive protocol therapy following registration, the patient's registration on the study may be canceled. The Study Coordinator should be notified of cancellations as soon as possible. A record of patients who fail to meet entry criteria (*i.e.* screen failures) will be maintained.

5. TREATMENT PLAN

5.1 Stratification by Hepatic Function

5.1.1 Study Definition of Hepatic Dysfunction Group

Patients entering this study will be stratified into 4 groups or cohort according to their hepatic function as outline in the following table (Table 6):

Table 6: Defining criteria for the four cohorts based on hepatic function.

Group	Hepatic Function	Hepatic Function
A	Normal hepatic function	Bilirubin ≤ ULN<br>AST ≤ ULN
B	Mild hepatic dysfunction	B1: bilirubin ≤ ULN and AST > ULN<br>B2: ULN < bilirubin ≤ 1.5x ULN and any AST
C	Moderate hepatic dysfunction	1.5x ULN < bilirubin ≤ 3x ULN and any AST
D	Severe hepatic dysfunction	3x ULN < bilirubin ≤ 10x ULN and any AST

ULN= upper limit of normal

- Patients must fulfill both total bilirubin and SGOT/AST criteria to be included in a group. However, if a patient's total bilirubin level and SGOT/AST level indicate different groups, the patient may be enrolled in the indicated group with the greatest degree of liver dysfunction.

- No distinction will be made between liver dysfunction due to metastases and liver dysfunction due to other causes.

- All liver function tests must be completed within 24 hours prior to the start of treatment.

- Group B (mild): For the purposes of this study, the "mild" liver dysfunction may be defined according to either of two criteria (B1 and B2), so that patients in Group B may come from either of these groups. Patients in Groups B1 and B2 are thus considered to have comparable liver dysfunction and will be combined for dose level allocation and all analyses.

- Patients whose degree of hepatic dysfunction changes (becomes worse or better) between registration and initiation of protocol therapy may be re-assigned to a different dysfunction

group and dose level. This change should be discussed with the Principal Investigator. The Central Office Study Coordinator must document reassignments with notification to Theradex.

- Group A (normal): Patients in group A are included in this study as control subjects and will be followed for toxicity; however, the definitions of DLT in section 5.3 will not apply and a recommended dose will not be defined in these patients because the MTD has already been defined in such patients.

5.1.2 Child-Pugh Classification (CPC) of hepatic dysfunction

Each patient's CPC score (for liver dysfunction cohorts: B, C, and D) should be calculated at baseline and prior each treatment cycle. See Appendix B for instructions on CPC calculation.

5.2 Trametinib Dimethyl Sulfoxide (GSK1120212B, MEKINIST™) Administration

Treatment will be administered orally on an outpatient basis. Reported adverse events and potential risks are described in Section 7. Appropriate dose modifications are described in Section 6. No investigational or commercial agents or therapies other than those described below may be administered with the intent to treat the patient's malignancy.

Patients will receive trametinib taken orally once a day continuously of a 28-day cycle (Table 7).

Table 7: Dosing details for trametinib.

**Regimen Description**					
**Agent**	**Premedications; Precautions**	**Dose**	**Route**	**Schedule**	**Cycle Length**
Trametinib	Not required; Empty stomach (1hr before or 2hr after a meal)	To be assigned by central office	Oral	Daily	28 days (4 weeks)

To allow for liver function testing within 24 hours before drug administration and maximum PK sampling within a standard working week, the first dose of trametinib should be administrated on a Tuesday or Wednesday. However, for those institutions with resources able to obtain PKs on weekends, treatment may be started on other days.

Dose escalation will follow the schedule summarized in the table below (Table 8). All 4 liver dysfunction cohorts (A, B, C, D) may be open concomitantly.

NOTE: The patient will be requested to maintain a medication diary of each dose of medication (Appendix F). The medication diary will be returned to clinic staff at the end of each course.

Table 8: Dose escalation schema for each cohort as defined by hepatic function.

	**Group A**	**Group B**	**Group C**	**Group D**
	**Normal hepatic function**	**Mild hepatic dysfunction**	**Moderate hepatic dysfunction**	**Severe Hepatic dysfunction**
**Dose Level**	mg	mg	mg	mg
Level - 2	-	1	0.5	-
Level - 1	-	1.5	1	0.5
Level 1	2	2	1.5	1
Level +1	no escalation	no escalation	2	1.5
Level +2	no escalation	no escalation	no escalation	2

5.2.1 Trametinib

The effect of food on trametinib absorption is unknown. The current recommendation is to administer trametinib on an empty stomach, 1 hour before or 2 hours after a meal; the recommendation to administer trametinib fasting may change based on emerging data.

5.3 Definition of Dose-Limiting Toxicity

Toxicities will be graded according to the NCI Common Toxicity Criteria for Adverse Events version 4.0.0 (CTCAE v4.0.0). Dose-limiting toxicity (DLT) will be a toxicity that occurs during cycle 1 (first 28 days) and is felt to be possibly, probably or definitely related to the study drug.

The definition of DLT (Sections 5.3.1 and 5.3.2) and approaches for dose modification (Section 6) are provided in the protocol documents.

5.3.1 Hematologic Toxicity:

- Grade 3 or 4 neutropenia complicated by fever $\geq$38.5°C or infection
- Grade 4 neutropenia of at least 7 days duration
- Grade 3 thrombocytopenia complicated by hemorrhage
- Grade 4 thrombocytopenia.
  Note: Grade 4 lymphopenia, anemia, or leucopenia without neutropenia will NOT constitute a DLT. Grade 4 neutropenia that is disease related will not count as DLT

5.3.2 Non-Hematologic toxicity:

- Allergic reaction/hypersensitivity will not be considered dose-limiting
- Alopecia will not be considered dose limiting
- Any Grade 4 non-hematologic toxicity
- Grade 3 non-hematologic toxicity (except nausea, vomiting or diarrhea that can be controlled by appropriate medical intervention or prophylaxis and that resolves to Grade 1 within 48 hours with medical intervention or electrolyte toxicities unable to

be corrected to ≤ Grade 1 or baseline within 48 hours) excluding specific toxicities (***** see below)

- Treatment-related toxicities that result in failure to receive ≥75% of trametinib doses in cycle 1 despite maximal supportive care measures
- Delays in starting cycle 2 by ≥ 2 weeks due to treatment-related toxicity will constitute a DLT

* **Liver toxicity**: Worsening liver function, as defined by a rise in serum bilirubin, not related to tumor progression or stent occlusion will constitute a DLT if:

- For patients in the Hepatic Dysfunction cohorts (B, C, D):
Mild group (B): progresses into severe dysfunction range for 1 week or longer.
Moderate group (C): bilirubin increases from baseline ≥ 3 fold for 1 week or longer.
Severe group (D): bilirubin increases from baseline ≥ 2 fold for 1 week or longer.

Note: Patients requiring stent placement during study treatment will be allowed to continue study treatment after stent placement and a period of stabilization of clinical status and liver function tests. Two measurements at least 2 days apart will be accepted as evidence of stable hepatic function.

*** Cardiac related toxicity**: symptomatic cardiac failure; ≥ grade 3 LV systolic dysfunction reduction in LVEF ≥ 10 points from baseline and below the institution's lower limit of normal, without improvement within 2 weeks; QTc > 500 msec; other ≥ grade 3 cardiac disorders.

Management and dose modifications associated with the above adverse events are outlined in Section 6.

Dose Escalation Schema

Dose escalation will proceed within each cohort according to the following schema (Table 9).

Table 9: Dose escalation schema for each cohort as defined by hepatic function.

	**Group A**	**Group B**	**Group C**	**Group D**
	**Normal hepatic function**	**Mild hepatic dysfunction**	**Moderate hepatic dysfunction**	**Severe hepatic dysfunction**
**Dose Level**	mg	mg	mg	mg
Level - 2	-	1	0.5	-
Level - 1	-	1.5	1	0.5
Level 1	2	2	1.5	1
Level +1	no escalation	no escalation	2	1.5
Level +2	no escalation	no escalation	no escalation	2

Dose-limiting toxicity (DLT) is defined above (Sections 5.3.1. and 5.3.2).

The patient's starting dose will be assigned by the Central Office Study Coordinator at the time of registration. The patients' hepatic dysfunction group may be altered after registration if hepatic function tests performed within 24 hours of commencing trametinib have changed from registration results as described in section 5.1.1. The dose may be reduced for individual patients in subsequent cycles depending on toxicity as outlined in section 6.

Normal, mild, moderate and severe liver dysfunction cohorts may be open concurrently.

The initial cohorts of patients will begin on dose level 1 based on their level of hepatic dysfunction. Liver function tests (AST and total bilirubin) should be repeated within 24 hours prior to starting treatment on study to determine hepatic dysfunction cohort.

Escalation is planned as the standard "3+3" schema, for each hepatic dysfunction cohort (Cohorts B, C and D), with the exception of the normal hepatic function cohort (Cohort A) (Table 10).

Table 10: Dose escalation and cohort expansion rules using the standard 3+3 design.

Number of Patients with DLT at a Given Dose Level	Escalation Decision Rule
0 out of 3	Enter 3 patients at the next dose level.
≥ 2	Dose escalation will be stopped. This dose level will be declared the maximally administrated dose (highest dose administrated). Three (3) additional patients will be entered at the next lowest dose level if only 3 patients were treated previously at that dose.
1 out of 3	Enter at least 3 more patients at this dose level.<br>• If 0 of these 3 patients experience DLT, proceed to the next dose level.<br>• If 1 or more of this group suffer DLT, then dose escalation is stopped, and this dose is declared the maximally administrated dose. Three (3) additional patients will be entered at the next lowest dose level if only 3 patients were treated previously at that dose.
≤ 1 out of 6 at highest dose level below the maximally administrated dose	This is generally the recommended phase 2 dose. At least 6 patients must be entered at the recommended phase 2 dose.

No more than 12 patients will be enrolled to the normal hepatic function cohort, which serves as a pharmacokinetic comparison. For safety reasons, patients in the severe dysfunction cohort will be enrolled one patient at a time (i.e., the second patient in the severe cohort will not be enrolled until the first patient completes cycle 1 and so on for each subsequent patient). This one by one rule applies only while the same dose level for the moderate hepatic dysfunction group is incomplete (i.e. if moderate cohort at 1.5 mg/day of trametinib is not completed, severe cohort can be opened at the same dose level but no more than 1 patient can be enrolled until the same dose level in the moderate cohort has been cleared); once complete, we can enroll to the severe cohort as usual (see also Section 13.2). Patients with normal and mild hepatic function will not have their dose escalated.

Doses in more severe cohorts will not be escalated beyond doses being tested in less severe cohorts. If DLT-level toxicity is noted in dose level 1, then dose de-escalation to level -1 may occur (except for the normal hepatic function cohort). A patient may be dose reduced to the next lowest dose level if they experience and recover from a Grade 4 adverse event as outline in Section 6, but this should be discussed first with the Principal Investigator.

5.4 General Concomitant Medication and Supportive Care Guidelines

Because there is a low potential for interaction of trametinib with other concomitantly administered drugs through the cytochrome P450 system, the case report form must capture the concurrent use of all other drugs, over-the-counter medications, or alternative therapies. The Principal Investigator should be alerted if the patient is taking any agent known to affect or with the potential to affect selected CYP450 isoenzymes.

5.5 Duration of Therapy

In the absence of treatment delays due to adverse event(s), treatment may continue until one of the following criteria applies:

- Disease progression,
- Intercurrent illness that prevents further administration of treatment,
- Unacceptable adverse event(s),
- Patient decides to withdraw from the study, or
- General or specific changes in the patient's condition render the patient unacceptable for further treatment in the judgment of the investigator.

5.6 Duration of Follow Up

Patients will be followed for 4 weeks after removal from study or until death, whichever occurs first. Patients removed from study for unacceptable adverse event(s) will be followed until resolution or stabilization of the adverse event.

5.7 Criteria for Removal from Study

Patients will be removed from study when any of the criteria listed in Section 5.5 applies. The reason for study removal and the date the patient was removed must be documented in the Case Report Form.

6. DOSING DELAYS/DOSE MODIFICATIONS

The start (day 1) of subsequent cycles after cycle 1 can be delayed by up to three to four weeks (based on the adverse event causing the delay, as specified in this Section), but missed doses due to delays are not made up.

Dose reductions for toxicities not meeting DLT criteria may be considered for individual patients but only after consultation with the Principal Investigator. There is a maximum of 2 dose reductions per patient, after which they will be removed from the study. Dose reductions below 0.5 mg QD are not allowed.

6.1 Trametinib Dimethyl Sulfoxide (GSK1120212B, MEKINIST™) Dose Modifications

The table (Table 11) below outlines the dose levels to be used for any necessary trametinib dose modifications:

Table 11: Dose modification schema for each cohort as defined by hepatic function.

	**Group A**	**Group B**	**Group C**	**Group D**
	**Normal hepatic function**	**Mild hepatic dysfunction**	**Moderate hepatic dysfunction**	**Severe hepatic dysfunction**
**Dose Level**	mg	mg	mg	mg
Level - 2	1*	1	0.5	-
Level - 1	1.5*	1.5	1	0.5
Level 1	2	2	1.5	1
Level +1	no escalation	no escalation	2	1.5
Level +2	no escalation	no escalation	no escalation	2

*To be used for dose modifications only

A maximum of two trametinib dose level reductions are allowed. If a third dose level reduction is required, treatment will be permanently discontinued.

If a dose reduction of trametinib is required, but the toxicity resolves and no additional toxicities are seen after two cycles of treatment, the dose of trametinib may be re-escalated but should not exceed 2 mg once a day.

Dose modification and AE guidelines are outlined in the sections below for AEs that are deemed possibly related to trametinib:

- AEs not otherwise specified
- Rash
- Visual changes
- Diarrhea
- Liver chemistry elevation
- Ejection fraction changes

- Hypertension
- Prolonged QTc
- Pneumonitis

6.1.1 Trametinib Dose Modification for Toxicities Not Specified in Subsequent Sections

Table 12: Dose modification for toxicities not specified in subsequent sections.

<table>
<tr><td colspan="3">Trametinib Treatment Modification for Clinically Significant Toxicities Deemed Related to Trametinib
(This section is not for specific AEs such as hypertension, rash, ejection fraction changes, pneumonitis, diarrhea, liver chemistry, QTc prolongation, or visual changes. Refer to other sections for these specific AEs).</td></tr>
<tr><th>CTCAE v4.0 Grade</th><th>Management Guideline</th><th>Dose Modification</th></tr>
<tr><td>Grade 1</td><td rowspan="4">Monitor as clinically indicated. Provide supportive care according to institutional standards</td><td>Continue trametinib at current dose level.</td></tr>
<tr><td>Grade 2 (tolerable)</td><td>• Interrupt treatment until resolution to grade 1 or baseline.
• Upon resolution, restart treatment at current dose level.</td></tr>
<tr><td>Grade 2 (intolerable) and Grade 3</td><td>• Interrupt treatment until resolution to grade 1 or baseline.
• Upon resolution to baseline or grade 1, restart with one level of dose reduction
• If the Grade 3 toxicity recurs, interrupt trametinib; When toxicity resolves to Grade 1 or baseline, restart trametinib reduced by another dose level</td></tr>
<tr><td>Grade 4</td><td>If the event resolves to grade 1 or baseline discuss potential continuation of trametinib with Medical Monitor; if continuation of treatment agreed then restart trametinib at dose reduced by one dose level.
If the event does not resolve, permanently discontinue trametinib.</td></tr>
<tr><td colspan="3">Trametinib should be discontinued if treatment delay is ≥21 days due to toxicities. If the investigator concludes that continued trametinib will benefit a patient, the study chair and CTEP Medical Monitor may be consulted for the possibility of resuming trametinib, provided that toxicities have resolved to baseline or grade 1.</td></tr>
</table>

6.1.2 Trametinib Dose Modification for Rash

Rash is a frequent AE observed in patients receiving trametinib[19]. Recommendations for supportive care and guidelines for dose modifications for rash are based on experience with other MEK inhibitors and EGFR inhibitors[41,42].

The institutional standards for the management of skin-related AEs can differ from these guidelines. In this case, best clinical judgment should be applied and a consultation with the study chair or the CTEP Medical Monitor may be required.

Table 13: Dose modification and guidelines for supportive care of rash.

**Guidelines for Supportive Care of Rash**	
**Type of Care**	**Action**
**Prevention/Prophylaxis**[a]	• Avoid unnecessary exposure to sunlight.<br>• Apply broad-spectrum sunscreen (containing titanium dioxide or zinc oxide) with a skin protection factor (SPF) ≥15 at least twice daily.<br>• Use thick, alcohol-free emollient cream (*e.g.*, glycerine and cetomacrogol cream) on dry areas of the body at least twice daily.<br>• Topical steroids and antibiotics should be applied at least twice daily, starting on Day 1 of study treatment, to body areas such as face, chest, and upper back.<br>• Use mild-strength topical steroid (hydrocortisone 1% cream) or topical antibiotic (*e.g.*, clindamycin) or oral antibiotics (*e.g.*, doxycycline 100 mg BID, minocycline 100 mg BID).
**Symptomatic Care**[b]	• Pruritic lesions: Cool compresses and oral antihistamine therapies.<br>• Fissuring lesions: Monsel's solution, silver nitrate, or zinc oxide cream.<br>• Desquamation: Thick emollients and mild soap.<br>• Paronychia: Antiseptic bath, local potent corticosteroids in addition to antibiotics; if no improvement, consult dermatologist or surgeon.<br>• Infected lesions: Appropriate bacterial/fungal culture-driven systemic or topical antibiotics.
[a] Rash prophylaxis is recommended for the first 6 weeks of study treatment.<br>[b] Patients who develop rash/skin toxicities should be seen by a qualified physician and should receive evaluation for symptomatic/supportive care management.	

**Trametinib Dose Modification Guidelines and Management for Rash**		
**Rash Severity**	**Management Guideline**	**Dose Modification**
**Grade 1**	• Initiate prophylactic and symptomatic treatment measures.[1]<br>• Use moderate strength topical steroid.[2]<br>• Reassess after 2 weeks.	• Continue trametinib.

Trametinib Dose Modification Guidelines and Management for Rash		
**Rash Severity**	**Management Guideline**	**Dose Modification**
**Grade 2**	• Initiate prophylactic and symptomatic treatment measures.[1]<br>• Use moderate strength topical steroid.[2]<br>• Reassess after 2 weeks.	• **Reduce trametinib by one dose level.**<br>• If rash recovers to ≤ grade 1 within 2 weeks, increase dose to previous dose level.<br>• If no recovery to ≤ grade 1 within 2 weeks, interrupt trametinib until recovery to ≤ grade 1.<br>• **Restart trametinib at reduced dose level.**[3]
**Grade ≥3**	• Use moderate strength topical steroids PLUS oral methyl-prednisolone dose pack.[2]<br>• Consult dermatologist.	•Interrupt trametinib until rash recovers to ≤ grade 1.<br>• **Restart with trametinib reduced by one dose level.**[3,4]<br>• If no recovery to ≤ grade 2 within 4 weeks, **permanently discontinue trametinib**.
1. Rash prophylaxis is recommended for the first 6 weeks of study treatment.<br>2. Moderate-strength topical steroids: Hydrocortisone 2.5% cream or fluticasone priopionate 0.5% cream.<br>3. Approval of CTEP Medical Monitor is required to restart study treatment after >4 weeks of interruption.<br>4. Trametinib may be escalated to previous dose level if no rash is evident 4 weeks after restarting study treatment.		

6.1.3 Trametinib Dose Modifications for Visual Changes

Trametinib is known to be associated with visual adverse events. An ophthalmologist should be consulted if changes in vision develop. However, if the visual changes are clearly unrelated to study treatment (e.g., allergic conjunctivitis), then monitor closely as it may be reasonable to defer ophthalmic examination. Special attention should be given to retinal findings (*e.g.*, retinal pigment epithelial detachment (RPED) or retinovascular abnormalities (*i.e.*, branch or central retinal vein occlusions [RVO]). For events of visual changes regardless of severity but for which an ophthalmic examination is conducted, a blood sample for PK analysis is encouraged when feasible, and the blood sample should be drawn as close as possible to the time of the event.

The ophthalmology exam will include best corrected visual acuity, visual field examination, tonometry, slit lamp biomicroscopic examination, and indirect fundoscopy. Optical coherence tomography is recommended if retinal abnormalities are suspected. Other types of ancillary testing including visual field examination, fundus photography, and fluorescein angiography may also be indicated as determined by clinical exam.

Guidelines regarding event management and dose reduction for visual changes considered to be related to study treatment are provided in the table below.

Table 14: Dose modification and guidelines for supportive care of visual changes

**Management and Trametinib Dose Modification for Visual Changes and/or Ophthalmic Examination Findings**		
**Event CTCAE Grade**	**Management Guideline**	**Dose Modification**
**Grade 1***	• Consult ophthalmologist within 7 days of onset.	• If dilated fundus examination cannot be performed within 7 days of onset, hold trametinib until RPED and RVO can be excluded by retina specialist/ophthalmologist.<br>• If RPED and RVO excluded, continue/or restart trametinib at same dose level.<br>• If RPED suspected/diagnosed: See RPED dose modification table below (following this table); **report as SAE**.<br>• If RVO diagnosed: **Permanently discontinue trametinib and report as SAE**.
**Grade 2 and Grade 3**	• Consult ophthalmologist immediately.	• Hold trametinib<br>• If RPED or RVO excluded, restart trametinib at same dose level after visual AE is ≤ grade 1. If no recovery within 3 weeks, discontinue trametinib<br>• If RPED diagnosed: See RPED dose modification table below; **report as SAE**.<br>• If RVO: **Permanently discontinue trametinib and report as SAE**.
**Grade 4**	• Consult ophthalmologist immediately.<br>• Report as SAE.	• Hold Trametinib<br>• If RPED/RVO excluded, may restart trametinib at same or reduced dose after discussion with the CTEP Medical Monitor.<br>• **If RVO or RPED, permanently discontinue trametinib**.
Abbreviations: RPED = retinal pigment epithelial detachments; RVO = retinal vein occlusion; SAE = serious adverse event<br>*If visual changes are clearly unrelated to study treatment (e.g., allergic conjunctivitis), monitor closely but ophthalmic examination is not required.		

**Trametinib Dose Modification for RPED**	
**Event CTCAE Grade**	**Action and Dose Modification**
**Grade 1 RPED** (Asymptomatic; clinical or diagnostic observations only)	• Continue treatment with retinal evaluation monthly until resolution. If RPED worsens, follow instructions below.
**Grade 2-3 RPED** (Symptomatic with mild to moderate decrease in visual acuity; limiting instrumental ADL)	• Interrupt trametinib.<br>• Retinal evaluation monthly.<br>• If improved to ≤ Grade 1, restart trametinib with one dose level reduction (reduced by 0.5 mg) or discontinue in patients taking trametinib 1 mg daily.<br>• If no recovery within 4 weeks permanently discontinue trametinib

6.1.4 Trametinib Dose Modification for Diarrhea

Episodes of diarrhea have occurred in patients receiving trametinib[19]. Other frequent causes of diarrhea may include concomitant medications (*e.g.*, stool softeners, laxatives, antacids, *etc.*), infections by *C. difficile* or other pathogens, or partial bowel obstruction. Those conditions should be excluded.

Guidelines regarding management and dose modification for diarrhea considered related to trametinib are provided in the table below.

Table 15: Dose modification and guidelines for supportive care of diarrhea.

**Management and Trametinib Dose Modification Guidelines for Diarrhea**		
**CTCAE Grade**	**Adverse Event Management**	**Action and Dose Modification**
**Uncomplicated Diarrhea,[1]**<br>**Grade 1 or 2**	• Diet: Stop all lactose containing products; eat small meals, BRAT-diet (banana, rice, apples, toast) recommended.<br>• Hydration: 8-10 large glasses of clear liquids per day (*e.g.*, Gatorade or broth).<br>• Loperamide[3]: Initially 4 mg, followed by 2 mg every 4 hours or after every unformed stool; maximum 16 mg/day. Continue until diarrhea-free for 12 hours.<br>• Diarrhea >24 hours: Loperamide 2 mg every 2 hours; maximum 16 mg/day. Consider adding oral antibiotics.<br>• Diarrhea >48 hous: Loperamide 2 mg every 2 hours; maximum 16 mg/day. Add budesonide or other second-line therapies (otreotide, or tincture of opium) and oral antibiotics.	• Continue trametinib.<br>• If diarrhea is grade 2 for > 48 h, interrupt trametinib until diarrhea resolves to grade ≤1.<br>• Restart trametinib at the same dose level<br>• If treatment delay is >21 days, discontinue trametinib.

Management and Trametinib Dose Modification Guidelines for Diarrhea		
**CTCAE Grade**	**Adverse Event Management**	**Action and Dose Modification**
**Uncomplicated Diarrhea,[1] Grade 3 or 4**<br><br>**Any Complicated Diarrhea[2]**	• Clinical evaluation mandatory.<br>• Loperamide[3]: Initially 4 mg, followed by 2 mg every 4 hours or after every unformed stool; maximum 16 mg/day. Continue until diarrhea-free for 12 hours.<br>• Oral antibiotics and second-line therapies if clinically indicated.<br>• Hydration: Intravenous fluids if clinically indicated.<br>• Antibiotics (oral or intravenous) if clinically indicated.<br>• Intervention should be continued until the subject is diarrhea-free for ≥24 hours.<br>• Intervention may require hospitalization for subjects at risk of life-threatening complications.	• Interrupt trametinib until diarrhea resolves to ≤ grade 1.<br>• Restart with trametinib reduced by one dose level.[4]<br>• If 3 dose reductions of study treatment are clinically indicated, **permanently discontinue trametinib**.<br>• If treatment delay is >4 weeks, discontinue trametinib.
1. **Uncomplicated diarrhea** defined by the absence of symptoms such as cramping, nausea/vomiting, ≥ grade 2, decreased performance status, pyrexia, sepsis, neutropenia ≥ grade 3, frank bleeding, and/or dehydration requiring intravenous fluid substitution.<br>2. **Complicated diarrhea** defined by the presence of symptoms such as cramping, nausea/vomiting, ≥ grade 2, decreased performance status, pyrexia, sepsis, neutropenia ≥ grade 3, frank bleeding, and/or dehydration requiring intravenous fluid substitution.<br>3. Loperamide should be made available prior to start of study treatment so loperamide administration can begin at the first signs of diarrhea.<br>4. Escalation of trametinib to previous dose level is allowed after consultation with the medical monitor and in the absence of another episode of complicated or severe diarrhea in the 4 weeks subsequent to dose reduction.		

6.1.5 Trametinib Dose Modification for Liver Chemistry Changes

- Hepatic function tests must be repeated within 72 hours of initiation of cycle 2 and subsequent cycles.

For patients in the Normal hepatic function (A) Cohort:

- Liver chemistry stopping criteria are defined as follows. When any of the liver chemistry stopping criteria is met, immediately discontinue trametinib, perform liver event follow-up assessments, and monitor the patient until liver chemistries resolve, stabilize, or return to baseline values.
  - Liver chemistry stopping criteria are as follows:
    ALT ⩾3x ULN and bilirubin ⩾2x ULN (>35% direct bilirubin) (or ALT ⩾ 3x ULN and international normalized ratio [INR] >1.5, if INR measured). NOTE: If serum bilirubin fractionation is not immediately available, trametinib should be discontinued if ALT ⩾3x ULN and bilirubin ⩾ 2x ULN. Serum bilirubin fractionation should be performed if testing is available. If testing is unavailable, record presence of detectable urinary bilirubin on dipstick, indicating direct bilirubin elevations and suggesting liver

injury.
- ALT ⩾5x ULN.
- ALT ⩾3x ULN if associated with the appearance or worsening of symptoms of hepatitis or hypersensitivity such as fatigue, nausea, vomiting, right upper quadrant pain or tenderness, fever, rash, or eosinophilia.
- ALT ⩾3x ULN persisting for ⩾4 weeks.
- ALT ⩾3x ULN and cannot be monitored weekly for 4 weeks.

For patients in the Mild hepatic function (B):

- Liver chemistry stopping criteria are as follows:
  - If bilirubin > 3X ULN any time, hold trametinib. Treatment may resume under the following conditions:
    - The bilirubin returns to the range of mild dysfunction within 2 weeks. The patient will be treated at one dose level lower unless they are already dose reduced, in which case they will be taken off treatment.
    - If bilirubin does not return to treatable range within 2 weeks, patient will be off trametinib.

For patients in the Moderate hepatic function (C) or Severe hepatic dysfunction (D) Cohorts:

- Treatment for an individual patient will stop immediately when the bilirubin rises to the level of DLT (Section 5.3.2). Treatment for that patient can resume after a DLT under the following conditions:
  - The bilirubin of a patient in the moderate cohort (C) returns to the range of moderate dysfunction range within 2 weeks. The patient will be treated at one dose level lower unless they are already dose reduced, in which case they will be taken off treatment.
  - The bilirubin of a patient in the severe cohort (D) returns to $\leq$ 1.2 x baseline bilirubin within 2 weeks. The patient will be treated at one dose level lower unless they are already dose reduced, in which case they will be taken off treatment.

  If the bilirubin does not return to within the above-stated ranges within 2 weeks, the patient will be taken off treatment.

- If a patient has worsening liver function that leads to a cohort change but does not constitute a DLT, they will be treated according to the dose level being used for their new cohort, except if that dose is the same or higher than the dose on which they have already been treated. If the dose of their new cohort is the same or higher, they will be treated at one dose level lower than the previous dose that they received.

- If a patient experiences an improvement in liver function during treatment, there will be no change in dose during an ongoing cycle, but treatment may resume at a

new dose level determined by the cohort that would apply based on their liver function, if there is continued improvement at the time of the next cycle.

6.1.6 Trametinib Dose Modification for Pneumonitis

Pneumonitis has been observed in patients receiving trametinib. To reduce the risk of pneumonitis, patients will be monitored closely for symptoms and evaluated with imaging and functional tests. Dose modification and supportive care guidelines for pneumonitis are described in the tables below.

Table 16: Dose modification and guidelines for supportive care of pneumonitis.

**Pneumonitis Guidelines for Trametinib Monotherapy**		
**CTCAE Grade**	**Adverse Event Management**	**Action and Dose Modification**
**Grade 1**	• CT scan (high-resolution with lung windows) recommended.<br>• Work-up for infection<br>• Monitoring of oxygenation via pulse-oximetry recommended<br>• Consultation with pulmonologist recommended	• Continue trametinib at current dose
**Grade 2**	• CT scan (high-resolution with lung windows) recommended.<br>• Work-up for infection<br>• Consult pulmonologist<br>• Pulmonary function tests – if < normal, repeat every 8 weeks until ≥ normal<br>• Bronchoscopy with biopsy and/or BAL recommended<br>• Symptomatic therapy including corticosteroids if clinically indicated	• Interrupt trametinib until recovery to grade ≤1<br>• Restart treatment with trametinib **reduced by one dose level**<br>• Escalation to previous dose level after 4 weeks may be considered after consultation with medical monitor<br>• If no recovery to grade ≤1 within 4 weeks, **permanently discontinue trametinib**
**Grade 3**	• Same as grade 2	• Interrupt trametinib until recovery to grade ≤1<br>• After consultation with medical monitor, trametinib may be restarted reduced by one dose level<br>• If no recovery to grade ≤1 within 4 weeks, **permanently discontinue trametinib**
**Grade 4**	• Same as grade 2	• **Permanently discontinue trametinib**

6.1.7 Trametinib Dose Modification for Reduced Left Ventricular Ejection Fraction

Decreases of the left ventricular ejection fraction (LVEF) have been observed in patients receiving trametinib. Therefore, ECHOs/MUGAs must be performed in regular intervals outlined in the Study Calendar. The same procedure (either ECHO or MUGA,

although ECHO is preferred) should be performed at baseline and at follow-up visit(s).

Table 17: Dose modification and guidelines for supportive care of LVEF decrease.

**Trametinib Dose Modification Guidelines and Stopping Criteria for LVEF Decrease**		
**Clinic**	**LVEF-drop (%) or CTCAE grade**	**Action and Dose Modification**
**Asymptomatic**	Absolute decrease of >10% in LVEF compared to baseline and ejection fraction below the institution's LLN.	• Interrupt trametinib and repeat ECHO/MUGA within 2 weeks.[a]<br>• If the LVEF **recovers** within 4 weeks (defined as LVEF ≥LLN and absolute decrease ≤10% compared to baseline):<br>– Consult with the CTEP trametinib medical monitor and request approval for restart.<br>– Restart treatment with trametinib at reduced dose by one dose level.[b]<br>– Repeat ECHO/MUGA 2, 4, 8, and 12 weeks after re-start; continue in intervals of 12 weeks thereafter.<br>• If LVEF **does not** recover within 4 weeks:<br>– Consult with cardiologist.<br>– **Permanently discontinue trametinib.**<br>– Report as SAE<br>– Repeat ECHO after 2, 4, 8, 12, and 16 weeks or until resolution.<br>– Consult with the CTEP trametinib medical monitor.[c]
**Symptomatic[b]**	• Grade 3: resting LVEF 39-20% or >20% absolute reduction from baseline<br>• Grade 4: Resting LVEF ≤20%.	• **Permanently discontinue trametinib.**<br>• **Report as SAE.**<br>• **Consult with cardiologist.**<br>• Repeat ECHO after 2, 4, 8, 12, and 16 weeks or until resolution.
[a] If ECHO/MUGA does not show LVEF recovery after 2 weeks, repeat ECHO/MUGA 2 weeks later.<br>[b] Escalation of trametinib to previous dose level can be considered if LVEF remains stable for 4 weeks after restarting of trametinib. Approval from the CTEP trametinib medical monitor is required.<br>[c] Symptoms may include: dyspnea, orthopnea, and other signs and symptoms of pulmonary congestion and edema.		

6.1.8 Trametinib Dose Modification for QTc Prolongation

Table 18: Dose modification and guidelines for supportive care of QTc prolongation

**Trametinib Withholding and Stopping Criteria for QTc Prolongation**	
**Prolongation***	**Action and Dose Modification**
• QTcB ≥501 msec, or<br>• Uncorrected QT >600 msec, or<br>• QTcB >530 msec for subjects with bundle branch block	• Interrupt study treatment until QTcB prolongation resolves to grade 1 or baseline.<br>• Test serum potassium, calcium, phosphorus, and magnesium. If abnormal, correct per routine clinical practice to within normal limits.<br>• Review concomitant medication usage for a prolonged QTc.<br>• Restart at current dose level.[b]<br>• **If the event does not resolve or recurs after restarting, permanently discontinue study treatment.**<br>•
Abbreviations: msec = milliseconds; QTcB = QT interval on electrocardiogram corrected using Bazett's formula<br>[a] Based on average QTc value of triplicate ECGs. For example, if an ECG demonstrates a prolonged QT interval, obtain two or more ECGs over a brief period, and then use the averaged QTc values of the three ECGs to determine if study treatments should be interrupted or discontinued.<br>[b] if the QTc prolongation resolves to grade 1 or baseline, the subject may resume study treatment if the investigator and the CTEP trametinib medical monitor agree that the subject will benefit from further treatment.	

6.1.9 Trametinib Dose Modification for Hypertension

Increases in blood pressure (BP) have been observed in patients receiving trametinib. Recommendations for BP monitoring and management are provided below.

Monitoring: All BP assessments should be performed under the following optimal conditions:

- The subject has been seated with back support, ensuring that legs are uncrossed and flat on the floor.
- The subject is relaxed comfortably for at least 5 minutes.
- Restrictive clothing has been removed from the cuff area, and the right cuff has been selected.
- The subject's arm is supported so that the middle of the cuff is at heart level.
- The subject remains quiet during the measurement.
- In subjects with an initial BP reading within the hypertensive range, a second reading should be taken at least 1 minute later, with the two readings averaged to obtain a final BP measurement. The averaged value should be recorded in the eCRF.
- Persistent hypertension is defined as an increase of systolic blood pressure (SBP) >140 mmHg and/or diastolic blood pressure (DBP) >90 mmHg in three

consecutive visits with blood pressure assessments from two readings as described above. Visits to monitor increased blood pressure can be scheduled independently from the per-protocol visits outlined in the study calendar. Ideally, subsequent blood pressure assessments should be performed within 1 week.

Table 19: Dose modification and guidelines for supportive care of hypertension.

**Management and Trametinib Dose Modification for Hypertension**		
**Event**	**Management Guideline**	**Dose Modification**
Definitions used in the table:<br>- Persistent hypertension: Hypertension detected in two separate readings during up to three subsequent visits.<br>- Well-controlled hypertension: Blood pressure of SBP ≤140 mmHg and DBP ≤90 mmHg in two separate readings during up to three subsequent visits.<br>- Symptomatic hypertension: Hypertension associated with symptoms (*e.g.*, headache, light-headedness, vertigo, tinnitus, episodes of fainting) that resolve after the blood pressure is controlled within the normal range.<br>- Asymptomatic hypertension: SBP >140 mmHg and/or DBP >90 mmHg in the absence of the above symptoms.		
**(Scenario A)**<br>• Asymptomatic and persistent SBP of ≥140 and <160 mmHg, or DBP ≥90 and <100 mmHg,<br>or<br>Clinically significant increase in DBP of 20 mmHg (but still below 100 mmHg).	• Adjust current or initiate new antihypertensive medication(s).<br>• Titrate antihypertensive medication(s) during the next 2 weeks to achieve well-controlled BP. If BP is not well-controlled within 2 weeks, consider referral to a specialist and go to scenario (B).	Continue trametinib at the current dose.
**(Scenario B)**<br>• Asymptomatic SBP ≥160 mmHg, or DBP ≥100 mmHg,<br>or<br>Failure to achieve well-controlled BP within 2 weeks in Scenario A.	• Adjust current or initiate new antihypertensive medication(s).<br>• Titrate antihypertensive medication(s) during the next 2 weeks to achieve well-controlled BP.	• Interrupt trametinib.<br>• Once BP is well-controlled, restart trametinib **reduced by one dose level**.[a]
**(Scenario C)**<br>• Symptomatic[c] hypertension<br>or<br>Persistent SBP ≥160 mmHg, or DBP ≥100 mmHg, despite antihypertensive medication and dose reduction of study treatment	• Adjust current or initiate new antihypertensive medication(s).<br>• Titrate antihypertensive medication(s) during the next 2 weeks to achieve well-controlled BP.<br>• Referral to a specialist for further evaluation and follow-up is recommended.	• Interrupt trametinib.<br>• Once BP is well-controlled, restart trametinib **reduced by one dose level**.[a]
**(Scenario D)**<br>Refractory hypertension unresponsive to above interventions or hypertensive crisis.	Continue follow-up per protocol.	**Permanently discontinue trametinib**.
[a] Escalation of trametinib to previous dose level can be considered if BPs remains well controlled for 4 weeks after restarting of trametinib. Approval from Medical Monitor is required.		

7. ADVERSE EVENTS: LIST AND REPORTING REQUIREMENTS

Adverse event (AE) monitoring and reporting is a routine part of every clinical trial. The following list of AEs (Section 7.1) and the characteristics of an observed AE (Section 7.2) will determine whether the event requires expedited reporting (via CTEP-AERS) **in addition** to routine reporting.

7.1 **Comprehensive Adverse Events and Potential Risks list (CAEPR)** for **Trametinib dimethyl sulfoxide (GSK1120212B, NSC 763093)**

The Comprehensive Adverse Events and Potential Risks list (CAEPR) provides a single list of reported and/or potential adverse events (AE) associated with an agent using a uniform presentation of events by body system. In addition to the comprehensive list, a subset, the Specific Protocol Exceptions to Expedited Reporting (SPEER), appears in a separate column and is identified with bold and italicized text. This subset of AEs (SPEER) is a list of events that are protocol specific exceptions to expedited reporting to NCI (except as noted below). Refer to the 'CTEP, NCI Guidelines: Adverse Event Reporting Requirements' http://ctep.cancer.gov/protocolDevelopment/electronic_applications/docs/aeguidelines.pdf for further clarification. *Frequency is provided based on 1111 patients.* Below is the CAEPR for Trametinib (GSK1120212B).

NOTE: Report AEs on the SPEER **ONLY IF** they exceed the grade noted in parentheses next to the AE in the SPEER. If this CAEPR is part of a combination protocol using multiple investigational agents and has an AE listed on different SPEERs, use the lower of the grades to determine if expedited reporting is required.

Table 20: CAEPR for Trametinib dimethyl sulfoxide (GSK1120212B, NSC 763093)

Version 2.6, October 10, 2019[1]

**Adverse Events with Possible Relationship to Trametinib (GSK1120212B) (CTCAE 5.0 Term) [n= 1111]**			**Specific Protocol Exceptions to Expedited Reporting (SPEER)**
**Likely (>20%)**	**Less Likely (<=20%)**	**Rare but Serious (<3%)**	
BLOOD AND LYMPHATIC SYSTEM DISORDERS			
	Anemia		***Anemia (Gr 3)***
CARDIAC DISORDERS			
		Heart failure	
		Left ventricular systolic dysfunction	
	Sinus bradycardia		
EYE DISORDERS			
	Blurred vision		
	Dry eye		
		Eye disorders - Other (chorioretinopathy also known as retinal pigment epithelial detachment)	

**Adverse Events with Possible Relationship to Trametinib (GSK1120212B) (CTCAE 5.0 Term) [n= 1111]**			**Specific Protocol Exceptions to Expedited Reporting (SPEER)**
**Likely (>20%)**	**Less Likely (<=20%)**	**Rare but Serious (<3%)**	
		Eye disorders - Other (retinal vein occlusion)	
	Eye disorders - Other (visual disorders)[2]		
		Papilledema	
	Periorbital edema		
GASTROINTESTINAL DISORDERS			
	Abdominal pain		***Abdominal pain (Gr 2)***
		Colitis	
		Colonic perforation	
	Constipation		***Constipation (Gr 2)***
Diarrhea			***Diarrhea (Gr 3)***
	Dry mouth		***Dry mouth (Gr 2)***
	Dyspepsia		***Dyspepsia (Gr 2)***
	Mucositis oral		***Mucositis oral (Gr 3)***
Nausea			***Nausea (Gr 3)***
	Vomiting		***Vomiting (Gr 3)***
GENERAL DISORDERS AND ADMINISTRATION SITE CONDITIONS			
	Chills		***Chills (Gr 2)***
	Edema face		
Fatigue			***Fatigue (Gr 3)***
	Fever		***Fever (Gr 2)***
Generalized edema[3]			***Generalized edema[3] (Gr 2)***
IMMUNE SYSTEM DISORDERS			
	Allergic reaction[4]		
INFECTIONS AND INFESTATIONS			
	Folliculitis		***Folliculitis (Gr 2)***
	Lung infection		
	Paronychia		***Paronychia (Gr 2)***
	Skin infection		***Skin infection (Gr 2)***
INVESTIGATIONS			
	Alanine aminotransferase increased		***Alanine aminotransferase increased (Gr 3)***
	Alkaline phosphatase increased		***Alkaline phosphatase increased (Gr 2)***
	Aspartate aminotransferase increased		***Aspartate aminotransferase increased (Gr 3)***
	CPK increased		
	Ejection fraction decreased		
METABOLISM AND NUTRITION DISORDERS			
	Anorexia		***Anorexia (Gr 3)***
	Dehydration		***Dehydration (Gr 3)***
	Hypoalbuminemia		
	Hypomagnesemia		***Hypomagnesemia (Gr 2)***
	Hyponatremia		***Hyponatremia (Gr 3)***
MUSCULOSKELETAL AND CONNECTIVE TISSUE DISORDERS			
	Arthralgia		
	Back pain		***Back pain (Gr 2)***

Adverse Events with Possible Relationship to Trametinib (GSK1120212B) (CTCAE 5.0 Term) [n= 1111]			Specific Protocol Exceptions to Expedited Reporting (SPEER)
**Likely (>20%)**	**Less Likely (<=20%)**	**Rare but Serious (<3%)**	
	Pain in extremity		*Pain in extremity (Gr 2)*
		Rhabdomyolysis	
NERVOUS SYSTEM DISORDERS			
	Dizziness		*Dizziness (Gr 2)*
	Headache		*Headache (Gr 2)*
RESPIRATORY, THORACIC AND MEDIASTINAL DISORDERS			
	Cough		*Cough (Gr 2)*
	Dyspnea		*Dyspnea (Gr 3)*
		Pneumonitis	
SKIN AND SUBCUTANEOUS TISSUE DISORDERS			
	Alopecia		*Alopecia (Gr 2)*
	Dry skin		*Dry skin (Gr 2)*
	Nail changes		
		Palmar-plantar erythrodysesthesia syndrome	
	Pruritus		*Pruritus (Gr 2)*
		Skin and subcutaneous tissue disorders - Other (drug reaction with eosinophilia and systemic symptoms [DRESS])	
Skin and subcutaneous tissue disorders - Other (rash)[5]			*Skin and subcutaneous tissue disorders - Other (rash)[5] (Gr 3)*
		Stevens-Johnson syndrome[6]	
VASCULAR DISORDERS			
	Hypertension		*Hypertension (Gr 3)*
		Thromboembolic event (venous)	
	Vascular disorders - Other (hemorrhage)[7]		

[1]This table will be updated as the toxicity profile of the agent is revised. Updates will be distributed to all Principal Investigators at the time of revision. The current version can be obtained by contacting PIO@CTEP.NCI.NIH.GOV. Your name, the name of the investigator, the protocol and the agent should be included in the e-mail.

[2]Visual disorders include visual disturbance that can be associated with conjunctival hemorrhage, corneal graft rejection, cyclitis, eye nevus, halo vision, iritis, macular edema, retinal hemorrhage, visual acuity reduced, visual impairment, and vitreous detachment.

[3]Generalized edema includes edema, lymphedema, and edema limbs.

[4]Hypersensitivity (allergic reactions) may present with symptoms such as fever, rash, increased liver function tests, and visual disturbances.

[5]Skin and subcutaneous tissue disorders - Other (rash) may include rash, rosacea, rash acneiform, erythematous rash, genital rash, rash macular, exfoliative rash, rash generalized, erythema, rash papular, seborrhoeic dermatitis, dermatitis psoriasiform, rash follicular, skin fissures, and skin chapped.

[6]Stevens-Johnson syndrome has been observed in patients treated with trametinib and dabrafenib combination.

[7]The majority of hemorrhage events were mild. Major events, defined as symptomatic bleeding in a critical area or organ (e.g., eye, GI hemorrhage, GU hemorrhage, respiratory hemorrhage), and fatal intracranial hemorrhages have been reported.

Adverse events reported on trametinib dimethyl sulfoxide (GSK1120212B) trials, but for which there is insufficient evidence to suggest that there was a reasonable possibility that trametinib dimethyl sulfoxide (GSK1120212B) caused the adverse event:

BLOOD AND LYMPHATIC SYSTEM DISORDERS - Disseminated intravascular coagulation; Febrile neutropenia; Leukocytosis
CARDIAC DISORDERS - Atrial fibrillation; Cardiac arrest; Myocardial infarction; Restrictive cardiomyopathy; Sinus tachycardia
EYE DISORDERS - Corneal ulcer; Eyelid function disorder; Flashing lights; Floaters; Glaucoma; Photophobia
GASTROINTESTINAL DISORDERS - Ascites; Duodenal ulcer; Esophageal necrosis; Esophageal ulcer; Esophagitis; Gastric hemorrhage[7]; Gastric ulcer; Gastritis; Gastrointestinal disorders - Other (intestinal obstruction); Gastrointestinal disorders - Other (pneumatosis intestinalis); Gastrointestinal fistula; Gingival pain; Hemorrhoidal hemorrhage[7]; Ileus; Obstruction gastric; Pancreatitis; Small intestinal obstruction
GENERAL DISORDERS AND ADMINISTRATION SITE CONDITIONS - Flu like symptoms; General disorders and administration site conditions - Other (axillary pain); Localized edema; Malaise; Non-cardiac chest pain; Pain
HEPATOBILIARY DISORDERS - Cholecystitis; Hepatic failure; Hepatic pain; Hepatobiliary disorders - Other (hepatic encephalopathy)
INFECTIONS AND INFESTATIONS - Biliary tract infection; Catheter related infection; Device related infection; Endocarditis infective; Enterocolitis infectious; Hepatitis viral; Infections and infestations - Other (abscess limb); Infections and infestations - Other (necrotizing fasciitis); Infections and infestations - Other (oral infection); Pharyngitis; Sepsis; Upper respiratory infection; Urinary tract infection
INJURY, POISONING AND PROCEDURAL COMPLICATIONS - Bruising
INVESTIGATIONS - Blood bilirubin increased; Blood lactate dehydrogenase increased; Creatinine increased; Electrocardiogram QT corrected interval prolonged; GGT increased; Lipase increased; Lymphocyte count decreased; Platelet count decreased; Serum amylase increased; White blood cell decreased
METABOLISM AND NUTRITION DISORDERS - Hyperglycemia; Hyperkalemia; Hyperphosphatemia; Hyperuricemia; Hypocalcemia; Hypoglycemia; Hypokalemia
MUSCULOSKELETAL AND CONNECTIVE TISSUE DISORDERS - Generalized muscle weakness; Muscle cramp; Musculoskeletal and connective tissue disorder - Other (compression fracture); Myalgia; Neck pain
NEOPLASMS BENIGN, MALIGNANT AND UNSPECIFIED (INCL CYSTS AND POLYPS) - Tumor hemorrhage[7]; Tumor pain
NERVOUS SYSTEM DISORDERS - Dysgeusia; Encephalopathy; Intracranial hemorrhage[7]; Lethargy; Nervous system disorders - Other (diplopia); Seizure; Somnolence; Stroke; Syncope; Transient ischemic attacks
PSYCHIATRIC DISORDERS - Anxiety; Confusion; Delirium; Depression; Hallucinations; Insomnia; Personality change
RENAL AND URINARY DISORDERS - Acute kidney injury; Cystitis noninfective; Dysuria; Hematuria; Proteinuria; Urinary incontinence
REPRODUCTIVE SYSTEM AND BREAST DISORDERS - Vaginal fistula; Vaginal hemorrhage[7]
RESPIRATORY, THORACIC AND MEDIASTINAL DISORDERS - Bronchopulmonary hemorrhage[7]; Hypoxia; Laryngeal edema; Oropharyngeal pain; Pleural effusion; Pneumothorax; Productive cough; Pulmonary hypertension; Respiratory failure; Sinus disorder
SKIN AND SUBCUTANEOUS TISSUE DISORDERS - Bullous dermatitis; Photosensitivity; Purpura; Skin and subcutaneous tissue disorders - Other (erythema nodosum); Skin ulceration; Urticaria
VASCULAR DISORDERS - Hematoma; Hot flashes; Hypotension

Note: Trametinib (GSK1120212B) in combination with other agents could cause an exacerbation of any

adverse event currently known to be caused by the other agent, or the combination may result in events never previously associated with either agent.

7.2 Adverse Event Characteristics

- CTCAE term (AE description) and grade: The descriptions and grading scales found in the revised NCI Common Terminology Criteria for Adverse Events (CTCAE) version 4.0 will be utilized until March 31, 2018 for AE reporting. CTCAE version 5.0 will be utilized for AE reporting beginning April 1, 2018. All appropriate treatment areas should have access to a copy of the CTCAE version 5.0. A copy of the CTCAE version 5.0 can be downloaded from the CTEP web site http://ctep.cancer.gov/protocolDevelopment/electronic_applications/ctc.htm

- For expedited reporting purposes only:
    - AEs for the agent that are ***bold and italicized*** in the CAEPR (*i.e.*, those listed in the SPEER column, Section 7.1) should be reported through CTEP-AERS only if the grade is above the grade provided in the SPEER.

- **Attribution** of the AE:
    - Definite – The AE *is clearly related* to the study treatment.
    - Probable – The AE *is likely related* to the study treatment.
    - Possible – The AE *may be related* to the study treatment.
    - Unlikely – The AE *is doubtfully related* to the study treatment.
    - Unrelated – The AE *is clearly NOT related* to the study treatment.

7.3 Expedited Adverse Event Reporting

7.3.1 Expedited AE reporting for this study must use CTEP-AERS (CTEP Adverse Event Reporting System), accessed via the CTEP Web site (http://ctep.cancer.gov). The reporting procedures to be followed are presented in the "NCI Guidelines for Investigators: Adverse Event Reporting Requirements for DCTD (CTEP and CIP) and DCP INDs and IDEs" which can be downloaded from the CTEP Web site (http://ctep.cancer.gov). These requirements are briefly outlined in the tables below (Section 7.3.3).

In the rare occurrence when Internet connectivity is lost, a 24-hour notification is to be made to CTEP by telephone at 301-897-7497. Once Internet connectivity is restored, the 24-hour notification phoned in must be entered electronically into CTEP-AERS by the original submitter at the site.

7.3.2 CTEP-AERS is programmed for automatic electronic distribution of reports to the following individuals: Study Coordinator of the Lead Organization, Principal Investigator, and the local treating physician. CTEP-AERS provides a copy feature for other e-mail recipients.

7.3.3 Expedited Reporting Guidelines

Use the NCI protocol number and the protocol-specific patient ID assigned during trial registration on all reports.

Note: A death on study requires both routine and expedited reporting regardless of causality, unless as noted below. Attribution to treatment or other cause must be provided.

Death due to progressive disease should be reported as **Grade 5 "Disease progression"** in the system organ class (SOC) "General disorders and administration site conditions. Evidence that the death was a manifestation of underlying disease (*e.g.*, radiological changes suggesting tumor growth or progression: clinical deterioration associated with a disease process) should be submitted.

Table 21: Phase 1 and Early Phase 2 Studies: Expedited Reporting Requirements for Adverse Events that Occur on Studies under an IND/IDE within 30 Days of the Last Administration of the Investigational Agent/Intervention [1, 2]

FDA REPORTING REQUIREMENTS FOR SERIOUS ADVERSE EVENTS (21 CFR Part 312)
NOTE: Investigators **MUST** immediately report to the sponsor (NCI) **ANY** Serious Adverse Events, whether or not they are considered related to the investigational agent(s)/intervention (21 CFR 312.64)
An adverse event is considered serious if it results in **ANY** of the following outcomes:

1) Death
2) A life-threatening adverse event
3) An adverse event that results in inpatient hospitalization or prolongation of existing hospitalization for ≥ 24 hours
4) A persistent or significant incapacity or substantial disruption of the ability to conduct normal life functions
5) A congenital anomaly/birth defect.
   Important Medical Events (IME) that may not result in death, be life threatening, or require hospitalization may be considered serious when, based upon medical judgment, they may jeopardize the patient or subject and may require medical or surgical intervention to prevent one of the outcomes listed in this definition. (FDA, 21 CFR 312.32; ICH E2A and ICH E6).

ALL SERIOUS adverse events that meet the above criteria MUST be immediately reported to the NCI via CTEP-AERS within the timeframes detailed in the table below.

Hospitalization	Grade 1 and Grade 2 Timeframes	Grade 3-5 Timeframes
Resulting in Hospitalization ≥ 24 hrs	10 Calendar Days	24-Hour 5 Calendar Days
Not resulting in Hospitalization ≥ 24 hrs	Not required	

NOTE: Protocol specific exceptions to expedited reporting of serious adverse events are found in the Specific Protocol Exceptions to Expedited Reporting (SPEER) portion of the CAEPR.

Expedited AE reporting timelines are defined as:

- "24-Hour; 5 Calendar Days" - The AE must initially be reported via CTEP-AERS within 24 hours of learning of the AE, followed by a complete expedited report within 5 calendar days of the initial 24-hour report.
- "10 Calendar Days" - A complete expedited report on the AE must be submitted within 10 calendar days of learning of the AE.

[1]Serious adverse events that occur more than 30 days after the last administration of investigational agent/intervention and have an attribution of possible, probable, or definite require reporting as follows:<br>**Expedited 24-hour notification followed by complete report within 5 calendar days for:**<br>• All Grade 3, 4, and Grade 5 AEs<br>**Expedited 10 calendar day reports for:**<br>• Grade 2 AEs resulting in hospitalization or prolongation of hospitalization<br><br>[2]For studies using PET or SPECT IND agents, the AE reporting period is limited to 10 radioactive half-lives, rounded UP to the nearest whole day, after the agent/intervention was last administered. Footnote "1" above applies after this reporting period.<br><br>Effective Date: May 5, 2011

7.4 Routine Adverse Event Reporting

All Adverse Events **must** be reported in routine study data submissions. **AEs reported through CTEP-AERS must also be reported in routine study data submissions.**

7.5 Secondary Malignancy

A secondary malignancy is a cancer caused by treatment for a previous malignancy (*e.g.*, treatment with investigational agent/intervention, radiation or chemotherapy). A secondary malignancy is not considered a metastasis of the initial neoplasm.
CTEP requires all secondary malignancies that occur following treatment with an agent under an NCI IND/IDE be reported via CTEP-AERS. Three options are available to describe the event:

- Leukemia secondary to oncology chemotherapy (*e.g.*, acute myelocytic leukemia [AML])
- Myelodysplastic syndrome (MDS)
- Treatment-related secondary malignancy

Any malignancy possibly related to cancer treatment (including AML/MDS) should also be reported via the routine reporting mechanisms outlined in each protocol.

7.6 Second Malignancy

A second malignancy is one unrelated to the treatment of a prior malignancy (and is **NOT** a metastasis from the initial malignancy). Second malignancies require **ONLY** routine reporting via CDUS unless otherwise specified.

8. PHARMACEUTICAL INFORMATION

A list of the adverse events and potential risks associated with the investigational agent administered in this study can be found in Section 7.1.

8.1 Trametinib dimethyl sulfoxide (GSK1120212B, MEKINIST™) (NSC 763093)

Chemical Name (IUPAC):
equimolecular combination of acetamide, N-[3-[3-cyclopropyl-5-[(2-fluoro-4-iodophenyl)amino]-3,4,6,7-tetrahydro6,8-dimethyl- 2,4,7-trioxopyrido[4,3-d]pyrimidin-1(2H)-yl]phenyl] with 1,1'-sulfinylbis[methane]

Other Names: trametinib, GSK1120212, JTP-74057, JTP-78296, JTP-75303, Mekinist

CAS Registry Number: 1187431-43-1

Classification: MEK inhibitor

Molecular Formula: $C_{26}H_{23}FIN_5O_4 \bullet C_2H_6OS$

M.W.: 693.53 (dimethyl sulfoxide solvate), 615.41 (anhydrous parent)

Approximate Solubility: Trametinib dimethyl sulfoxide is almost insoluble in water (<0.0001 mg/mL at 25° C)

Mode of Action: Trametinib dimethyl sulfoxide is a reversible, highly selective, allosteric inhibitor of mitogen-activated extracellular signal regulated kinase 1 (MEK1) and MEK2. Tumor cells commonly have hyperactivated extracellular signal-related kinase (ERK) pathways in which MEK is a critical component. Trametinib dimethyl sulfoxide inhibits activation of MEK by RAF kinases and MEK kinases.

Description: Trametinib dimethyl sulfoxide is a white to almost white powder.

How Supplied: Novartis supplies and CTEP, NCI, DCTD distributes 0.5 mg and 2 mg (as free base) tablets. Each investigationally-labeled bottle contains 32 tablets.

The tablet core contains mannitol, microcrystalline cellulose, hypromellose, croscarmellose sodium, magnesium stearate (non-animal), colloidal silicon dioxide and sodium lauryl sulfate.

- 0.5 mg tablets are yellow, modified oval, biconvex and film-coated. Aqueous film coating consists of hypromellose, titanium dioxide, polyethylene glycol, iron oxide yellow.
- 2 mg tablets are pink, round, biconvex and film-coated. Aqueous film coating consists of hypromellose, titanium dioxide, polyethylene glycol, polysorbate 80, iron oxide red.

Storage: Store tablets at 2°C - 8°C (36° F to 46° F) in the original bottle and dispense unopened bottles. Do not open bottles or repackage tablets or remove desiccant. Bottles should be protected from light and moisture.

If a storage temperature excursion is identified, promptly return trametinib to 2°C -8°C and quarantine the supplies. Provide a detailed report of the excursion (including

documentation of temperature monitoring and duration of the excursion) to PMBAfterHours@mail.nih.gov for determination of suitability.

Stability: Stability studies are ongoing. Tablets are only stable for 32 days once bottle has been opened. If multiple bottles are dispensed to a patient in the same visit, please advise the patient to open only one bottle at a time.

Route of Administration: Oral. Take by mouth on an empty stomach, either 1 hour before or 2 hours after a meal. If a dose of trametinib is missed, the dose can be taken if it is more than 12 hours until the next scheduled dose.

Potential Drug Interactions

In vitro studies suggest that trametinib dimethyl sulfoxide is not a substrate of CYP enzymes or of human BCRP, MRP2, OATP1B1, OATP1B3, OATP2B1, OCT1 or MATE1 transporters. Trametinib elimination by deacetylation to metabolite M5 is dependent on carboxylesterases (CES1b, CES1c and CES2). Trametinib is a substrate for P-gp and BSEP, but this is not expected to be clinically relevant due to trametinib's high permeability.

Trametinib dimethyl sulfoxide is an *in vitro* inhibitor of CYP 2C8, and is anticipated to have overall low potential for drug interactions as a perpetrator. It is also a weak CYP 2B6 and 3A4 inducer and expected to have little clinical effect on sensitive substrates. Trametinib is not an inhibitor of CYP 1A2, 2A6, 2B6, 2C9, 2C19, 2D6 and 3A4 and not an inhibitor of MRP2 or BSEP, but as in vitro inhibitor of P-gp, BCRP, OATP1B1, OATP1B3, OAT1, OAT3, OCT2 and MATE1 at systemic concentrations that are not clinically relevant. No clinically relevant inhibition by trametinib is predicted in the liver or kidney and a low risk of intestinal drug-drug interaction is possible with BCRP.

Patient Care Implications: Advise women study participants of reproductive potential to use effective contraception while receiving study treatment and for 4 months after the last dose of trametinib. Advise women not to breastfeed while receiving study treatment and for 4 months after the last dose of trametinib. Advise men study participants to use barrier contraception and not to father a child while taking study treatment and for 4 months after the last dose of trametinib.

Availability

Trametinib dimethyl sulfoxide (GSK1120212B) is an investigational agent supplied to investigators by the Division of Cancer Treatment and Diagnosis (DCTD), NCI.

Trametinib dimethyl sulfoxide (GSK1120212B) is provided to the NCI under a Collaborative Agreement between the Pharmaceutical Collaborator and the DCTD, NCI (see Section 12.3).

8.1.1 Agent Ordering and Agent Accountability

8.1.1.1 NCI-supplied agents may be requested by the Principal Investigator (or their authorized designee) at each participating institution. Pharmaceutical Management Branch (PMB) policy requires that agent be shipped directly to the institution where the patient is to be treated. PMB does not permit the transfer of agents between institutions (unless prior approval from PMB is obtained). The CTEP-assigned protocol number must be used for ordering all CTEP-supplied investigational agents. The responsible investigator at each participating institution must be registered with CTEP, DCTD through an annual submission of FDA Form 1572 (Statement of Investigator), Curriculum Vitae, Supplemental Investigator Data Form (IDF), and Financial Disclosure Form (FDF). If there are several participating investigators at one institution, CTEP-supplied investigational agents for the study should be ordered under the name of one lead investigator at that institution.

Active CTEP-registered investigators and investigator-designated shipping designees and ordering designees can submit agent requests through the PMB Online Agent Order Processing (OAOP) application (https://eapps-ctep.nci.nih.gov/OAOP/pages/login.jspx). Access to OAOP requires the establishment of a CTEP Identity and Access Management (IAM) account (https://eapps-ctep.nci.nih.gov/iam/) and the maintenance of an “active” account status and a “current” password. For questions about drug orders, transfers, returns, or accountability, call (240) 276-6575 Monday through Friday between 8:30 am and 4:30 pm (ET) or email PMBAfterHours@mail.nih.gov anytime.

8.1.1.2 Agent Inventory Records – The investigator, or a responsible party designated by the investigator, must maintain a careful record of the inventory and disposition of all agents received from DCTD using the NCI Drug Accountability Record Form (DARF). (See the NCI Investigator’s Handbook for Procedures for Drug Accountability and Storage.)

8.1.2 Investigator Brochure Availability

The current versions of the IBs for PMB-supplied agents will be accessible to site investigators and research staff through the PMB Online Agent Order Processing (OAOP) application. Access to OAOP requires the establishment of a CTEP Identity and Access Management (IAM) account and the maintenance of an “active” account status and a “current” password. Questions about IB access may be directed to the PMB IB coordinator at IBCoordinator@mail.nih.gov.

9. BIOMARKER, CORRELATIVE, AND SPECIAL STUDIES

9.1 Pharmacokinetic Studies

Pharmacokinetic studies will be performed on all enrolled patients to assess the bio-distribution of trametinib in patients with organ dysfunction. At the Principal Investigator’s discretion, this requirement may be waived in case of patient hardship, including lack of venous access. In this event, patients will be replaced to ensure that

adequate PK data are obtained for each group (i.e., at least 3 patients per group and at least 6 patients at the MTD level).

In blood, trametinib has been shown to be the predominant radio- component and M5 the only detectable minor component (<7% of the blood radioactivity). Plasma concentrations will be measured by Covance Laboratory Inc using a validated LC/MS/MS assay. All PK measurements for this study will be performed at Covance Laboratory Inc. All data and results will be made available to the investigators on this study and to CTEP. The minimum turnaround time for PK measurements will be 4 weeks from receipt of samples by the analytical laboratory. In patients who experience unexpected serious toxicity efforts will be made to have analysis available in 2 weeks. Availability of PK data is anticipated prior to dose escalation to another dose level.

PK sampling will be performed in cycle 1 for all patients. In patients with incomplete PK from cycle 1 and in those who change dose level or hepatic dysfunction group between cycles, repeat PK sampling is encouraged in subsequent cycles but is not mandatory. For severe liver dysfunction cohort, patients who complete PK schedule at day 16 will be considered evaluable for DLT assessment.

Specimen Collection and Analysis Details are provided in the "Pharmacokinetic Laboratory Manual" supplied by the Coordinating Central Office. Note: This will be disseminated with the clinical protocol and is also available to investigators on request from the coordinating center.

9.1.1 Specimen Collection

The blood samples will be collected into 2 mL K2-EDTA vacutainers for all time points except Day 15 predose and 2 hours. At those two points, blood samples will be collected into 5 mL K2-EDTA vacutainers due to extra plasma protein binding measurement. Fill all blood tube completely and mix immediately by gently inverting the tube 8-10 times. Place collected blood on wet ice *immediately* after mixing with EDTA.

Pharmacokinetic study will start on C1D15 of the trial. The times of drug administration on Day 15 and 16should be recorded on the specimen collection form.

9.1.2 Specimen Collection Schedule

Plasma will be collected on Day 15 of Cycle 1 according to the following schedule: before drug administration and at the following times after administering the oral dose: 0.5, 1, 2, 3, 4, 6, 10 and 24 hours (+/- 10 minute window). Thus, 9 samples will be collected in Cycle 1.

To evaluate trametinib plasma protein binding measurements, additional blood samples will be collected on Day 15before trametinib dose and 2 hours after trametinib dose (+/- 10 minute window).

Sampling Schedule is summarized in following Table (Table 22).

Table 22: Pharmacokinetic sample collection schedule.

Sample Number	Day of Collection	Planned Collection Time (hours)
1*	15	0 (Pretreatment)
2	15	0.5
3	15	1.0
4*	15	2.0
5	15	3.0
6	15	4.0
7	15	6.0
8	15	10
9	16	24

* 5mL of blood should be collected to generate 1.5 mL of plasma samples for pharmacokinetics and plasma protein binding analysis.

9.1.3 Specimen Processing Procedures

Blood samples stored on wet ice will be centrifuged *within 1 hour of collection* at approximately 1600 g for approximately 15 minutes under chilled conditions (approximately 4°C). For all time points except Day 15 predose and 2 hours, the resulting plasma will be transferred immediately into appropriately labeled 1.0-mL Matrix TrackMate ScrewTop tubes for PK analysis. For Day 15 predose and 2 hour time points, aliquot of 1.5 mL of plasma samples will be transferred immediately into labeled 3-mL Nunc tubes for plasma protein binding measurement; the remaining plasma samples (approximately 0.5 mL) will be transferred immediately to labeled 1.0 mL Matrix TrackMate ScrewTop tubes for PK analysis. All plasma samples will be placed on wet ice *immediately* after processing. Store samples frozen at minus 20°C (-20°C) or lower within 60 minutes of the sampling time. Ship frozen to Covance CLS. For additional details please refer to Appendix D.
Note: Do not fill the tube more than half full.

9.1.4 Specimen Shipping Instructions

Ship Pharmacokinetics samples to:

Sample Management-Bioanalytical (Rm 1S 160)
Covance Laboratory Inc.
3301 Kinsman Boulevard
Madison, WI 53704-2523
Madison.SA@Covance.com

Contact Information:
Mark Hoffman
mark.hoffmann@covance.com

9.1.5 Pharmacokinetic Analysis

The following trametinib PK parameters will be calculated based on results collected for each Cohort:

- Maximum plasma concentration (Cmax)
- Time to maximum concentration (Tmax)
- Steady state concentration (Css)
- Trough concentrations at steady-state ($C_{ss,min}$)
- Terminal half-life ($t_{1/2}$) clearance
- Area under the plasma concentration-time curve for 0-24 h or tau ($AUC_{0\text{-}24h/tau}$)

9.2 Biomarker Studies

Tumor molecular profiling exploratory analysis: The increased cancer biology knowledge and the availability of molecular profiling technologies have dramatically modified the current medical oncology treatment to a more targeted and personalized medicine approach[43,44]. Novel genomic sequencing technologies and platforms provide the opportunity to more comprehensively characterize molecular aberrations of human cancers for individual cancer patients. Most recently, new trials are currently evaluating the importance of performing tumor molecular analysis in cancer setting[45,46]. As an example, the Princess Margaret Cancer Centre Integrated Molecular Profiling in Advanced Cancers Trial (IMPACT) has shown the feasibility and the clinical relevance of integrating this approach into the routine care of cancer patients[47]. In the current study, targeted DNA sequencing will be performed in the CAP/CLIA certified laboratory of Dr. Suzanne Kamel-Reid, using the TruSeq® Amplicon – Cancer Panel (TSACP) for the MiSeq® System, which includes 48 genes and 212 amplicons (see below gene list, Table 23):

Table 23: TSACP Panel

**ABL1**	**EGFR**	**GNAS**	**MLH1**	**RET**
**AKT1**	**ERBB2**	**HNF1A**	**MPL**	**SMAD4**
**ALK**	**ERBB4**	**HRAS**	**NOTCH1**	**SMARCB1**
**APC**	**FBXW7**	**IDH1**	**NPM1**	**SMO**
**ATM**	**FGFR1**	**JAK2**	**NRAS**	**SRC**
**BRAF**	**FGFR2**	**JAK3**	**PDGFRA**	**STK11**
**CDH1**	**FGFR3**	**KDR**	**PIK3CA**	**TP53**

**CDKN2A**	**FLT3**	**KIT**	**PTEN**	**VHL**
**CSF1R**	**GNA11**	**KRAS**	**PTPN11**	
**CTNNB1**	**GNAQ**	**MET**	**RB1**	

In this study, the molecular profiles of formalin fixed paraffin embedded tumor specimens, as assessed using the TSACP panel, will not be considered integral or integrated tests and will be performed retrospectively for exploratory analysis. The results will be correlated with clinical outcome on trametinib. All results will be considered exploratory.

The clinical investigator and the submitting pathologist have the responsibility for submitting representative materials for the goals cited in the protocol.

Tissue requirement for mutation determination and confirmation is composed of either slides or blocks. Available formalin fixed paraffin embedded tissue blocks or slides must consist of: A routine H&E slide should be sent along with tissue blocks or unstained slides. The H&E will be reviewed for tissue to determine percent of tumor nuclei with appropriate area marked for macro dissection. If blocks are not available unstained slides can be provided. Ten unstained slides should be sequentially cut 4-5 micron sections and mount on uncharged slides and one routine H&E section. Slides will be coded. Ideally, each slide must have a minimum of 50% tumor tissue on the slide to be deemed adequate for study; although with macro dissection slides with lower tumor % will be considered. Do not bake or place cover slips on the slides. Slides should be placed in appropriate slide containers and labeled, shipping is at ambient temperature.

In this study, the molecular profiles of formalin fixed paraffin embedded tumor specimens, as assessed using the TSACP panel, will be correlated with clinical outcome on trametinib as exploratory biomarkers. All the results will be exploratory and descriptive; therefore no statistical significance will be concluded.

All study subjects are requested to provide 1 tube of whole blood at baseline (or anytime on study if baseline sample is missed). This will provide germline DNA for control purpose such that it is possible to distinguish between germline and somatic DNA to enable the analysis of the genomic data obtained using the TSACP panel.

Please refer to Laboratory Study Manual for processing and shipping information.

10. STUDY CALENDAR

Baseline evaluations are to be conducted within 1 week prior to start of protocol therapy. Scans and x-rays must be done $\leq$4 weeks prior to the start of therapy. **Liver function tests should be repeated within 24 hours to starting Cycle 1 Day 1.** In the event that the patient's condition is deteriorating, laboratory evaluations should be repeated within 48 hours prior to initiation of the next cycle of therapy. The schedules should be followed as closely as possible; however, the schedule may be modified (± 3 days) due to problems such as scheduling delays or conflicts (i.e.

clinic closure, weather conditions, vacations, etc.). Longer delays will require the guidance of the Principal Investigator as appropriate. Additional tests may be performed at the discretion of the treating investigator or site as per routine practice, or as otherwise clinically indicated.

Table 24: Study calendar.

	**Screen[1]**	**C1, D1**	**C1, D2**	**C1, D8**	**C1, D15**	**C1, D16**	**C1, D22**	**C2, D1**	**C2, D15**	**C3, D1**	**Every 2 weeks after C3, D1[2]**	**Every 4 weeks after C3, D1[2]**	**Every 8 weeks after C3, D1[2]**	**Every 12 weeks after C3, D1[2]**	**EOT[3]**	**F/U**	**END**
Informed Consent	X																
Baseline Demography	X																
Medical History	X																
Prior anti-cancer therapy & radiotherapy	X																
Registration		X															
**SAFETY**																	
Physical Examination	X	X			X			X	X	X	X				X		
Dermatologic Examination[4]	X																
ECOG Performance Status	X	X			X			X	X	X	X						
Vital Signs (BP, HR, T)	X	X		X	X		X	X	X	X	X				X		
Height and Weight[5]	X	X						X		X		X			X		
Hematology/Clinical Chemistry[6]	X	X†		X	X		X	X	X	X	X				X		
Females: Pregnancy Test[7]	X									X			X		X		
INR/PTT	X	X†						X		X		X					
12-lead ECG	X	Performed during treatment if clinically indicated															
Echocardiogram or MUGA*	X	Every 12 weeks (use same methodology for baseline and follow up)															
Ophthalmology exam[8]	X	Performed during treatment if clinically indicated															
Urinalysis	X				X			X		X		X					
Adverse Events	X	X		X	X		X	X	X	X	X				X	X	X
Child-Pugh Classification (CPC) assessment (not required in Group A (normal cohort))	X							X		X		X					
Concomitant Medication Check	X	X		X	X		X	X	X	X	X				X	X	X
**STUDY DRUG**																	
Dispense study medication trametinib, assess compliance (4 week supplies, except C1, weekly supply)		X		X	X		X	X		X		X					
**EFFICACY**																	
Disease characteristics[9]	X																

	**Screen**[1]	**C1, D1**	**C1, D2**	**C1, D8**	**C1, D15**	**C1, D16**	**C1, D22**	**C2, D1**	**C2, D15**	**C3, D1**	**Every 2 weeks after C3, D1**[2]	**Every 4 weeks after C3, D1**[2]	**Every 8 weeks after C3, D1**[2]	**Every 12 weeks after C3, D1**[2]	**EOT**[3]	**F/U**	**END**
Lesion Assessment (RECIST 1.1)	X	Tumor measurements are repeated every 2 cycles (approximately 8 weeks). Documentation (radiologic) must be provided for patients removed from study for progressive disease.														X	
Tumor marker(s) (if applicable)[10]	X							X		X			X		X		
**PHARMACOKINETICS/ PHARMACOGENOMICS**																	
Pharmacokinetic Sampling[11]					X	X											
Pharmacogenomic Blood Sampling[12, 13]	X																
**EXPLORATORY CORRELATIVE STUDIES**																	
Archival tumor tissue[13]	X																

Note: BP=Blood pressure; C=Cycle; D=Day; ECG=Electrocardiogram; ECOG=Eastern Cooperative Oncology Group; End=End of study participation; EOT=End of Treatment; F/U=follow-up; HR=Heart rate; T=Temperature; W=Week

† If baseline labs are within 1 week of the start of protocol therapy, labs do not need to be repeated on cycle 1 day 1. Only the liver function tests (AST and total bilirubin) to confirm the cohort assignment need to be repeated within 24 hours of cycle 1 day 1.

1. All screening assessments must be completed within 1 week prior to first dose except archival tumor tissue and imaging analyses
2. Safety assessments will continue every 2 weeks while the subject remains on study medication(s), with the exception of urinalysis which will be every 4 weeks, , ECHO/MUGA will be performed every 12 weeks unless clinically indicated. Efficacy assessments will occur every 2 cycles (approximately 8 weeks), unless required to confirm a response or as clinically indicated.
3. Discontinuation visit should be 14 days from last dose of study medication(s).
4. Dermatologic exams should be performed by the Investigator, or may be referred to a dermatologist, at the discretion of the investigator for patients. Dermatologic exam should be performed at baseline and then repeated as clinically indicated.
5. Height and weight at screening only; weight only at other indicated visits.
6. Hematology/Clinical Chemistry includes CBC with differential, sodium, chloride, potassium, bicarbonate, creatinine, AST, ALT, ALP, bilirubin, albumin, calcium, magnesium, and phosphate. Note: liver function tests must be performed 24 hours prior C1D1.
7. Serum pregnancy test should be performed within 14 days of administration of the first dose of study medication; all subsequent pregnancy tests may be either serum or urine.
8. Ophthalmology Exam: Fundoscopy, tonometry, visual field examination, slit lamp biomicroscopic examination, and corrected visual acuity assessments at baseline screening and when clinically indicated during study. Note: Optical coherence tomography is recommended if retinal abnormalities are suspected
9. Disease Characteristics: Record date of diagnosis, primary tumor type, histology, stage, etc.
10. Applicable tumor markers include CA125 (ovary), CA19-9 (ampullary, biliary), and CEA (small bowel).
11. Pharmacokinetic sampling: C1D15 (up to 10 hours), C1D16 (24 hours) (+/- 10 minute window).
12. One tube of whole blood will be obtained at C1D1 (or anytime during study) in all patients to provide a source of germline DNA.

13. PG blood sample and archival tissue collection are optional and subjects will choose if they wish to donate these samples by signing the optional genetic testing ICF; however donation of these samples are highly recommended

* the same modality (ECHO or MUGA) should be used at baseline and at follow-up

11. MEASUREMENT OF EFFECT

Although response is not the primary endpoint of this trial, patients with measurable disease will be assessed by standard criteria. For the purposes of this study, patients should be re-evaluated every 8 weeks. In addition to a baseline scan, confirmatory scans will also be obtained 4 weeks following initial documentation of an objective response.

11.1 Antitumor Effect – Solid Tumors

For the purposes of this study, patients should be re-evaluated for response every 2 cycles (approximately 8 weeks). In addition to a baseline scan, confirmatory scans should also be obtained 4 (not less than 4) weeks following initial documentation of objective response.

Response and progression will be evaluated in this study using the new international criteria proposed by the revised Response Evaluation Criteria in Solid Tumors (RECIST) guideline (version 1.1)[48]. Changes in the largest diameter (unidimensional measurement) of the tumor lesions and the shortest diameter in the case of malignant lymph nodes are used in the RECIST criteria.

11.1.1 Definitions

<u>Evaluable for toxicity</u>. All patients will be evaluable for toxicity from the time of their first treatment with trametinib.

<u>Evaluable for objective response.</u> Only those patients who have measurable disease present at baseline, have received at least one cycle of therapy, and have had their disease re-evaluated will be considered evaluable for response. These patients will have their response classified according to the definitions stated below. (Note: Patients who exhibit objective disease progression prior to the end of cycle 1 will also be considered evaluable.)

<u>Evaluable Non-Target Disease Response</u>. Patients who have lesions present at baseline that are evaluable but do not meet the definitions of measurable disease, have received at least one cycle of therapy, and have had their disease re-evaluated will be considered evaluable for non-target disease. The response assessment is based on the presence, absence, or unequivocal progression of the lesions.

11.1.2 Disease Parameters

<u>Measurable disease</u>. Measurable lesions are defined as those that can be accurately measured in at least one dimension (longest diameter to be recorded) as ≥20 mm by chest x-ray or as ≥10 mm with CT scan, MRI, or calipers by clinical exam. All tumor measurements must be recorded in <u>millimeters</u> (or decimal fractions of centimeters).

Note: Tumor lesions that are situated in a previously irradiated area might or might not be considered measurable. *If the investigator thinks it appropriate to include them, the conditions under which such lesions should be considered must be defined in the protocol*.

Malignant lymph nodes. To be considered pathologically enlarged and measurable, a lymph node must be ≥15 mm in short axis when assessed by CT scan (CT scan slice thickness recommended to be no greater than 5 mm). At baseline and in follow-up, only the short axis will be measured and followed.

Non-measurable disease. All other lesions (or sites of disease), including small lesions (longest diameter <10 mm or pathological lymph nodes with ≥10 to <15 mm short axis), are considered non-measurable disease. Bone lesions, leptomeningeal disease, ascites, pleural/pericardial effusions, lymphangitis cutis/pulmonitis, inflammatory breast disease, and abdominal masses (not followed by CT or MRI), are considered as non-measurable.

Note: Cystic lesions that meet the criteria for radiographically defined simple cysts should not be considered as malignant lesions (neither measurable nor non-measurable) since they are, by definition, simple cysts.

'Cystic lesions' thought to represent cystic metastases can be considered as measurable lesions, if they meet the definition of measurability described above. However, if non-cystic lesions are present in the same patient, these are preferred for selection as target lesions.

Target lesions. All measurable lesions up to a maximum of 2 lesions per organ and 5 lesions in total, representative of all involved organs, should be identified as **target lesions** and recorded and measured at baseline. Target lesions should be selected on the basis of their size (lesions with the longest diameter), be representative of all involved organs, but in addition should be those that lend themselves to reproducible repeated measurements. It may be the case that, on occasion, the largest lesion does not lend itself to reproducible measurement in which circumstance the next largest lesion, which can be measured reproducibly should be selected. A sum of the diameters (longest for non-nodal lesions, short axis for nodal lesions) for all target lesions will be calculated and reported as the baseline sum diameters. If lymph nodes are to be included in the sum, then only the short axis is added into the sum. The baseline sum diameters will be used as reference to further characterize any objective tumor regression in the measurable dimension of the disease.

Non-target lesions. All other lesions (or sites of disease) including any measurable lesions over and above the 5 target lesions should be identified as **non-target lesions** and should also be recorded at baseline. Measurements of these lesions are not required, but the presence, absence, or in rare cases unequivocal progression of each should be noted throughout follow-up.

11.1.3 Methods for Evaluation of Measurable Disease

All measurements should be taken and recorded in metric notation using a ruler or calipers. All baseline evaluations should be performed as closely as possible to the beginning of treatment and never more than 4 weeks before the beginning of the treatment.

The same method of assessment and the same technique should be used to characterize each identified and reported lesion at baseline and during follow-up. Imaging-based evaluation is preferred to evaluation by clinical examination unless the lesion(s) being followed cannot be imaged but are assessable by clinical exam.

Clinical lesions: Clinical lesions will only be considered measurable when they are superficial (*e.g.*, skin nodules and palpable lymph nodes) and ≥10 mm diameter as assessed using calipers (*e.g.*, skin nodules). In the case of skin lesions, documentation by color photography, including a ruler to estimate the size of the lesion, is recommended.

Chest x-ray: Lesions on chest x-ray are acceptable as measurable lesions when they are clearly defined and surrounded by aerated lung. However, CT is preferable.

Conventional CT and MRI: This guideline has defined measurability of lesions on CT scan based on the assumption that CT slice thickness is 5 mm or less. If CT scans have slice thickness greater than 5 mm, the minimum size for a measurable lesion should be twice the slice thickness. MRI is also acceptable in certain situations (*e.g.* for body scans).

Use of MRI remains a complex issue. MRI has excellent contrast, spatial, and temporal resolution; however, there are many image acquisition variables involved in MRI, which greatly impact image quality, lesion conspicuity, and measurement. Furthermore, the availability of MRI is variable globally. As with CT, if an MRI is performed, the technical specifications of the scanning sequences used should be optimized for the evaluation of the type and site of disease. Furthermore, as with CT, the modality used at follow-up should be the same as was used at baseline and the lesions should be measured/assessed on the same pulse sequence. It is beyond the scope of the RECIST guidelines to prescribe specific MRI pulse sequence parameters for all scanners, body parts, and diseases. Ideally, the same type of scanner should be used and the image acquisition protocol should be followed as closely as possible to prior scans. Body scans should be performed with breath-hold scanning techniques, if possible.

PET-CT: At present, the low dose or attenuation correction CT portion of a combined PET-CT is not always of optimal diagnostic CT quality for use with RECIST measurements. However, if the site can document that the CT performed as part of a PET-CT is of identical diagnostic quality to a diagnostic CT (with IV and oral contrast), then the CT portion of the PET-CT can be used for RECIST measurements and can be used interchangeably with conventional CT in accurately measuring cancer lesions over time. Note, however, that the PET portion of the CT introduces additional data, which may bias an investigator if it is not routinely or serially performed.

Ultrasound: Ultrasound is not useful in assessment of lesion size and should not be used as a method of measurement. Ultrasound examinations cannot be reproduced in their entirety for independent review at a later date and, because they are operator dependent, it cannot be guaranteed that the same technique and measurements will be taken from one assessment to the next. If new lesions are identified by ultrasound in the course of the study, confirmation by CT or MRI is advised. If there is concern about radiation exposure at CT,

MRI may be used instead of CT in selected instances.

Endoscopy, Laparoscopy: The utilization of these techniques for objective tumor evaluation is not advised. However, such techniques may be useful to confirm complete pathological response when biopsies are obtained or to determine relapse in trials where recurrence following complete response (CR) or surgical resection is an endpoint.

Tumor markers: Tumor markers alone cannot be used to assess response. If markers are initially above the upper normal limit, they must normalize for a patient to be considered in complete clinical response. Specific guidelines for both CA-125 response (in recurrent ovarian cancer) and PSA response (in recurrent prostate cancer) have been published[49-51]. In addition, the Gynecologic Cancer Intergroup has developed CA-125 progression criteria which are to be integrated with objective tumor assessment for use in first-line trials in ovarian cancer[52].

Cytology, Histology: These techniques can be used to differentiate between partial responses (PR) and complete responses (CR) in rare cases (*e.g.*, residual lesions in tumor types, such as germ cell tumors, where known residual benign tumors can remain).

The cytological confirmation of the neoplastic origin of any effusion that appears or worsens during treatment when the measurable tumor has met criteria for response or stable disease is mandatory to differentiate between response or stable disease (an effusion may be a side effect of the treatment) and progressive disease.

FDG-PET: While FDG-PET response assessments need additional study, it is sometimes reasonable to incorporate the use of FDG-PET scanning to complement CT scanning in assessment of progression (particularly possible 'new' disease). New lesions on the basis of FDG-PET imaging can be identified according to the following algorithm:

a. Negative FDG-PET at baseline, with a positive FDG-PET at follow-up is a sign of PD based on a new lesion.
b. No FDG-PET at baseline and a positive FDG-PET at follow-up: If the positive FDG-PET at follow-up corresponds to a new site of disease confirmed by CT, this is PD. If the positive FDG-PET at follow-up is not confirmed as a new site of disease on CT, additional follow-up CT scans are needed to determine if there is truly progression occurring at that site (if so, the date of PD will be the date of the initial abnormal FDG-PET scan). If the positive FDG-PET at follow-up corresponds to a pre-existing site of disease on CT that is not progressing on the basis of the anatomic images, this is not PD.
c. FDG-PET may be used to upgrade a response to a CR in a manner similar to a biopsy in cases where a residual radiographic abnormality is thought to represent fibrosis or scarring. The use of FDG-PET in this circumstance should be prospectively described in the protocol and supported by disease-specific medical literature for the indication. However, it must be acknowledged that both approaches may lead to false positive CR due to limitations of FDG-PET and biopsy resolution/sensitivity.

Note: A ‘positive’ FDG-PET scan lesion means one which is FDG avid with an uptake

greater than twice that of the surrounding tissue on the attenuation corrected image.

11.1.4 **Response Criteria**

11.1.4.1 Evaluation of Target Lesions

Complete Response (CR): Disappearance of all target lesions. Any pathological lymph nodes (whether target or non-target) must have reduction in short axis to <10 mm.

Partial Response (PR): At least a 30% decrease in the sum of the diameters of target lesions, taking as reference the baseline sum diameters.

Progressive Disease (PD): At least a 20% increase in the sum of the diameters of target lesions, taking as reference the smallest sum on study (this includes the baseline sum if that is the smallest on study). In addition to the relative increase of 20%, the sum must also demonstrate an absolute increase of at least 5 mm. (Note: the appearance of one or more new lesions is also considered progressions).

Stable Disease (SD): Neither sufficient shrinkage to qualify for PR nor sufficient increase to qualify for PD, taking as reference the smallest sum diameters while on study.

11.1.4.2 Evaluation of Non-Target Lesions

Complete Response (CR): Disappearance of all non-target lesions and normalization of tumor marker level. All lymph nodes must be non-pathological in size (<10 mm short axis).

Note: If tumor markers are initially above the upper normal limit, they must normalize for a patient to be considered in complete clinical response.

Non-CR/Non-PD: Persistence of one or more non-target lesion(s) and/or maintenance of tumor marker level above the normal limits.

Progressive Disease (PD): Appearance of one or more new lesions and/or *unequivocal progression* of existing non-target lesions. *Unequivocal progression* should not normally trump target lesion status. It must be representative of overall disease status change, not a single lesion increase.

Although a clear progression of "non-target" lesions only is exceptional, the opinion of the treating physician should prevail in such circumstances, and the progression status should be confirmed at a later time by the review panel (or Principal Investigator).

11.1.4.3 Evaluation of Best Overall Response

The best overall response is the best response recorded from the start of the treatment

until disease progression/recurrence (taking as reference for progressive disease the smallest measurements recorded since the treatment started). The patient's best response assignment will depend on the achievement of both measurement and confirmation criteria.

Table 25: For Patients with Measurable Disease (*i.e.*, Target Disease)

<table>
<tr><th>Target Lesions</th><th>Non-Target Lesions</th><th>New Lesions</th><th>Overall Response</th><th>Best Overall Response when Confirmation is Required*</th></tr>
<tr><td>CR</td><td>CR</td><td>No</td><td>CR</td><td>≥4 wks. Confirmation**</td></tr>
<tr><td>CR</td><td>Non-CR/Non-PD</td><td>No</td><td>PR</td><td rowspan="3">≥4 wks. Confirmation**</td></tr>
<tr><td>CR</td><td>Not evaluated</td><td>No</td><td>PR</td></tr>
<tr><td>PR</td><td>Non-CR/Non-PD/not evaluated</td><td>No</td><td>PR</td></tr>
<tr><td>SD</td><td>Non-CR/Non-PD/not evaluated</td><td>No</td><td>SD</td><td>Documented at least once ≥4 wks. from baseline**</td></tr>
<tr><td>PD</td><td>Any</td><td>Yes or No</td><td>PD</td><td rowspan="3">no prior SD, PR or CR</td></tr>
<tr><td>Any</td><td>PD***</td><td>Yes or No</td><td>PD</td></tr>
<tr><td>Any</td><td>Any</td><td>Yes</td><td>PD</td></tr>
<tr><td colspan="5">* See RECIST 1.1 manuscript for further details on what is evidence of a new lesion.
** Only for non-randomized trials with response as primary endpoint.
*** In exceptional circumstances, unequivocal progression in non-target lesions may be accepted as disease progression.

Note: Patients with a global deterioration of health status requiring discontinuation of treatment without objective evidence of disease progression at that time should be reported as “symptomatic deterioration.” Every effort should be made to document the objective progression even after discontinuation of treatment.</td></tr>
</table>

Table 26: For Patients with Non-Measurable Disease (*i.e.*, Non-Target Disease)

Non-Target Lesions	New Lesions	Overall Response
CR	No	CR
Non-CR/non-PD	No	Non-CR/non-PD*
Not all evaluated	No	not evaluated
Unequivocal PD	Yes or No	PD
Any	Yes	PD

* ‘Non-CR/non-PD’ is preferred over ‘stable disease’ for non-target disease since SD is increasingly used as an endpoint for assessment of efficacy in some trials so to assign this category when no lesions can be measured is not advised

11.1.5 Duration of Response

Duration of overall response: The duration of overall response is measured from the time measurement criteria are met for CR or PR (whichever is first recorded) until the first date that recurrent or progressive disease is objectively documented (taking as reference for progressive disease the smallest measurements recorded since the treatment started).

The duration of overall CR is measured from the time measurement criteria are first met for CR until the first date that progressive disease is objectively documented.

Duration of stable disease: Stable disease is measured from the start of the treatment until the criteria for progression are met, taking as reference the smallest measurements recorded since the treatment started, including the baseline measurements.

12. STUDY OVERSIGHT AND DATA REPORTING / REGULATORY REQUIREMENTS

Adverse event lists, guidelines, and instructions for AE reporting can be found in Section 7.0 (Adverse Events: List and Reporting Requirements).

12.1 Study Oversight

This protocol is monitored at several levels, as described in this section. The Protocol Principal Investigator is responsible for monitoring the conduct and progress of the clinical trial, including the ongoing review of accrual, patient-specific clinical and laboratory data, and routine and serious adverse events; reporting of expedited adverse events; and accumulation of reported adverse events from other trials testing the same drug(s). The Protocol Principal Investigator and statistician have access to the data at all times through the CTMS web-based reporting portal.

For the Phase 1 portion of this study, all decisions regarding dose escalation/expansion/de-escalation require sign-off by the Protocol Principal Investigator through the CTMS/IWRS. In addition, for the Phase 1 portion, the Protocol Principal Investigator will have at least monthly, or more frequently, conference calls with the Study Investigators and the CTEP Medical Officer(s) to review accrual, progress, and adverse events and unanticipated problems.

All Study Investigators at participating sites who register/enroll patients on a given protocol are responsible for timely submission of data via Medidata Rave and timely reporting of adverse events for that particular study. This includes timely review of data collected on the electronic CRFs submitted via Medidata Rave.

All studies are also reviewed in accordance with the enrolling institution's data safety monitoring plan.

12.2 Data Reporting

Data collection for this study will be done exclusively through Medidata Rave. Access to the trial in Rave is granted through the iMedidata application to all persons with the appropriate roles assigned in the Regulatory Support System (RSS). To access Rave via iMedidata, the site user must have an active CTEP IAM account (https://eapps-ctep.nci.nih.gov/iam) and the appropriate Rave role (Rave CRA, Read-Only, or Site Investigator) on either the Corresponding Organization or Participating Organization roster at the enrolling site.

Upon initial site registration approval for the study in RSS, all persons with Rave roles assigned on the appropriate roster will be sent a study invitation e-mail from iMedidata. To accept the invitation, site users must log into the Select Login (https://login.imedidata.com/selectlogin) using their CTEP-IAM user name and password, and click on the "accept" link in the upper right-corner of the iMedidata page. Please note, site users will not be able to access the study in Rave until all required Medidata and study specific trainings are completed. Trainings will be in the form of electronic learnings (eLearnings), and can be accessed by clicking on the link in the upper right pane of the iMedidata screen.

Users that have not previously activated their iMedidata/Rave account at the time of initial site registration approval for the study in RSS will also receive a separate invitation from iMedidata to activate their account. Account activation instructions are located on the CTSU website, Rave tab under the Rave resource materials (Medidata Account Activation and Study Invitation Acceptance). Additional information on iMedidata/Rave is available on the CTSU members' website under the Rave tab or by contacting the CTSU Help Desk at 1-888-823-5923 or by e-mail at ctsucontact@westat.com.

Method

This study will be monitored by the Clinical Trials Monitoring Service (CTMS). Data will be submitted to CTMS at least once every two weeks via Medidata Rave (or other modality if approved by CTEP). Information on CTMS reporting is available at http://www.theradex.com/clinicalTechnologies/?National-Cancer-Institute-NCI-11. On-site audits will be conducted three times annually (one annual site visit and two data audits). For CTMS monitored studies, after users have activated their accounts, please contact the Theradex Help Desk at (609) 799-7580 or by email at CTMSSupport@theradex.com for additional support with Rave and completion of CRFs.

12.2.1 **Responsibility for Data Submission**

For ETCTN trials, it is the responsibility of the PI(s) at the site to ensure that all investigators at the ETCTN Sites understand the procedures for data submission for each ETCTN protocol and that protocol specified data are submitted accurately and in a timely manner to the CTMS via the electronic data capture system, Medidata Rave.

Data are to be submitted via Medidata Rave to CTMS on a real-time basis, but no less than once every 2 weeks. The timeliness of data submissions and timeliness in resolving data queries will be tracked by CTMS. Metrics for timeliness will be followed and assessed on a quarterly basis. For the purpose of Institutional Performance Monitoring, data will be considered delinquent if it is greater than 4 weeks past due.

Data from Medidata Rave and CTEP-AERS is reviewed by the CTMS on an ongoing basis as data is received. Queries will be issued by CTMS directly within Rave. The queries will appear on the Task Summary Tab within Rave for the CRA at the ETCTN to resolve. Monthly web-based reports are posted for review by the Drug Monitors in the IDB, CTEP. Onsite audits will be conducted by the CTMS to ensure compliance with

regulatory requirements, GCP, and NCI policies and procedures with the overarching goal of ensuring the integrity of data generated from NCI-sponsored clinical trials, as described in the ETCTN Program Guidelines, which may be found on the CTEP (http://ctep.cancer.gov/protocolDevelopment/electronic_applications/adverse_events.htm) and CTSU websites.

An End of Study CRF is to be completed by the PI, and is to include a summary of study endpoints not otherwise captured in the database, such as (for phase 1 trials) the recommended phase 2 dose (RP2D), and a description of any dose-limiting toxicities (DLTs). CTMS will utilize a core set of eCRFs that are Cancer Data Standards Registry and Repository (caDSR) compliant (http://cbiit.nci.nih.gov/ncip/biomedical-informatics-resources/interoperability-and-semantics/metadata-and-models). Customized eCRFs will be included when appropriate to meet unique study requirements. The PI is encouraged to review the eCRFs, working closely with CTMS to ensure prospectively that all required items are appropriately captured in the eCRFs prior to study activation. CTMS will prepare the eCRFs with built-in edit checks to the extent possible to promote data integrity.

CDUS data submissions for ETCTN trials activated after March 1, 2014, will be carried out by the CTMS contractor, Theradex. CDUS submissions are performed by Theradex on a monthly basis. The trial's lead institution is responsible for timely submission to CTMS via Rave, as above.

Further information on data submission procedures can be found in the ETCTN Program Guidelines (http://ctep.cancer.gov/protocolDevelopment/electronic_applications/adverse_events.htm).

12.3 Collaborative Agreements Language

The agent(s) supplied by CTEP, DCTD, NCI used in this protocol is/are provided to the NCI under a Collaborative Agreement (CRADA, CTA, CSA) between the Pharmaceutical Company(ies) (hereinafter referred to as "Collaborator(s)") and the NCI Division of Cancer Treatment and Diagnosis. Therefore, the following obligations/guidelines, in addition to the provisions in the "Intellectual Property Option to Collaborator" (http://ctep.cancer.gov/industryCollaborations2/intellectual_property.htm) contained within the terms of award, apply to the use of the Agent(s) in this study:

1. Agent(s) may not be used for any purpose outside the scope of this protocol, nor can Agent(s) be transferred or licensed to any party not participating in the clinical study. Collaborator(s) data for Agent(s) are confidential and proprietary to Collaborator(s) and shall be maintained as such by the investigators. The protocol documents for studies utilizing Agents contain confidential information and should not be shared or distributed without the permission of the NCI. If a copy of this protocol is requested by a patient or patient's family member participating on the study, the individual should sign a confidentiality agreement. A suitable model agreement can be downloaded from:

http://ctep.cancer.gov.

2. For a clinical protocol where there is an investigational Agent used in combination with (an) other Agent(s), each the subject of different Collaborative Agreements, the access to and use of data by each Collaborator shall be as follows (data pertaining to such combination use shall hereinafter be referred to as "Multi-Party Data”):

   a. NCI will provide all Collaborators with prior written notice regarding the existence and nature of any agreements governing their collaboration with NCI, the design of the proposed combination protocol, and the existence of any obligations that would tend to restrict NCI's participation in the proposed combination protocol.

   b. Each Collaborator shall agree to permit use of the Multi-Party Data from the clinical trial by any other Collaborator solely to the extent necessary to allow said other Collaborator to develop, obtain regulatory approval or commercialize its own Agent.

   c. Any Collaborator having the right to use the Multi-Party Data from these trials must agree in writing prior to the commencement of the trials that it will use the Multi-Party Data solely for development, regulatory approval, and commercialization of its own Agent.

3. Clinical Trial Data and Results and Raw Data developed under a Collaborative Agreement will be made available to Collaborator(s), the NCI, and the FDA, as appropriate and unless additional disclosure is required by law or court order as described in the IP Option to Collaborator (http://ctep.cancer.gov/industryCollaborations2/intellectual_property.htm). Additionally, all Clinical Data and Results and Raw Data will be collected, used and disclosed consistent with all applicable federal statutes and regulations for the protection of human subjects, including, if applicable, the *Standards for Privacy of Individually Identifiable Health Information* set forth in 45 C.F.R. Part 164.

4. When a Collaborator wishes to initiate a data request, the request should first be sent to the NCI, who will then notify the appropriate investigators (Group Chair for Cooperative Group studies, or PI for other studies) of Collaborator's wish to contact them.

5. Any data provided to Collaborator(s) for Phase 3 studies must be in accordance with the guidelines and policies of the responsible Data Monitoring Committee (DMC), if there is a DMC for this clinical trial.

6. Any manuscripts reporting the results of this clinical trial must be provided to CTEP by the Group office for Cooperative Group studies or by the principal investigator for non-Cooperative Group studies for immediate delivery to Collaborator(s) for advisory review and comment prior to submission for publication. Collaborator(s) will have 30 days from the date of receipt for review. Collaborator shall have the right to request that publication be delayed for up to an additional 30 days in order to ensure that Collaborator’s confidential

and proprietary data, in addition to Collaborator(s)'s intellectual property rights, are protected. Copies of abstracts must be provided to CTEP for forwarding to Collaborator(s) for courtesy review as soon as possible and preferably at least three (3) days prior to submission, but in any case, prior to presentation at the meeting or publication in the proceedings. Press releases and other media presentations must also be forwarded to CTEP prior to release. Copies of any manuscript, abstract and/or press release/ media presentation should be sent to:

Email: ncicteppubs@mail.nih.gov

The Regulatory Affairs Branch will then distribute them to Collaborator(s). No publication, manuscript or other form of public disclosure shall contain any of Collaborator's confidential/ proprietary information.

13. STATISTICAL CONSIDERATIONS

13.1 Study Design/Endpoints

This phase 1 trial will use a design involving four cohorts of patients with different degrees of liver dysfunction.

- The dose escalation rules used in this study are adapted from the standard up-and-down "3+3" design, and maintain the basic principles of that design. Normal, mild, moderate and severe liver dysfunction cohorts may be open concurrently. The design has been modified for this organ dysfunction study to eliminate waiting periods between dose levels as the clinical stability of patients with impaired hepatic function is frequently limited, and it is thus unreasonable to delay therapy for 2-3 weeks in this patient population. The disadvantage of this approach is that it may increase the number of patients who receive a dose that is subsequently found to be above the recommended dose level. However, the benefit is expected to outweigh this risk as this population of patients is small, has few or no standard therapeutic options, and these patients usually have a limited timeframe during which therapy can be safely administered.

- Although dose-finding will be carried out independently for each of the liver dysfunction groups, an ancillary constraint is imposed: accrual to Group D (severe) will occur one patient at a time (i.e., the second patient in a cohort will not be enrolled until the first patient completes cycle 1 and so on for each subsequent patient until the equivalent moderate dose is complete), and the dose recommended for a group with greater liver dysfunction cannot be greater than that for a group with a lesser dysfunction. While it is conceivable that patients with greater liver dysfunction might tolerate the study drug better than those with lesser dysfunction, it is considered very unlikely. Furthermore, the highest dose to be explored is no greater than the recommended dose for patients with normal liver function. Thus, the ancillary constraint can do no harm; it is intended to compensate in part for patient heterogeneity and yield more accurate final recommended doses than possible with independent dose escalation in the four liver dysfunction groups.

- At least 12 patients (1 per participating institution) will be entered into Group A (normal liver function). Patients in Group A are included in this study to obtain PK data in the same manner as for the patients with liver dysfunction. This group will also be followed for toxicity, but the definitions of recommended dose that are specific to patients with liver dysfunction will not be used.

- If a delay in completing one cohort occurs (*e.g.* severe liver dysfunction cohort has been completed enrollment), study data analysis and reporting will be considered for cohorts that have been completed. The enrollment for the remaining cohorts will continue concomitantly to data analysis.

- Toxicity will be graded according to the NCI CTCAE v4.0.0 and relationship to the study drug; results will be tabulated by liver dysfunction group. All patients who receive any amount of trametinib will be evaluable for toxicity, but patients who receive other than the prescribed dose and do not experience a DLT will be considered inevaluable for DLT. Patients who are not evaluable for DLT will be replaced. One exception is made for patients enrolled in the severe hepatic dysfunction cohort (Group D). As patients with severe hepatic dysfunction can deteriorate quickly due to their disease and poor organ functions, patients in this cohort who complete PK schedule at day 16 will be considered evaluable for DLT assessment.

Primary Endpoints:

- To provide appropriate dosing recommendations for patients with varying degree of hepatic dysfunction receiving trametinib (mild, moderate and severe).
- To establish the maximum tolerated dose (MTD) and dose-limiting toxicity (DLT) of trametinib in advanced cancer patients with varying degrees of hepatic dysfunction
- To characterize the PK profile of trametinib in advanced cancer patients with varying degrees of hepatic dysfunction.

Secondary Endpoints:

- To document the non-DLTs associated with the administration of trametinib in patients with varying degrees of hepatic dysfunction.
- To document any antitumor activity associated with trametinib treatment of patients enrolled on this study.
- To explore and characterize predictive biomarkers for individual cancer patients utilizing genomic sequencing technologies.

13.2 **Sample Size/Accrual Rate**

- A total of 12 patients will be accrued in the Normal Hepatic Function cohort. A minimum of 3 and a maximum of 12 patients will be accrued in the Mild Hepatic Dysfunction cohort (B). A minimum of 6 and a maximum of 22 patients will be accrued in the Moderate Hepatic Dysfunction cohort (C). A minimum of 6 and a maximum of 22 patients will be accrued in the Severe Hepatic Dysfunction cohort (D). In total this study will accrue a minimum of 27 and a maximum of 68 patients.

13.3 Stratification Factors

Patients will be stratified according to level of hepatic dysfunction as summarized in the table below (Table 27) and described in Section 5.1. Dose escalation and determination of the MTD will be carried out separately for each cohort or stratum. For Cohort A, no MTD or RP2D will need to be defined as this group has normal liver function. For Cohorts B, C and D, the 3+3 rule will be used to define the RP2D, such that for each Cohort, the highest level in which $\leq$ 1 out of 6 patients experienced DLT, at or below the maximum administered dose, would constitute the RP2D. If is $\leq$ 1 of 6 patients experienced DLTs at the maximum administered dose, then the maximum administered dose will be declared RP2D for that cohort.

Table 27: Defining criteria for the four cohorts based on hepatic function.

Group	Hepatic Function	Hepatic Function
A	Normal hepatic function	Bilirubin ≤ ULN<br>AST ≤ ULN
B	Mild hepatic dysfunction	B1: bilirubin ≤ ULN and AST > ULN<br>B2: ULN < bilirubin ≤ 1.5x ULN and any AST
C	Moderate hepatic dysfunction	1.5x ULN < bilirubin ≤ 3x ULN and any AST
D	Severe hepatic dysfunction	3x ULN < bilirubin ≤ 10x ULN and any AST

Further exploratory analysis may be performed using the clinical and pharmacokinetic data collected from this study based on patients' Childs-Pugh Classification.

13.4 Analysis of Secondary Endpoints

Summary statistics, such as the mean, median, counts and proportion, will be used to describe patients' clinical characteristics. Objective response to treatment will be assessed using the RECIST criteria 1.1. If no objective responses are observed in a disease-specific dose expansion cohort with a minimum of 10 patients evaluable for response, there is ≥89% probability to exclude a true objective response rate of ≥20%.

Predictors of clinical outcomes will be investigated using logistic regression, Cox proportional hazards regression and/or generalized estimating equations as appropriate. Potential predictors include clinical predictors and molecular correlates. Descriptive statistics and plotting of data will also be used to better understand potential relationships.

Frequency and severity of adverse events will be tabulated using counts and proportions detailing frequently occurring, serious and severe events of interest. Adverse events will be summarized using all adverse events experienced, although a sub-analysis may be conducted including only those adverse events in which the treating physician deems possibly, probably or definitely attributable to one or both study treatments. Attempts to model associations between pharmacokinetic data with toxicity profiles will be performed primarily using descriptive statistics; however, logistic regression may be used if warranted.

All analyses will be considered exploratory and inference will be performed with appropriate caution. For all statistical tests, two-sided tests will be performed and no p-value adjustment performed due to the exploratory nature of these tests. A p-value of 0.05 or less will be considered statistically significant.

REFERENCES

1. Donelli MG, Zucchetti M, Munzone E, et al: Pharmacokinetics of anticancer agents in patients with impaired liver function. Eur J Cancer 34:33-46, 1998
2. Sun H, Frassetto L, Benet LZ: Effects of renal failure on drug transport and metabolism. Pharmacol Ther 109:1-11, 2006
3. Administration USFaD: Guidance for Industry Drug-Induced Liver Injury: Premarketing Clinical Evaluation, http://mobile.pharmacytimes.com/publications/issue/2012/July2012/Organ-Dysfunction-Treating-Patients-With-CKD-and-Liver-Disease - sthash.NOicujiS.dpuf, 2013
4. Egorin M: The effects of organ dysfunction on drug dosing. Clin Adv Hematol Oncol 4:116-8, 2006
5. Mano MS, Cassidy J, Canney P: Liver metastases from breast cancer: management of patients with significant liver dysfunction. Cancer Treat Rev 31:35-48, 2005
6. Kamath PS, Wiesner RH, Malinchoc M, et al: A model to predict survival in patients with end-stage liver disease. Hepatology 33:464-70, 2001
7. Administration. USFaD: FDA approves two drugs, companion diagnostic test for advanced skin cancer [Press release]. Retrieved from http://www.fda.gov/NewsEvents/Newsroom/PressAnnouncements/ucm354199.htm., 2013
8. Administration. USFaD: FDA approves Mekinist in combination with Tafinlar for advanced melanoma [Press release]. http://www.fda.gov/NewsEvents/Newsroom/PressAnnouncements/ucm381159.htm., 2014
9. Flaherty KT, Robert C, Hersey P, et al: Improved survival with MEK inhibition in BRAF-mutated melanoma. N Engl J Med 367:107-14, 2012
10. Gopal YN, Deng W, Woodman SE, et al: Basal and treatment-induced activation of AKT mediates resistance to cell death by AZD6244 (ARRY-142886) in Braf-mutant human cutaneous melanoma cells. Cancer Res 70:8736-47, 2010
11. Ebi H, Corcoran RB, Singh A, et al: Receptor tyrosine kinases exert dominant control over PI3K signaling in human KRAS mutant colorectal cancers. J Clin Invest 121:4311-21, 2011
12. Engelman JA, Chen L, Tan X, et al: Effective use of PI3K and MEK inhibitors to treat mutant Kras G12D and PIK3CA H1047R murine lung cancers. Nat Med 14:1351-6, 2008
13. Hoeflich KP, O'Brien C, Boyd Z, et al: In vivo antitumor activity of MEK and phosphatidylinositol 3-kinase inhibitors in basal-like breast cancer models. Clin Cancer Res 15:4649-64, 2009
14. Tolcher AW, J.C. Bendell, A. Patnaik, et al. : A phase Ib study of the MEK inhibitor GSK1120212 combined with gemcitabine in patients with solid tumors: Interim results, J Clin Oncol, 2011
15. Bendell J, P. LoRusso, E. Kwak, et al. : Clinical combination of the MEK inhibitor GDC-0973 and the PI3K inhibitor GDC-0941: A first-in-human phase Ib study in patients with advanced solid tumors, AACR Meeting Abstracts., 2011
16. GlaxoSmithKline: Investigator's Brochure. GSK1120212 (trametinib). 2013
17. Gilmartin AG, Bleam MR, Groy A, et al: GSK1120212 (JTP-74057) is an inhibitor of MEK activity and activation with favorable pharmacokinetic properties for sustained in vivo pathway inhibition. Clin Cancer Res 17:989-1000, 2011
18. Yamaguchi T, Kakefuda R, Tajima N, et al: Antitumor activities of JTP-74057 (GSK1120212), a novel MEK1/2 inhibitor, on colorectal cancer cell lines in vitro and in vivo. Int J Oncol 39:23-31, 2011

19. Investigator's Brochure. (2012a). GSK1120212. GlaxoSmithKline. Version 04. September 5, 2012.
20. Infante JR, L.A. Fecher, S. Nallapareddy, et al. : Safety and efficacy results from the first-in-human study of the oral MEK 1/2 inhibitor GSK1120212, ASCO, J Clin Oncol. , 2010
21. Messersmith WA, G.S. Falchook, L.A. Fecher, et al. : Clinical activity of the oral MEK1/MEK2 inhibitor GSK1120212 in pancreatic and colorectal cancer. . ASCO, J Clin Oncol 29 suppl 4; abstr 246, 2011
22. GlaxoSmithKline: MEKINIST Package Insert, May 2013
23. Borthakur G, J.M. Foran, T. Kadia, et al: GSK1120212, a MEK1/MEK2 inhibitor, demonstrates acceptable tolerability and preliminary activity in a dose rising trial In subjects with AML and other hematologic malignancies. , Blood (ASH Annual Meeting Abstracts), 2010
24. Blumenschein G, Smit EF, Planchard D, et al. : MEK114653: A randomized, multicenter, phase II study to assess efficacy and safety of trametinib (T) compared with docetaxel (D) in KRAS-mutant advanced non-small cell lung cancer (NSCLC). , J Clin Oncol., 2013
25. Infante J, Somer BG, Park JO, et al. : A randomized, double-blind, placebo-controlled trial of trametinib, a MEK inhibitor, in combination with gemcitabine for patients with untreated metastatic adenocarcinoma of the pancreas. , ASCO, J Clin Oncol. , 2013
26. Investigator's Brochure. (2012a). GSK1120212. GlaxoSmithKline. Version 04. September 5, 2012.
27. Kurzrock R, Patnaik A, Rosenstein L, et al. : Phase I dose-escalation of the oral MEK1/2 inhibitor GSK1120212 (GSK212) dosed in combination with the oral AKT inhibitor GSK2141795 (GSK795). , ASCO, J Clin Oncol., 2011
28. Abe H KS, Hayakawa K et al. : Discovery of a highly potent and selective MEK inhibitor: GSK1120212 (JTP-74057 DMSOSolvate). . ACS Med Chem Lett 2:320-4, 2011
29. Jing J, Greshock J, Holbrook JD, et al: Comprehensive predictive biomarker analysis for MEK inhibitor GSK1120212. Mol Cancer Ther 11:720-9, 2012
30. Infante JR, Fecher LA, Falchook GS, et al: Safety, pharmacokinetic, pharmacodynamic, and efficacy data for the oral MEK inhibitor trametinib: a phase 1 dose-escalation trial. Lancet Oncol 13:773-81, 2012
31. Falchook GS, Lewis KD, Infante JR, et al: Activity of the oral MEK inhibitor trametinib in patients with advanced melanoma: a phase 1 dose-escalation trial. Lancet Oncol 13:782-9, 2012
32. Sausville EA: Promises from trametinib in RAF active tumors. N Engl J Med 367:171-2, 2012
33. Bekaii-Saab T, Phelps MA, Li X, et al: Multi-institutional phase II study of selumetinib in patients with metastatic biliary cancers. J Clin Oncol 29:2357-63, 2011
34. Farley J, Brady WE, Vathipadiekal V, et al: Selumetinib in women with recurrent low-grade serous carcinoma of the ovary or peritoneum: an open-label, single-arm, phase 2 study. Lancet Oncol 14:134-40, 2013
35. Kim KB, Kefford R, Pavlick AC, et al: Phase II study of the MEK1/MEK2 inhibitor Trametinib in patients with metastatic BRAF-mutant cutaneous melanoma previously treated with or without a BRAF inhibitor. J Clin Oncol 31:482-9, 2013
36. Karen K MJ, Leighl NB, Barlesi F, Zalcman G, Gordon MS, Reckamp KL, Gandara DR, Gomez-Roca CA, Bennouna J, Chul Cho B, Park K, Infante JR, Richards DA, Wu Y, Schramek DJ, Cox DS, Gardner OS, Peddareddigari VGR, Blumenschein GR.: Oral MEK1/MEK2 inhibitor trametinib (GSK1120212) in combination with pemetrexed for KRAS-mutant and wild-type (WT) advanced non-small cell lung cancer (NSCLC): A phase I/Ib trial., ASCO, J Clin

Oncol 31, 2013 2013
37. Gandara DR HS, Blumenschein GR, et al.: Oral MEK1/MEK2 inhibitor trametinib (GSK1120212) in combination with docetaxel in KRAS-mutant and wild-type (WT) advanced non-small cell lung cancer (NSCLC): A phase I/Ib trial., ASCO, J Clin Oncol 31, 2013 ASCO Annual Meeting 2013
38. Infante JR SB, Park JO, Li CP, Scheulen ME, Kasubhai SM, Oh DY, Liu Y, Lahiri S, Steplewski K, Le NT: A randomized, double-blind, placebo-controlled trial of trametinib, a MEK inhibitor, in combination with gemcitabine for patients with untreated metastatic adenocarcinoma of the pancreas. J Clin Oncol 30 (suppl34; abstract 291), 2013
39. Casey M GT, Hamid M, et al.: GSK1120212 Investigator's Brochure, 2012, September 5th
40. Infante JR, Papadopoulos KP, Bendell JC, et al: A phase 1b study of trametinib, an oral Mitogen-activated protein kinase kinase (MEK) inhibitor, in combination with gemcitabine in advanced solid tumours. Eur J Cancer, 2013
41. Balagula Y, Barth Huston K, Busam KJ, et al: Dermatologic side effects associated with the MEK 1/2 inhibitor selumetinib (AZD6244, ARRY-142886). Invest New Drugs 29:1114-21, 2011
42. Lacouture ME, Anadkat MJ, Bensadoun RJ, et al: Clinical practice guidelines for the prevention and treatment of EGFR inhibitor-associated dermatologic toxicities. Support Care Cancer 19:1079-95, 2011
43. Macconaill LE, Garraway LA: Clinical implications of the cancer genome. J Clin Oncol 28:5219-28, 2010
44. de Bono JS, Ashworth A: Translating cancer research into targeted therapeutics. Nature 467:543-9, 2010
45. Liang S CP, Ma Z , et al.: A community-based program for personalized cancer care using next-generation sequencing (NGS). ASCO, J Clin Oncol 31, 2013, 2013
46. Gunn S RX, Smith LS, et al.: Routine molecular profiling of solid tumors in a community cancer center's genomic pathology laboratory., ASCO, J Clin Oncol 31, 2013
47. Bedard PL OA, Tsao MS, Leighl NB, Shepherd FA, Chen EX, Tannock I, Krzyzanowska MK, Dhani NC, Clarke B, Berman HK, Serra S, Craddock KJ, Chadwick D, Zhang T, Sukhai MA, Yu C, Hansen AR, Kamel-Reid S, Siu LL.: Princess Margaret Cancer Centre (PMCC) Integrated Molecular Profiling in Advanced Cancers Trial (IMPACT) using genotyping and targeted next-generation sequencing (NGS). ASCO, J Clin Oncol 31, 2013, 2013
48. Eisenhauer EA, Therasse P, Bogaerts J, et al: New response evaluation criteria in solid tumours: revised RECIST guideline (version 1.1). Eur J Cancer 45:228-47, 2009
49. Rustin GJ, Quinn M, Thigpen T, et al: Re: New guidelines to evaluate the response to treatment in solid tumors (ovarian cancer). J Natl Cancer Inst 96:487-8, 2004
50. Bubley GJ, Carducci M, Dahut W, et al: Eligibility and response guidelines for phase II clinical trials in androgen-independent prostate cancer: recommendations from the Prostate-Specific Antigen Working Group. J Clin Oncol 17:3461-7, 1999
51. Scher HI, Halabi S, Tannock I, et al: Design and end points of clinical trials for patients with progressive prostate cancer and castrate levels of testosterone: recommendations of the Prostate Cancer Clinical Trials Working Group. J Clin Oncol 26:1148-59, 2008
52. Vergote I, Rustin GJ, Eisenhauer EA, et al: Re: new guidelines to evaluate the response to treatment in solid tumors [ovarian cancer]. Gynecologic Cancer Intergroup. J Natl Cancer Inst 92:1534-5, 2000

APPENDIX A PERFORMANCE STATUS CRITERIA

ECOG Performance Status Scale		Karnofsky Performance Scale	
Grade	Descriptions	Percent	Description
0	Normal activity. Fully active, able to carry on all pre-disease performance without restriction.	100	Normal, no complaints, no evidence of disease.
		90	Able to carry on normal activity; minor signs or symptoms of disease.
1	Symptoms, but ambulatory. Restricted in physically strenuous activity, but ambulatory and able to carry out work of a light or sedentary nature (*e.g.*, light housework, office work).	80	Normal activity with effort; some signs or symptoms of disease.
		70	Cares for self, unable to carry on normal activity or to do active work.
2	In bed <50% of the time. Ambulatory and capable of all self-care, but unable to carry out any work activities. Up and about more than 50% of waking hours.	60	Requires occasional assistance, but is able to care for most of his/her needs.
		50	Requires considerable assistance and frequent medical care.
3	In bed >50% of the time. Capable of only limited self-care, confined to bed or chair more than 50% of waking hours.	40	Disabled, requires special care and assistance.
		30	Severely disabled, hospitalization indicated. Death not imminent.
4	100% bedridden. Completely disabled. Cannot carry on any self-care. Totally confined to bed or chair.	20	Very sick, hospitalization indicated. Death not imminent.
		10	Moribund, fatal processes progressing rapidly.
5	Dead.	0	Dead.

APPENDIX B CHILD-PUGH Classification (CPC) of Liver Dysfunction

CPC score is calculated from the sum of the points for each CPC criteria:

**CPC Classification**	**Level of dysfunction**	**Score**
A	Mild	5-6
B	Moderate	7-9
C	Severe	≥ 10

	**Points**		
**CPC Criteria**	**1**	**2**	**3**
Encephalopathy grade (see table below)	0	1 or 2	3 or 4
Ascites	absent	asymptomatic	Requiring intervention
Serum bilirubin, mg/dL	< 2	2 to 3	> 3
Serum albumin, g/dL	> 3.5	2.8 to 3.5	< 2.8
Prothrombin time, sec prolonged	< 4	4 to 6	> 6

**Encephalopathy grade**	**Definition (EEG required for Gr. 2,3,4)**
0	Normal consciousness, personality, neurological exam
1	Restless, sleep disturbed, irritable/agitated, tremor, impaired handwriting
2	Lethargic, time-disoriented, inappropriate, asterixis, ataxia, slow triphasic waves on EEG
3	Somnolent, stuporous, place-disoriented, hyperactive reflexes, rigidity, slower waves on EEG
4	Unrousable coma, no personality/behavior, decerebrate, slow 2-3 cps delta activity on EEG

CPC should be calculated at baseline and prior to each treatment cycle.

APPENDIX C DATA MANAGEMENT GUIDELINES

Data Submission Schedule
This study will be monitored by the Clinical Trials Monitoring Service (CTMS). Information on CTMS reporting is available at http://www.theradex.com/CTMS. Data will be entered into the Medidata RAVE system by each participating site.

For all consortium sites (*except* LAO-11030):
Regulatory Requirements:

- A copy of the Site Participant's List/Delegation Log and Training Log
- Consent forms must be reviewed and pre-approved by the PM Phase 1 Consortium Central Office prior to submission to the local ethics board (REB/IRB).
- *Confirmation* of maintenance of the following documents at each institution must be made in writing to the Central Office Study Coordinator (listed on the protocol cover page) at Princess Margaret Cancer Centre:
    - All investigators must have a valid NCI investigator number
    - An up-to-date, signed and dated CV on file for all investigators
    - Laboratory certification/accreditation and normal ranges
    - Confirmation that all investigators have undergone training in the Protection of Human Research Subjects. It is preferred that other staff involved in the trial also undergo such training.
    - A current membership list of the ethics board
    - OHRP assurance number

For LAO-11030 consortium sites only:
Regulatory Requirements

- Please submit all required documents (#1-#8 below) to the PM Phase 1 Consortium Central Office (study coordinator on protocol face page).
- Consent forms must be reviewed and pre-approved by the Central Office prior to submission to the local ethics board (REB/IRB).
- All investigators must have a valid NCI investigator number on file with the PM Phase 1 Consortium Central Office.
- The following documents must be on file with the PM Phase 1 Consortium Central Office:
    1. A completed Qualified Investigator Undertaking for each Principal Investigator (Canadian sites only)
    2. An up-to-date, signed and dated CV on file for all investigators
    3. Laboratory certification/accreditation and normal ranges
    4. Confirmation that all investigators have undergone training in the Protection of Human Research Subjects is required. It is preferred that other staff involved in the trial also undergo such training
    5. A current membership list of the ethics board
    6. A Site Participant's List/Delegation Log and Training Log
    7. A copy of the initial approval letter from the ethics board for the trial
    8. OHRP assurance numbers, if applicable for each institution
- Continuing approval will be obtained at least yearly until follow-up on patients is completed and no further data is being obtained for research purposes.

Additional monitoring will be done by the Central Office Coordinator (listed on the protocol cover page) at Princess Margaret Cancer Centre. Source documentation is required to be forwarded at the timelines below.

Source Document Submission Schedule

**Case Report Form**	**Source Document Submission**
Eligibility Checklist	At the time of registration
Baseline	Within 2 weeks of on study date
On Treatment	Within 24 hrs of completion of course 1: Drug administration, lab values and AE pages<br>The rest of the source within 2 week of study visit
	Further courses within 2 weeks of study visit
Off Study	Within 2 week of the off study visit
Follow-up	Within 2 week of the follow-up visit<br>If SAE, including death, occurs within 30 days of last dose, this constitutes a reportable adverse event and it should be reported according to CTEP-AERS guidelines

Submission of Source Documentation for LAO-11030 consortium sites only

- De-identified source documentation will be submitted via fax or email to the PM Phase 1 Consortium Central Office (study coordinator on protocol face page) as outlined in the table above under Source Document Submission.

Verifying Data

- Source documentation will be submitted to the PM Phase 1 Consortium Central Office such as scans, progress notes, nursing notes, blood work, pathology reports etc. All patient names, hospital numbers or other identifying information will be removed prior to being sent to the Central Office and the documents labelled with patient initials, study number and the protocol number.

Monitoring

- Central data monitoring will take place throughout the trial at the Central Office. Query letters will be issued by Central Office staff that performs the monitoring. Signed query letters acknowledging completion will be returned to the Central Office study coordinator within two weeks of receipt.
- On-site monitoring may be performed at some participating sites.
- Clinical Trial Monitoring Service (CTMS) will also monitor data and issue queries.

Quality Assurance

- Quality review of studies will be performed at regular intervals.

Patient Registration

- Refer to section 4 of the protocol

APPENDIX D PK SAMPLING GUIDELINES

PK studies will be done to assess the biodistribution of trametinib.

Plasma concentrations of trametinib will be measured under the direction of GSK.

Specimen Collection and Analysis Details are provided in the "Pharmacokinetic Laboratory Manual" supplied by the Coordinating Central Office. Note: This will be disseminated with the clinical protocol and is also available to investigators on request from the coordinating center.

Specimen Collection

The blood samples will be collected into 2 mL K2-EDTA vacutainers for all time points except Day 15 predose and 2 hours. At those two points, blood samples will be collected into 5 mL K2-EDTA vacutainers due to extra plasma protein binding measurement. Fill all blood tube completely and mix immediately by gently inverting the tube 8-10 times. Place collected blood on wet ice *immediately* after mixing with EDTA.

Specimen Collection Schedule (an example of timepoints of collection is listed below).

Sample Number	Day of Collection	Planned Collection Time (hours)
1*	15	0 (Pretreatment)
2	15	0.5
3	15	1.0
4*	15	2.0
5	15	3.0
6	15	4.0
7	15	6.0
8	15	10
9	16	24

* 5mL of blood should be collected to generate 1.5 mL of plasma samples for pharmacokinetics and plasma protein binding analysis.

Specimen Processing Procedures

Blood samples stored on wet ice will be centrifuged *within 1 hour of collection* at approximately 1600 g for approximately 15 minutes under chilled conditions (approximately 4°C). For all time points except Day 15 predose and 2 hours, the resulting plasma will be transferred immediately into appropriately labeled 1.0-mL Matrix TrackMate ScrewTop tubes for PK analysis. For Day 15 predose and 2 hour time points, aliquot of 1.5 mL of plasma samples will be transferred immediately into labeled 3-mL Nunc tubes for plasma protein binding measurement; the remaining plasma samples (approximately 0.5 mL) will be transferred immediately to labeled 1.0 mL Matrix TrackMate ScrewTop tubes for PK analysis. All plasma samples will be placed on wet ice *immediately* after processing. Store samples frozen at minus 20°C (-20°C) or lower within 60 minutes of the sampling time. Ship frozen to Covance CLS.

Note: Do not fill the tube more than half full.

Specimen Shipping Instructions

Ship Pharmacokinetic samples to:

Sample Management-Bioanalytical (Rm 1S 160)
Covance Laboratory Inc.
3301 Kinsman Boulevard
Madison, WI 53704-2523
Madison.SA@Covance.com
Tel 608.241.4471
FAX 608.242.7978

Contact Information:

Mark Hoffman
Email: mark.hoffmann@covance.com
Phone: 608.230.1762

APPENDIX E PATIENT MEDICATION DIARY

Study Number: __________________ Cycle: ____________

Please record the time that you take your study medications. Return this diary, the medication bottles (even if empty or unopened), and any unused tablets at your next clinic visit and make sure you are given another diary.

Store your study medication in the original bottle in the refrigerator (2°C to 8°C). Temperature excursions should be limited to the time it takes for you to transport your study medication inn from the clinic/hospital to your home refrigerator.

Trametinib comes in a tablets form and should be taken by mouth every day. You should take trametinib at least 1 hour prior to or 2 hours after a meal.

Day	Date (mm/dd/yy)	Dose (mg)	Time taken	Day	Date (mm/dd/yy)	Dose (mg)	Time Taken
**1**				**15**			
**2**				**16**			
**3**				**17**			
**4**				**18**			
**5**				**19**			
**6**				**20**			
**7**				**21**			
**8**				**22**			
**9**				**23**			
**10**				**24**			
**11**				**25**			
**12**				**26**			
**13**				**27**			
**14**				**28**			

(Date) (Signature of Participant)

____________ ________________________
